# Theory-based evaluation of three research–practice partnerships designed to deliver novel, sustainable collaborations between adult social care research and practice in the UK: a research protocol for a ‘layered’ contributions analysis and realist evaluation

**DOI:** 10.1136/bmjopen-2022-068651

**Published:** 2022-11-25

**Authors:** Juliette Malley, Annette Bauer, Annette Boaz, Hannah Kendrick, Martin Knapp

**Affiliations:** 1 Care Policy and Evaluation Centre, The London School of Economics and Political Science, London, UK; 2 Department of Health Services Research and Policy, London School of Hygiene & Tropical Medicine, London, UK

**Keywords:** health policy, organisational development, quality in health care

## Abstract

**Introduction:**

Research–practice partnerships (RPPs) are long-term collaborations between research and practice that aim to conduct research that can be used to make practice-based improvements. They intentionally bring together diverse experience in decision making and seek to shift power dynamics so that all partners have a say. The Creating Care Partnerships project aims to explore whether the RPP approach developed within the US educational context can be successfully applied to the English care home context. The project involves a programme of codesign, implementation and evaluation within three case study sites. This protocol set outs the aims, research design and governance of the evaluation.

**Methods and analysis:**

The evaluation takes a theory-based approach to explore how, why and in what circumstances RPPs in the care home context contribute to enhancing research and research use in local care homes and informing wider improvement efforts. A mixed-methods design will be used for each case study, including semistructured interviews, observations of RPP events and meetings, an online survey, activity diary and review of local data and documents. Data collection will proceed in waves, with the theory of change (ToC) being continually refined and used to guide further data collection and analysis. Insights will be drawn using Contribution Analysis, Realist Evaluation and systems perspectives to assess the contribution made by the case study sites to achieving outcomes and the influence of contextual factors. Economic consequences will be identified through the ToC, using a narrative economic analysis to assess costs, consequences and value for money.

**Ethics and dissemination:**

The study has undergone ethics review by HRA Research Ethics Committee. It does not pose major ethical issues. A final report will be published and articles will be submitted to international journals.

**STRENGTHS AND LIMITATIONS OF THIS STUDY**

- A theory-based approach allows for greater explanation of how RRPs work, while appreciating the complexity and non-linearity of implementation.
- The use of mixed methods allows us to draw on the strengths of different methods, improving the credibility of evaluation findings.
- An economic evaluation will allow policy makers and funders to make evidence-based decisions about the value of further investment in research–practice partnerships for the care home sector.
- The evaluation period may not be long enough to capture the extent to which outcomes have been achieved within either local care homes or the wider care ecosystem.
- Theory-based approaches, including contributions analysis and realist evaluation that are used here, are not as well established as other approaches for establishing effectiveness. This study may draw criticism for not being as rigorous as experimental approaches.

## Introduction

An important question for adult social care (ASC) is how investment in research can be optimised to support improvements for people drawing on social care services. Despite significant investment and a growing evidence base,^1^ the evidence produced is in general poorly used by the ASC practice community (eg, social care commissioners, providers and the workforce).^2^ There is a growing interest in approaches that focus on building relationships and stronger links between those who produce research, those who use research and the intended beneficiaries to complement and augment existing efforts and infrastructure investments.^3–5^ The Creating Care Partnerships (CCP) project aims to redesign for the care home context and implement a promising approach called research–practice partnerships (RPPs) in three sites across England.^6^ Reflecting the central place learning has in the CCP project, it also includes an evaluation of the new RPPs. This paper describes the evaluation protocol.

RPPs are a specific form of partnership that offer a different way of producing and mobilising research that fundamentally challenges the status quo. They are long-term collaborations between research and practice communities that aim to bring about real-world change through the use of research evidence. RPPs can vary in scope and size and may have different strengths, but not all research–practice collaborations are RPPs.^7 8^ To be an RPP, collaborative efforts must extend beyond the life of a single research study or project and must engage with research as a core activity. Another feature of RPPs is the intentional integration of expertise from two communities—practice and research—that are often disconnected. Relatedly, RPPs engage in activities to shift power relations to ensure everyone has a say in the research endeavours; people from practice communities are involved from the outset, and both communities contribute equitably to shaping the direction of the work and supporting the use of what is learnt from the research.^8^ Although a substantial corpus of research has developed that describes the core principles of an RPP with lessons for those seeking to reproduce it (see NNERPP RPP knowledge clearinghouse https://nnerpp.rice.edu/rpp-knowledge-clearinghouse/, William T. Grant microsite https://rpp.wtgrantfoundation.org/), questions remain around what effective partnering looks like.^8 9^ How well RPPs meet their goals and the conditions that support or hinder their progress are seen as pressing issues for research.^8 10 11^

The main question for the CCP evaluation is whether this approach, which has been developed within the US educational context, can be successfully adapted for and implemented within the English care home context with similarly positive results in terms of driving improvements in practice and in the well-being of recipients of social care services. The primary aim of the CCP evaluation is therefore to provide evidence about the effectiveness of RPPs in the care home context; but, with a view to ensuring a legacy from the project, a second aim is to gather evidence about how to implement and sustain the approach so it can be reproduced elsewhere. The English care home context is very different to the US education context—a key difference being the lack of professionalisation and lower educational attainment of the majority of the care home workforce compared with educators, but there are also differences in the higher education contexts between the two countries and the research and innovation infrastructure. We expect this evaluation to deepen understanding of the way in which local conditions affect how RPPs function, the kinds of strategies they need to leverage to enact the RPP guiding principles and, possibly, what RPPs look like, with lessons for the international RPP community. Given the economic context and existing investments in research and knowledge mobilisation, a third aim of the evaluation is to understand the desirability of further investment in RPPs given the costs and the value that flows from the investment. Since the question of the economic value of RPPs is only beginning to be considered,^12^ this element of the evaluation is novel and will contribute to developing schemas for assessing value.

The CCP codesign work has produced a set of guiding principles for RPPs operating in the care home context that will be operationalised in different ways by each new RPP. Reflecting the strongly theoretical and complex nature of RPPs, our evaluation perspective is theory-based and draws on a system’s perspective.^13^ It addresses the following questions (and subquestions):

1. How, why and in what circumstances do RPPs in the care home context contribute to enhancing research and research use in local care homes and informing wider care home improvement efforts?
  - To what extent have the main outcomes been achieved?
  - How significant is the contribution of the CCP partnership to the main outcomes, given other factors?
  - How, why and in what circumstances do the CCP partnerships contribute to each outcome?
  - To what extent is the way the CCP partnerships operate consistent with the RPP approach?
2. What are the costs of delivering RPPs in the care home context, and are they good value for money?

It is not yet standard practice to publish protocols for evaluations of the kind outlined here. Our intention in publishing this protocol is to increase transparency in our methods and encourage discussion around them.

## Theoretical framework

We use the evidence-based framework developed by Henrick *et al*^10^ and adapted to the care home context as a framework for the evaluation. It identifies five dimensions of outcomes for successful RPPs: (1) building trust and cultivating partner relationships, (2) producing relevant research that is used, (3) supporting the practice organisation in achieving its goals, (4) producing knowledge that can inform social care practice improvements more broadly and (5) building the capacity of participants to engage in the partnership work.^9^ This framework provides a focus for measurement of RPP effectiveness and the integration of findings across the sites, but it can also inform the development and sustainability of such partnerships and theories of change.^14–16^

A key strength of the framework is that it is flexible enough to allow multiple theories to inform the evaluation, which existing research indicates will be necessary to understand how and why RPPs work. RPPs have been conceptualised in a variety of ways,^11 15^ but there is a consensus around the notion of RPPs as engaged in joint work at boundaries.^17 18^ In addition to theories of boundary infrastructure (including boundary spanners, practices and objects), scholars have drawn on organisational theories (including absorptive capacity, organisational learning and organisational routines) to explain how RPPs successfully produce and use research that improves practice within the practice organisation and has wider sectoral impacts.^17–19^ Since working across boundaries is challenging,^11^ many of the conceptual contributions also focus on relational aspects, including building trust, redistributing power, conflict and consensus, identity and role negotiation, and leadership.^18 20–22^ Theoretical contributions that help to analyse different types of power and how they are distributed^23^ and how new identities are formed or resisted^24^ may prove useful.

Evidence from existing RPPs suggests that it takes time for them to become productive and embedded within the wider higher education and practice ecosystems.^14^ As RPPs start to have an impact on and beyond the partnering organisations it may be useful to conceptualise them as ‘events in systems’.^25^ This perspective draws attention to the fact that RPPs are not neutral additions to the ecosystem but through their intention to have broader impact on care practice and how research is done, they challenge the status quo and in complex ways will interact with, shape and be shaped by these wider contexts. Understanding how the RPP and other players in the wider ecosystem (eg, higher education institutions, funding bodies, local authorities) interact with each other over time is key to understanding the potential for sustainability and spread of RPPs, as to endure RPPs must become resilient to shocks from within (eg, organisational turnover, conflicts and competing organisational norms) and, crucially, shocks from outwith the partnership (eg, changes in policy, economic shocks, changes in funding).^18^

## Design and methods for the theory-based evaluation

The evaluation is theory based and employs a multiple case study design with longitudinal data collection. There are various approaches to theory-based evaluation, which differ in their methods for constructing a valid theory of change (ToC) or programme theory and the types of questions for which they are designed to respond.^26–30^ In this evaluation, we use contribution analysis, and realist evaluation sequentially as ‘layered tactics’ to address different subquestions.^31^

The contribution analysis lens addresses whether the RPPs make a meaningful contribution to enhancing research and research use in care homes and the wider system (1b), by taking into account other factors and rival explanations.^28 32 33^ It produces credible causal claims about the contribution RPPs make to observed outcomes allowing us to draw conclusions about whether RPPs are a promising approach in the English care home context. By contrast through realist methods, we can probe in greater depth the different ways in which the CCP partnerships may implement the RPP approach, the circumstances that may affect the choices they make and the outcomes observed (1c). Realist evaluation explores causality through developing and testing programme theories as context–mechanism–outcome (CMO) configurations, which explain how and why RPPs might trigger different change mechanisms across different contexts to achieve (or not achieve) outcomes.^30 34^ The three partnerships–Research and Practice Development Care Partnership in north-west England, Care and Research North East, and Lancashire partnership–were selected through an open competition to offer contrasting situations. They differ in the size, scope, types of partners and local context, which will allow us to explore conducive or inhibiting contexts for RPPs.^35^ Should it prove useful for mapping the complexity of the RPPs’ context and refining our ToC (eg, because the RPPs are actively seeking to have impact beyond their sites) we will add a third soft systems lens.^35^

### Stage 1: development of the initial ToC and hypotheses

For theory-based evaluations, the ToC or programme theory plays a central role in assuring the quality of the evaluation. It guides the measurement of concepts and the investigation of causal relationships between the activities of RPPs, outputs and outcomes. There is guidance for developing and testing ToC/programme theory for contributions analysis and realist evaluation that we will follow.^32 36–41^ The ToC and hypotheses about CMO combinations will be informed by the literature on RPPs in the US education context, similar partnerships between research and practice in ASC and related fields, and insights about how the approach might translate to the English care home context gathered through the codesign work conducted as part of the first stage of the CCP project.

**Figure 1:**
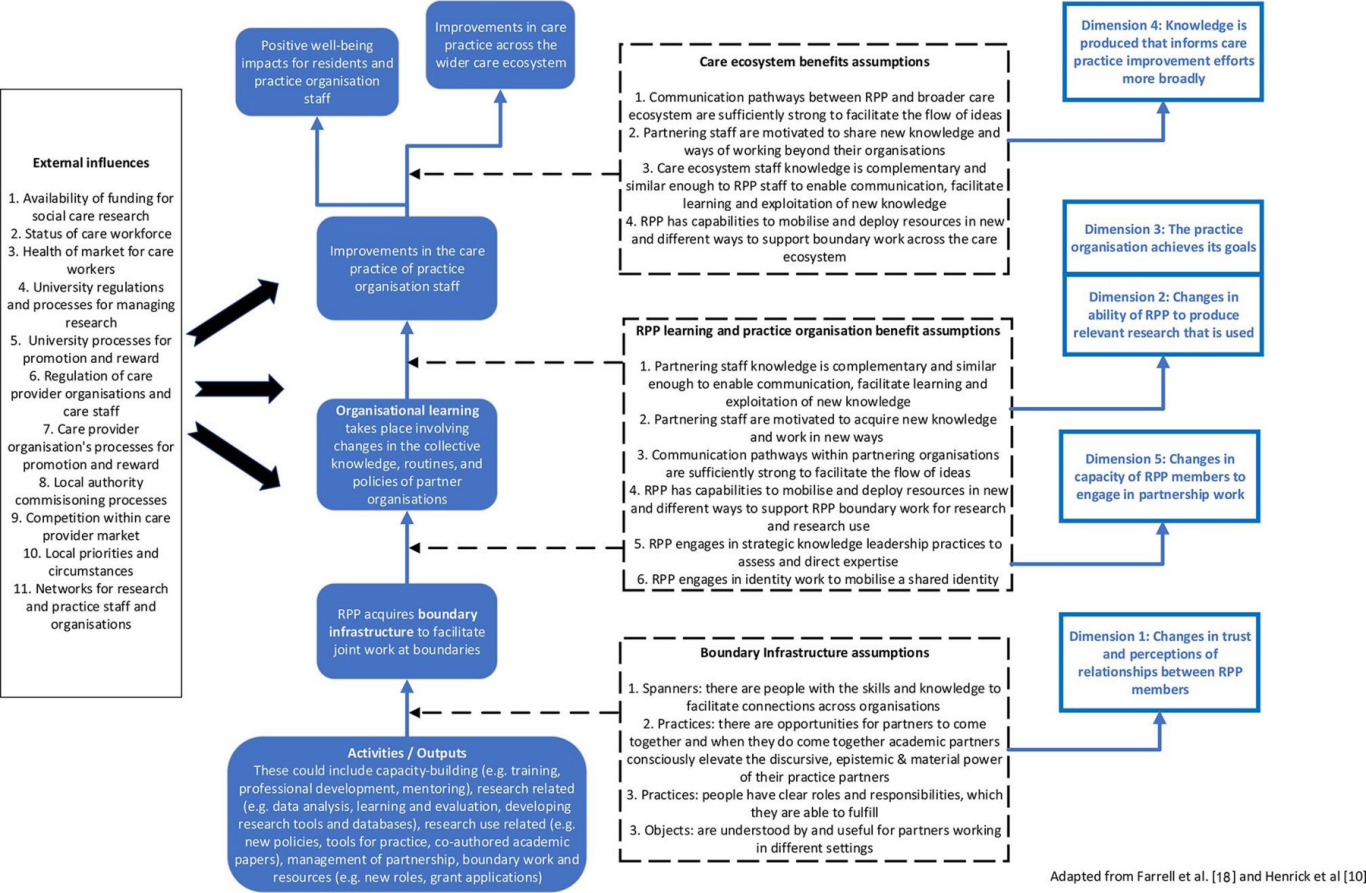
A theory of change for research–practice partnerships (RPP) in adult social care.

Based on an initial review, figure 1 sets out a ToC for RPPs in the care home context. In setting out the theoretical causal chain through which activities/outputs lead to improvements in care practice in the care homes and the wider care ecosystem and the assumptions that need to be met for this chain of events to come about, we draw heavily on Farrell *et al*’s work.^18^ Influencing factors are drawn from our knowledge of the sector and discussions within the codesign workshops. We also illustrate in figure 1 the relationship between the ToC and the five dimensions of effectiveness, which guide measurement.^10^

Figure 1 is a preliminary ToC and a stepping-stone towards developing initial CMO hypotheses. The ToC does not illustrate well the trajectories to economic impact, hypotheses about how the context might trigger certain mechanisms and outcomes, nor our expectation that the journeys for each RPP will resemble a ‘ripple effect’, in that outcomes from earlier activities may form the context for later activities and outcomes.^42^ Ultimately, systems diagrams with feedback loops to identify how inputs, activities and the outputs of those activities affect stocks of trust, and organisational capabilities to produce and use research, may be useful, as might participatory systems and ToC mapping approaches with the sites to identify economic value and understand complex local systems.^42 43^ The use of such approaches, however, will depend on willingness and progress made by the sites.

### Stage 2: conducting the evaluation through testing and refining theory

We will use a mixed-methods design for each case study,^44^ including semistructured interviews, observation of RPP events and meetings, an online survey, activity diary and review of local data and documents. The different methods allow for evaluation of a broader range of outcomes, unintended consequences and provide greater confidence in the measurement of key constructs and the evidencing of claims about the effectiveness of the new CCP partnerships. This approach is common among empirical studies of research use to make sure it is not overestimated,^45^ and has been widely employed in RPP studies.^7 10 46^ Data will be collected from August 2022 to October 2024 in multiple rounds and will be guided by ongoing refinement of the ToC/programme theory.

To ensure we gather a range of perspectives on the development of the CCP partnerships, we will seek insights from members of RPPs (ie, researchers, residents and family members, care home staff and other professionals who participate in the partnership) and wider stakeholders. These are people not directly involved in partnership work but who have a key stake in its success or influence its progress. They could include the university leadership, local authority staff (commissioners, social workers), owners or directors of care home groups, local trade associations, CQC inspectors, etc.

#### Survey

A web-based survey, designed using Qualtrics, will be sent to all RPP members at each partnership, on a roughly 6 monthly basis, starting at baseline and around three to four further times over the project (see online supplemental file 1). As partnerships are small (we expect around 10 people), the survey will take an enumeration sample of partnership members. The research team will send two reminders over a 6-week period to maximise response rates.

10.1136/bmjopen-2022-068651.supp1 Supplementary data

The questionnaire builds on a validated tool to evaluate the progress of US RPPs in the education context against the five outcome dimensions,^47^ but includes adaptations and changes to fit this context and address the research questions. The questionnaire captures trust and perceptions of relationships; whether the partnership has routines for doing and using research (capturing organisational capabilities); participation in partnership activities (capturing boundary working); relevance of research to practice; whether the partnership is achieving its goals; the impact of the partnership on the care home practitioners and their practice, and the wider sector; evidence of investment in the partnership and its members to enable everyone to participate fully.

To capture contextual factors of influence, we also included a question on individual skills and knowledge to participate in the partnership; a set of questions of particular relevance to practitioners on attitudes to research,^48^ and four-item personal research skills and knowledge subscale from the R&D culture index^49^; a set of questions of particular relevance to researchers on personal coproduction skills and knowledge^50^; a set of questions on their employer’s (not the RPP’s) research culture^48^ and culture with respect to coproduction; and a set of questions on identification with and commitment to the RPP that draw on Mael and Ashforth’s^51^ six-item scale of organisational identification and four items from Meyer and Allen’s^52^ affective commitment scale, dropping an item that could not be translated to this context and another that is not considered part of affective commitment.^53^ In the final survey wave, for those questions that ask respondents to judge their skill or experience level, we will consider using retrospective pretests that allow respondents to rate themselves retrospectively from the beginning of the partnership compared with at the time of the final survey. This has been found to remove response shift bias and provide a more valid result than traditional pretest and post-test ratings when respondents are providing self-evaluations of their knowledge.^54^

A survey of stakeholders might be warranted to capture what they view as the significance and value of partnership work. As its value depends on progress of the RPPs, it is not currently planned.

#### Interviews with CCP partnership members, wider stakeholders and CCP team members

For each partnership, we will hold 1-hour long semistructured interviews with CCP partnership members and stakeholders at the start of the partnership process and at three points thereafter. The number of interviews will be determined on a case-by-case basis, based on involvement in activities of the partnership, influence over the operation of the partnership and their ability to inform the research, but for CCP partnership members, it is likely to be around seven to eight interviews at each wave and for stakeholders around three. Initially RPP members and stakeholders will be chosen in consultation with the main site contact, but in order to minimise selection bias and the marginalisation of people, a ‘snowballing’ identification practice will be implemented.

Inevitably, as the partnerships develop the focus of the interviews will shift from capturing the setting up of the partnerships, to doing research as an RPP, and then to using research for organisational learning, wider knowledge exchange and impact beyond the partnership. Topic guides will be informed by the ToC and hypotheses about CMO combinations. Prompts and probes will ensure we explore power dynamics, trust, and the wider organisational and system context characterised by competing interests and values in shaping the trajectory of these partnerships.

We will also conduct 1-hour semistructured interviews with members of the CCP team who are leading the codesign, implementation and user and stakeholder involvement activities shortly after the codesign process has ended, and about three further times over the course of the implementation phase in broad alignment with the timing of support activities. The aim of these interviews is to capture the CCP team members’ experience of delivering support activities to the partnerships and their views on how the partnerships are responding to the support, developing and using the support to shape their partnerships.

All interviews will be audiorecorded and transcribed (see online supplemental file 2 for interview proformas.)

10.1136/bmjopen-2022-068651.supp2 Supplementary data

#### Observation

We will conduct observation of partnership events for each case study to understand how the work of RPPs is being carried out in practice. The observations will focus on interactions, such as how the RPP members and attendees at events work together, make decisions and put their ideas and strategies into practice, and will be informed by an observational framework. Field notes will be written up for each event observed.

#### Activity diaries

To capture the time RPP members spend on different partnership activities, they will complete an activity diary. To facilitate entry in real time, we propose that they use an existing time tracking app (Harvest, http://www.getharvest.com). Data will be visualised on an ongoing basis using the app and downloaded on a monthly basis.

We have included a development and testing phase to explore the best way of reporting activities and the feasibility of using the app at each site. Given the different types of activities that partnerships might use to enact RPP principles, we will use this phase to build up a categorisation for data entry. It will also help us understand the best way to integrate the collection of these data into RPP members’ routines to ensure high-quality data.^13^ We will hold a workshop with each CCP partnership to develop solutions to these and other issues that members may have, including access to smartphones.

We will develop tailored guidance for the CCP partnerships and training that can be rolled out if new members join the partnership during the evaluation.

#### Routine and project-related data and local documents

Routine data, data related to research projects and documents produced by, for or about the partnership will be collected from each partnership on a regular basis. The aim of collecting this information is to provide insight into the plans for and activities of the partnership, research it is producing and using, and the relationship between the partnership and its parent and other organisations.

At this stage, it is difficult to say what the information might look like, as it will be highly dependent on the plans and research agenda for each site. Based on learning from the interviews and observations, we will develop a template of the types of information we will request from the CCP partnerships on a quarterly basis. Examples might include, meeting minutes, data analysis notes, tools developed, grant proposals. This will ensure a degree of consistency in what we request from sites. The template will be reviewed and revised as the partnerships develop.

### Reflecting on evaluation practice

The aim of the evaluation is not to provide a definitive judgement about the effectiveness of each RPP. We recognise that RPPs are on a journey and judgements about their value would be time-bound and unstable.^55^ Instead, we aim to learn more about what can be achieved through the RPP approach as it is introduced in a new context and how the principles can be successfully enacted.

Although we are not providing a definitive judgement about the effectiveness of each new RPP, we will need to reflect critically on our practices and be sensitive to the ways in which they and the evidence we produce might influence the ways in which the CCP partnerships develop.^56^ In this vein, we do not take a formative approach to the evaluation. This is in part to ensure a degree of independence, but more importantly, it ensures that our activity does not prevent the new partnerships from developing their own capacity to monitor and evaluate their work, since this might in the long-run undermine the sustainability of the partnership. We do recognise, however, that our evaluative judgements will be of value to the sites and have planned several feedback sessions. This will need to be situated within processes for learning and action, and will be delivered with the CCP implementation, and sustainability and spread teams. These workshops will enable sites to learn from the evaluation and use the information to improve how their partnerships are working.

### Patient and public involvement

The CCP project includes a public member as part of the Management Team, who has the role of Involvement Lead. The involvement lead contributed to the development of the proposal, methods and will advise the team throughout the various phases of the project.

The study has a lived experience reference group, comprising people with lived experience of receiving and giving care. Their main role is to provide support to the sites to involve the public and people with lived experience of giving and receiving care in their work. They also support the main CCP team, and have provided feedback on the data collection tools to ensure they are accessible. They will continue to advise the project team on the accessibility of data collection tools and outputs related to the project. We thank the advisors for their input to the evaluation.

## Analysis and synthesis of the data

The aim of the analysis is to provide evidence about whether RPPs are a promising approach for driving improvements in practice in the care home context and to understand how, why and in what circumstances RPPs contribute to enhancing research and research use in local care homes and informing wider care home improvement efforts. Since this is a longitudinal evaluation, data will be gathered in waves and analysis will proceed iteratively, using evidence gathered from previous waves to inform subsequent data collection. Following each data collection wave findings will be updated to generate a picture of how the CCP partnerships are developing over time, and the ToC/programme theory refined as we learn more about how and why the CCP partnerships are working and the kinds of impact they are having. At each wave the available data will be analysed in stages.

The first stage is to prepare descriptive profiles for each site. Each dataset will be analysed independently initially. We will use framework analysis,^57^ supported by NVivo software to index the qualitative data (interviews, observation, document analysis) and identify evidence for outcomes, outputs, key constructs (eg, boundary infrastructure), activities or strategies being enacted by the partnership. To inform decisions about whether or not data can be considered as evidence for or against outcomes, outputs and key constructs we will draw on theory and studies of research use.^46 58^ Working within-case study sites, we will then compare across data types to triangulate evidence for each outcome, output and activity in a first stage of synthesis. This will enable us to develop outcome, output and activity profiles for each site, which will be used for the economic analysis.

Subsequently, analysis will focus on the subquestions, working first within case study sites then comparing across case study sites. The outcomes profile will enable us to assess subquestion 1a—the extent to which outcomes have been achieved by each RPP. To address subquestion 1b and determine how significant a contribution the CCP partnership is making to the observed outcomes, we will use contribution analysis. We will follow the analytical steps outlined by Mayne and practical guidance^39–41^ to use the evidence we gather to assemble and assess the contribution stories for how the partnerships have led to research being produced that is used to improve practice within the site and care improvements beyond the site. An important part of this analysis will be to understand the influence of the CCP codesign and implementation support teams. Comparing across case studies to identify whether patterns are consistent or are specific to particular CCP partnership will be important for ToC refinement.

We will complement our use of contribution analysis by drawing on realist methods to explore in more depth how, why and the circumstances in which the CCP partnerships contribute to each outcome (subquestion 1c). The focus will be on developing and refining links between CMOs, following guidance for realist evaluation,^36^ as well as exploring narrower aspects of causality within the broader ToC.^59^ As the analysis progresses, we will explore how later CMOs relate to and might depend on earlier CMOs.^60^ We will also investigate whether these patterns occur regardless of context, or are specific to particular CCP partnerships by comparing across sites. This analysis will provide insight, for example, into whether certain strategies are more suited to particular contexts.

Finally, we will explore whether the way in which the CCP partnerships are operating is consistent with the RPP approach (subquestion d). Additional coding schemes will be developed to capture who is involved in the activities, their context and purpose, the way in which they are being enacted (eg, power differentials are present and not addressed), their consequences and the contextual factors influencing the initiation and progress of the activities/strategies. As coding proceeds, the team will write memos to capture thinking around whether activities/strategies can be considered as faithful to the RPP approach, the applicability of the RPP approach to the social care context and what these new partnerships can tell us about whether the core principles underpinning RPPs need to be adapted.

## Economic evaluation

The economic evaluation will focus on exploring some of the more tangible economic consequences and use knowledge on indicators to model economic consequences for different types of outcomes. The analytical objectives for this stream are to establish the costs and economic consequences of RPPs, which combined will be used to derive an understanding of economic value of the RPP approach. The economic evaluation aligns with the theory-based evaluation and will draw on the data collection and analysis, using in particular the activity, output and outcomes profiles.

Full cost-effectiveness analysis would not be appropriate. Instead, we will use a ‘narrative’ economic analysis to examine both the costs of delivering the RPPs and some of the potential economic consequences. This method, widely used in the social care context draws on simulation modelling and cost–consequence analysis techniques.^61–63^ It provides information on the estimated costs of an initiative and the estimated cost of alternatives, enabling the decision maker to determine whether a course of action is worth investing in given the particular context in which they operate.

There are two parts to the analysis: part 1, assesses the costs of delivering RPPs, and part 2, models the economic consequences of RPPs. The two parts are subsequently synthesised to assess the value for money for each of the RPPs. We will take a health and social care and broader societal value perspective taken in the economic analysis. The latter will consider improvements in (healthcare related or social care related) quality of life, productivity and unpaid care.

### Assessing costs

To cost the RRPs, we will use both bottom-up and top-down approaches.^64^ Unit costs will be attached to each activity in the activity profile, using local sources where possible or—where this is not possible—adapted from national sources to reflect local salaries, overheads and capital costs. Budget information will be used where it is not possible to obtain bottom-up data on activities and for other resource use. Descriptive costs profiles will be developed for each site.

### Modelling economic consequences

Potential economic consequences will be established through the ToC development and refinement process. As a first step, this will therefore include the further development of the outputs and outcomes profiles, to derive economic indicators, and expected trajectories to potential economic impacts. From this, economic vignettes will be drawn for each site.

In a next step, monetary values will be assigned to outputs and outcomes identified in the vignettes as being linked to economic impacts. For some of the economic impacts, it will be possible to attach monetary values either directly, or based on data from published sources (through modelling). An example of a consequence of direct monetary value is the income gained from a joint grant activity. An example of a consequence that would require further modelling to assign a monetary value is the implementation of an evidence-based intervention as part of service and quality improvements known to be cost-effective (such as the implementation of cognitive stimulation therapy for people with dementia^65^). Modelling will use (where available) local data or information from the sites, and published data.

Since some of the economic gains will be realised during the research period while others will take place in the future, the analysis will have different time horizons (eg, short term, medium term and long term) reflecting differences in the certainty of (potential) economic gains. For example, it may be the case that a research project completed during the study period with known economic consequences for the care homes, but in another site a research project may only just have started or may still be at the planning stage, but nevertheless with expected but uncertain future economic consequences.

### Cross-site comparison and synthesising costs and economic consequences

As economic consequences are likely to differ across sites, we need a way of structuring and categorising them to facilitate a narrative comparison between RPPs. Our starting point it to use the ‘Payback Framework’, which has been developed for examining the impact of health research.^66^ It offers a multidimensional categorisation of benefits ranging from more traditional academic benefits of knowledge production to wider benefits to society, but it may need some adaptation to this context.

## Ethics and dissemination

The study has undergone ethics review by the HRA Research Ethics Committee (REC) and has been reviewed in accordance with the London School of Economics (LSE) Research Ethics Policy and Procedure. The study does not pose major ethical issues or chance of harm for participants. Key issues relate to the observational component of the research and working with care home providers and ensuring steps to maintain confidentiality.

### Processes for consent

Although the partnerships are obliged to participate in the evaluation, participation of individuals is voluntary. We will obtain written informed consent from all partnership members and stakeholders for all research activities that they will participate in. Consent will be obtained at the start of the research and again at the start of each research activity, with participants able to withdraw at any time.

Consent for observations of events and other activities related to the partnerships that involve people who are not closely connected to the partnership (and therefore have not previously given consent to be involved in research activities) will be achieved through negotiated and privileged access to the field and implied consent. An information sheet will be sent to participants in advance with the papers for events and a script prepared for the event chair to introduce the researchers. Participants will have an opportunity to raise concerns at the start of any event and refuse permission for observation. Discussion of the CCP evaluation aims and objectives with the partnerships at the outset of their work and through personal conversations with the local evaluators will contribute to raising awareness and enabling implied consent.

### Data management and anonymisation

Data will be stored and managed in accordance with university and national rules and regulations as described in the project data management plan. Steps will be taken to minimise any risk of breaching confidentiality of research or personal data. Any personal information that could identify participants (such as name or job title) will be removed or changed before results are made public. All data collected from the activity diary and the survey will be reported at an appropriate level of aggregation so individuals cannot be identified.

### Dissemination

Outputs will include interim case study reports and a comparative report for each analysis phase. The economic analysis will be conducted towards the end of the evaluation timeframe so will be included in the final report. An economic framework will be produced that can be used by those who want to replicate the analyses of economic value of RPPs.

The final analysis and synthesis will be published as a final report and articles covering the different aspects of the evaluation will be submitted to international journals. The final report will feed into three workshops to be held at the end of the project. These workshops will focus on the sustainability and spread of the RPP approach beyond the CCP sites to the rest of the UK, and sharing leaning from the study with interest groups, thought leaders and senior policy makers in social care from across the UK.

## Supplementary Material

Reviewer comments

Author's manuscript
