# Supplementary material for: Theory-based evaluation of three research–practice partnerships designed to deliver novel, sustainable collaborations between adult social care research and practice in the UK: a research protocol for a ‘layered’ contributions analysis and realist evaluation
Source: BMJ Open. 2022 Nov 25;12(11):e068651. doi: 10.1136/bmjopen-2022-068651 (PMC9703321; doi:10.1136/bmjopen-2022-068651)
Supplement: Supplementary data [file bmjopen-2022-068651supp001.pdf]

Supplementary file 1: Survey for CCP evaluation

This is a survey for CCP partnership members. It will be distributed by email and will be completed online, using Qualtrics software.

This questionnaire is based on a questionnaire that has been originally developed by the National Center for Research in Policy and Practice, Boulder, Colorado, USA. Full reference: National Center for Research in Policy and Practice. Research-Practice Partnerships Outcomes Survey [field test version]. Boulder, CO: 2021.

Due to the differences in context, some of the questions have been adapted to reflect the English social care/care home context. The questionnaire has been shortened to exclude some questions where data will be collected by other data collection methods. For example, we have excluded questions gathering factual information about partnership activities, as these will be collected via the activity diary. We also excluded questions asking about power dynamics between partners, as this will be captured through interviews and observations. Where the National Center for Research in Policy and Practice survey did not cover areas identified as important within our theory of change, we drew on other validated surveys to add questions. The scales used are cited in the main paper with full references, and are:

- ***Questions on practitioners' attitudes to research from a survey by Penuel et al (2016)***
- ***A four-item personal research skills and knowledge sub-scale from the R&D culture index by Watson et al (2005)***
- ***Questions on the employer's (not the RPP's) research culture from a survey by Penuel et al (2016)***
- ***Questions on identification with and commitment to the RPP that draw on Mael and Ashforth's (1992) six-item scale of organisational identification and four items from Meyer and Allen's (1991) affective commitment scale***

A question asking whether the respondent has completed the survey before has been added so that questions that would have the same response at each survey wave will be excluded following the first completion. We have also included q.2 to identify whether respondents identify as being from a research or a practice organisation, which will allow us to route respondents to relevant questions.

The introductory information for the survey has been removed.

CCP partnership member survey, CCP Project, v3.0
10/06/2022

Creating Care Partnerships (CCP) Evaluation Online Survey

Questionnaire

About the partnership and your role in the partnership

A note on how we're using the following terms in this survey:

Partnership: We use the term partnership to refer to the collaboration between research and practice that is part of the Creating Care Partnerships project. We are not referring to any other partnerships that your organisation may be involved in.

Partners: Individuals or groups who are actively involved in the work of the partnership. This might include care home managers, care home staff including nurses, care workers, activity providers, cooks, and other staff, care home residents, family members of care home residents, Local authority commissioners, other local leaders, and researchers.

Non-academic partners: Individuals or groups who are involved in the work of the partnership who are not employed within Higher Education Institutions or other research organisations. This might include care home managers, care home staff including nurses, care workers, activity providers, cooks, and other staff, care home residents, family members of care home residents, Local authority commissioners, other local leaders.

My organisation: This relates the organisation you are employed with.

Stakeholders: Individuals or groups who have a stake or interest in issues relevant to caring within a care home but who are not necessarily actively involved in the partnership.

Research: An activity in which people aim to answer questions by using evidence from observation or experience that has been specifically collected to answer the question. This is different from using data which is already collected for more general purposes by care homes, local authorities or nationally.

Practice: issues of importance to care homes or social care, care delivery, residents/families, and care home staff.

CCP partnership member survey, CCP Project, v3.0
10/06/2022

Q1. Please indicate below if this is your first time completing this survey.

1. Yes
2. No

If Q1=2 then do not ask questions 2-4

Q2. What type of organisation do you work for?

1. Care Home provider
2. Local authority
3. Other social care organisation
4. Higher Education Institution
5. Other research organisation
6. Other (please specify)

Q3. What is the name of the organization or institution that you work for?

[open response]

Q4. How would you identify your primary role in the context of this partnership?

1. Care assistant
2. Senior care assistant
3. Care home deputy / assistant manager
4. Care home manager
5. Care home group leader/manager (central office)
6. Registered nurse
7. Social worker
8. Researcher
9. Research nurse
10. Other, please specify

Q5. Overall, how satisfied are you in your job?

1. Very satisfied
2. Quite satisfied
3. Neither satisfied nor dissatisfied
4. Quite dissatisfied
5. Very dissatisfied

Q6. Do you have a formal leadership role within the partnership?

1. Yes
2. No
3. Not sure

CCP partnership member survey, CCP Project, v3.0
10/06/2022

If Q6 = 2-3 then ask

Q7. Can you describe this role?

Insert free text answer

Building trust and cultivating partnership relationships

If Q1=2 then do not ask

Q8a. Did you know any of the other partnership members before this partnership's work began?

1. Yes, I knew at least one other partnership member.
2. I didn't know any partnership members personally, but another person whom I trusted did.
3. No, I didn't know any partnership members at all.

Display This Question: If Did you know any of the other partnership members before this partnership's work began? = Yes, I knew at least one other partnership member.

If Q1=2 then do not ask

Q8b. In what way did you know other partnership member(s) before this partnership's work began?

Select all that apply.

1. I knew them personally, outside of professional work.
2. I worked with them previously when we were employed within the same organisation
3. I have collaborated with them previously, but not in a formal partnership
4. I worked with them in a formal partnership.

Q9. Indicate how much you agree or disagree with each statement.

	Strongly Disagree	Disagree	Neither agree nor disagree	Agree	Strongly Agree
Our partners follow through when they commit to something.					
Our partners finish tasks when they say they will.					
Our partners' work is reliably of high quality.					
We have supports in place to help people					

follow through with their commitments.					
I can count on my partners to help me outside of our formal commitments.					

Q10. Indicate how much you agree or disagree with each statement.

	Strongly Disagree	Disagree	Neither agree nor disagree	Agree	Strongly Agree
Partners listen to what each person has to say.					
Partners withhold information that is relevant to the partnership.					
Partners feel comfortable discussing an issue when a conflict arises.					
Even when we disagree, I feel that my opinions have been recognized.					
I don't feel comfortable raising concerns I have.					

Q11. Indicate how much you agree or disagree with each statement.

	Strongly Disagree	Disagree	Neither agree nor disagree	Agree	Strongly Agree
I trust my partners are honest with me					
Even in difficult situations, partners can depend on one another.					
I trust my partners will share important information with me					
Partners tend to do what is in their interest, regardless of whether it benefits the partnership.					

CCP partnership member survey, CCP Project, v3.0
10/06/2022

It is difficult to work in our partnership because of existing mistrust.					

Q12. Indicate how much you agree or disagree with each statement.

	Strongly Disagree	Disagree	Neither agree nor disagree	Agree	Strongly Agree
I feel good about working with other people in this partnership.					
All partners have something valuable to contribute.					
Partners have high and reasonable expectations for each other's contributions.					
Partners sometimes have to guess about each other's needs.					
Partners treat each other with care.					
Partners are continually learning new things that are useful in their daily lives.					

Conducting relevant research to inform care home practice

Q13. To the best of your knowledge, how often has your partnership discussed the following in partnership meetings in the past year?

	At every meeting	At most Meetings	Occasionally	Rarely	Not at all
Ideas from research conducted in other contexts					
Data collection or analysis strategies					
Quantitative data representations (e.g., graphs)					

CCP partnership member survey, CCP Project, v3.0
10/06/2022

Qualitative data e.g., interview transcripts)					
Findings and implications for action					
Equalities, diversity, and inclusion considerations to the work					

Q14. In our partnership, I have participated in...

(Mark all that apply.)

1. Deciding what topics and issues to research
2. Developing a grant proposal
3. Developing a literature review
4. Creating a research design
5. Gathering new or existing data
6. Developing resources for storing and accessing data
7. Helping to analyse data
8. None of the above

Q15. In our partnership, I have participated in...

(Mark all that apply.)

1. Organising opportunities for partners to discuss research/inquiry findings
2. Sharing findings
3. Sharing my thoughts on research findings
4. Taking on a specific role to communicate findings across academic and non-academic partners
5. Developing new interventions, strategies, or tools based on our findings
6. None of the above

Q16. In our partnership, I have participated in...

(Mark all that apply.)

1. Co-authoring written outputs from the research
2. Co-designing products developed by the partnership, e.g. innovative practices, guidelines, materials etc
3. None of the above

Q17. Indicate how much you agree or disagree with each statement.

Our partnership's work...

	Strongly Disagree	Disagree	Neither agree nor disagree	Agree	Strongly Agree
Addresses a central concern for care home practice					
Addresses a pressing need of those who have a stake in or are affected by care homes.					
Addresses a gap in the academic literature					

Supporting the non-academic partner organisation in achieving its goals

Q18a. Which best describes the extent to which your partnership has made progress on its goals, aims or objectives?

1. We have not identified goals, **aims or objectives** and do not plan to.
2. We are in the process of identifying goals, **aims or objectives**.
3. We have specified goals, **aims or objectives** to address an issue that is important to non-academic partners
4. We have made progress in working toward our goals **aims or objectives**.
5. We have accomplished some of our goals.
6. We have accomplished and are extending our goals.

Display This Question:

If Which best describes the extent to which your partnership has made progress on its goals? = We have specified to goals to address an issue that is important to non-academic partners.

Or Which best describes the extent to which your partnership has made progress on its goals? = We have made progress in working toward our goals.

Or Which best describes the extent to which your partnership has made progress on its goals? = We have accomplished some of our goals.

Or Which best describes the extent to which your partnership has made progress on its goals? = We have accomplished and are extending our goals.

Q18b Indicate how much you agree or disagree with each statement.

	Strongly Disagree	Disagree	Neither agree nor disagree	Agree	Strongly Agree

We are working toward a common set of goals.					
We have some goals in common and some goals that differ.					
Partners hold substantially different goals.					
Our goals were set by a narrow group, and stakeholders did not have opportunities to contribute.					
In creating or revising our goals, there were multiple opportunities for stakeholders to be involved.					

Q19. Which is most true for your partnership's impact on the care home partner organisation's decisions?

Our partnership...

1. Is too new to have impacted decisions yet.
2. Aims to impact decisions, but we have run into challenges.
3. Is in the process of informing decisions, but no changes have been made yet.
4. Has impacted decisions in the care home partner organisation
5. Does not aim to impact the care home partner organisation's decisions.

Display This Question:

If Which is most true for your partnership's impact on the care home partner organisation's decisio... = Has impacted decisions in the practice/community organization.

Q20. Due to the partnership's influence, the care home partner organisation has...

(Mark all that apply.)

1. Adopted new policies, programs, or practices
2. Participated in designing new policies, programs, or practices
3. Improved existing policies, programs, and practices
4. Allocated more resources to particular policies, programs, or practices
5. Ended support for existing policies, programs, or practices
6. Designed new professional learning opportunities or training

CCP partnership member survey, CCP Project, v3.0
10/06/2022

7. Created a new framework or set of ideas to help think about how to address care home issues
8. Other (please specify)

Q21. Indicate how much you agree or disagree with each statement.

Because of this partnership, non-academic partners...

	Strongly Disagree	Disagree	Neither agree nor disagree	Agree	Strongly Agree
Are more informed by research in their thinking across a variety of decisions.					
More often turn to research to directly inform the decisions they make.					
More often point to research to persuade others.					
More often point to research to justify a decision that has already been made.					
More often use varied forms of research evidence in making decisions.					

Producing knowledge that can inform care home and social care improvement efforts more broadly

Q22. What is most true about your partnership's efforts to share knowledge created through the partnership's work with audiences outside of the partnership?

1. We actively share knowledge in a range of forms and venues.
2. We share knowledge in some ways but would like to do more.
3. We have plans to share knowledge but have not yet done so.
4. It is not a priority to share knowledge created by our partnership.

CCP partnership member survey, CCP Project, v3.0
10/06/2022

Display This Question:

If What is most true about your partnership's efforts to share knowledge created through the partner... = We actively share knowledge in a range of forms and venues.

Or What is most true about your partnership's efforts to share knowledge created through the partner... = We share knowledge in some ways but would like to do more.

Or What is most true about your partnership's efforts to share knowledge created through the partner... = We have plans to share knowledge but have not yet done so.

Q23. Has your partnership identified external audiences for your work?

1. We have identified multiple audiences, including research, care home/social care, and non-academic audiences for our work
2. We identified either research or care home/social care, non-academic audiences outside of our partnership for our work.
3. We have not begun identifying audiences outside of our partnership, but are interested in doing so.

Display This Question:

If What is most true about your partnership's efforts to share knowledge created through the partner... = We actively share knowledge in a range of forms and venues.

And What is most true about your partnership's efforts to share knowledge created through the partner... = We share knowledge in some ways but would like to do more.

And What is most true about your partnership's efforts to share knowledge created through the partner... = We have plans to share knowledge but have not yet done so.

Q24. In which ways does your partnership share knowledge?

	Already do	Plan to do	Have not done and do not plan to do
Online media (e.g., website, blog, webinars, podcasts, newsletters, social media, etc.)			
Research-focused products (e.g., journal articles)			
Practice, care home/social care or policy-focused products (e.g., magazine articles, briefs, reports)			
Presentations at meetings for research audiences			

CCP partnership member survey, CCP Project, v3.0
10/06/2022

Presentations at meetings for practice, care home/social care, or policy audiences			
Storytelling or theatrical representations			
Makes connections with other networks or expands work to other settings			
Acts as a resource for other partnerships			
Has staff with dedicated communications responsibilities			
Applies for further funding to upscale partnership research project			
Other (please specify)			

Q.25. Has your partnership engaged with any of the following groups or networks to share knowledge?

	Already do	Plan to do	Have not done and do not plan to do
Applied Research Collaborations (ARCs)			
NIHR Enabling Research in Care Homes (ENRICH)			
Local and regional provider associations			
Association of Directors of Adult Social Services (ADASS)			
Professional bodies (e.g. British Association of Social Workers, Royal College of Nursing)			
Research in Practice for Adults (Ripfa)			
Social Care Institute for Excellence (SCIE)			
Other research networks (please specify)			
Local and regional provider associations			
Association of Directors of Adult Social Services (ADASS)			

CCP partnership member survey, CCP Project, v3.0
10/06/2022

Building the capacity of participating researchers, practitioners, care homes, and research organisations to engage in partnership work

Q26. Our partnership has created opportunities for partners to learn more about the following aspects of the research process:

(Mark all that apply.)

1. Asking research/inquiry questions that matter
2. Collecting data using different methods
3. Analysing data and interpreting findings
4. Presenting findings for different audiences
5. Knowing how to link research results to key issues facing decision-makers
6. None of the above

Q27. Our partnership has created opportunities for partners to learn more about the following aspects of co-design between academic and non-academic partners:

(Mark all that apply.)

1. Designing, testing, and/or adapting delivery processes or materials
2. Designing, testing, and/or adapting professional development within care homes
3. None of the above

Q28. Our partnership has created opportunities for partners to learn more about the following aspects of the practice-based issue and local context:

(Mark all that apply.)

1. A broader system perspective (i.e., the range of individuals, organisations, and networks that influence the issue at hand)
2. How decision-making unfolds in care homes and/or local authorities
3. The content of the issue at hand
4. The historical, political, or other equity dimensions of the issue at hand
5. Resources in local communities or organizations
6. None of the above

Q29. As a result of working together, participating organisations have...

(Mark all that apply.)

1. Created new positions (e.g., partnership intermediary, broker, or project manager)
2. Revised job descriptions
3. Submitted grant proposals to support the work
4. Dedicated financial resources to support the partnership
5. Dedicated in-kind resources to support the partnership (e.g., staff time, office space)
6. Changed formal policies (e.g., guidelines for promotion)

CCP partnership member survey, CCP Project, v3.0
10/06/2022

7. Altered or developed new evidence based interventions
8. Adopted routines modelled in the partnership's work
9. Produced outputs that are of value the wider research and practice community
10. None of the above

Q30. Indicate how much you agree or disagree with each statement.

In terms of our research/inquiry activities, all partners...

	Strongly Disagree	Disagree	Neither agree nor disagree	Agree	Strongly Agree
Are committed to doing their part.					
Have the time and resources to do their part.					
Have the knowledge and skills needed to do their part.					
Think that the partnership is worthwhile					
All partners are equally involved					

Q31. Does your partnership have a sense of community among its members?

1. We have not yet considered how this partnership could build a sense of community among its members.
2. We see the benefit of building a sense of community among members but are unsure of how to proceed.
3. We are in the process of building a sense of community among members.
4. Our partnership activities have built a sense of community among members.

Q32. What best describes partners' attention to the overall progress of your partnership?

1. We don't pay attention to the progress made by the partnership.
2. We keep our partnership's progress in mind, but we rarely discuss it.
3. We occasionally discuss the overall progress of our partnership and make adjustments if needed.
4. We regularly discuss the progress of our partnership and make adjustments.

Q33. Indicate how much you agree or disagree with each statement.

	Strongly Disagree	Disagree	Neither agree nor disagree	Agree	Strongly Agree
When someone criticizes the partnership, it feels like a personal insult					
I am very interested in what others think about the partnership					
When I talk about this partnership, I usually say ‘we’ rather than ‘they’					
This partnership's successes are my successes					
When someone praises this partnership, it feels like a personal compliment.					
If a story in the media criticized the partnership, I would feel embarrassed					

Q34. Indicate how much you agree or disagree with each statement.

	Strongly Disagree	Disagree	Neither agree nor disagree	Agree	Strongly Agree
I do not feel a strong sense of belonging to the partnership					
I do not feel emotionally attached to this partnership					
I do not feel like part of the family at my partnership					
This partnership has a great deal of personal meaning for me					
I would like this partnership to have more personal meaning for me.					

CCP partnership member survey, CCP Project, v3.0
10/06/2022

Q35. Indicate how much you agree or disagree with each statement.

In terms of our research/inquiry activities, I…

	Strongly Disagree	Disagree	Neither agree nor disagree	Agree	Strongly Agree
I am committed to doing my part.					
I have the time and resources to do my part.					
I have the knowledge and skills needed to do my part.					
I feel supported by my organisation to do my part					

If Q2 = 1-3, 6 then ask.

Q36. Indicate how much you agree or disagree with each statement

	Strongly Disagree	Disagree	Neither agree nor disagree	Agree	Strongly Agree
I understand research terminology					
I feel confident about using research in my practice					
I know how practice is influenced by research					
I have the skills to use the library and learning facilities					

If Q2 = 1-3, 6 then ask.

Q37. I feel confident that I have the knowledge and skills to:

1. Find research to inform policy, care delivery change, practices
2. Evaluate the quality of research
3. Interpret the results of research
4. Apply research to policies, programmes, or practice development
5. Design evaluations of policies, programmes, or practices
6. Commission research to support policies, programmes, or practice development

If Q2 = 4-5 then ask

Q38. Indicate how good you are at the following:

	Excellent	Good	Average	Poor	Very Poor
Acknowledging expertise of non-academic partners					
Being able to work with individuals in non-academic partner organisations					
Building relationships with non-academic partners					
Building rapport through regular contact with non-academic partners					
Being collaborative, not being telling nor controlling					
Engaging with non-academic partners without superior attitude					
Communicating research in plain language					
Explaining complex ideas in a way that non-academic partners can understand					
Understanding the perspectives of non-academic partner's perspectives and showing empathy					

If Q2 = 4-5 then ask

Q39. Indicate how often the following happen:

	Never	Sometimes	Frequently,	All of the time
In my organisation, practitioner's views are seen as a useful source of information for making decisions about what research should be done				

CCP partnership member survey, CCP Project, v3.0
10/06/2022

In my organisation, there is encouragement to work closely with social care practitioners throughout the research process				
In my organisation, it is expected that you will work with practitioners to conduct research that will improve care homes				

If Q2 = 1-3, 6 then ask

Q40. Indicate how often the following happen:

	Never	Sometimes	Frequently,	All of the time
In my organisation, research is seen as a useful source of information				
In my organisation, there is encouragement to use research as part of our ongoing work				
In my organisation, studies are conducted on changes to care delivery/new processes/policies that are implemented to see how they work				
In my organisation, it is expected that if you make a claim in a meeting, you will be able to cite research evidence to back it up				
In my organisation, interaction or collaboration with researchers or research organisations is encouraged				

If Q2 = 1-3, 6 then ask

CCP partnership member survey, CCP Project, v3.0
10/06/2022

Q41. Indicate how much you agree with the following statements:

	Strongly Disagree	Disagree	Neither agree nor disagree	Agree	Strongly Agree
Research helps identify problems facing care homes					
There is a disconnect between the research world and the care home world					
Research addresses questions that help us make better decisions about social care					
When confronted with a new problem or decision, it is valuable to speak with social care researchers					
Social care research is too narrow to be useful to social care leaders or managers					
Social care researchers work in an ivory tower and are isolated from practice					
By the time research findings are published they are no longer useful to me					
Research can address practical problems facing care homes					
Researchers provide a valuable service to social care leaders, managers and workers					
Social care researchers are unbiased					

About You

The remaining questions will allow us to better understand your background. You do not have to answer them if you do not want to.

If Q1=2 then do not ask

Q42. I am:

1. Male
2. Female
3. Non-binary
4. Prefer not to say

CCP partnership member survey, CCP Project, v3.0
10/06/2022

Q43. Which of the following best describes your ethnic group?

1. White (e.g. English, Irish, Scottish)
2. Mixed/multiple ethic group (White and Black Caribbean, White and Black African, White and Asian)
3. Asian/Asian British (e.g. Indian, Pakistani, Bangladeshi, Chinese)
4. Black/African/Caribbean
5. Other (please specify)

Q44. How old are you?

1. 18 years old or younger
2. 19-25 years old
3. 26-30 years old
4. 31-35 years old
5. 36-40 years old
6. 41-45 years old
7. 46-50 years old
8. 51-55 years old
9. 56-60 years old
10. 61 years old or older

Q.45. What is your highest level qualification?

1. None
2. NVQ Level 1
3. NVQ Level 2
4. NVQ Level 3
5. NVQ Level 4
6. NVQ Level 5
7. GCSE (s) (or equivalent)
8. AS level(s) (or equivalent)
9. Undergraduate degree
10. Masters degree
11. Doctorate
