# Supplementary material for: Theory-based evaluation of three research–practice partnerships designed to deliver novel, sustainable collaborations between adult social care research and practice in the UK: a research protocol for a ‘layered’ contributions analysis and realist evaluation
Source: BMJ Open. 2022 Nov 25;12(11):e068651. doi: 10.1136/bmjopen-2022-068651 (PMC9703321; doi:10.1136/bmjopen-2022-068651)
Supplement: Supplementary data [file bmjopen-2022-068651supp002.pdf]

Supplementary file 2: Interview proformas for CCP evaluation

RPP member interview proforma

This proforma will be used for RPP member interviewees, which includes those involved in the design and implementation of one of the three RPP case study sites. These could include care home managers, university researchers, care staff, nurses, members of staff within the local authority or charitable organisations involved with the RPP.

The questions are largely framed as they will be used for the initial interviews, but these will change for later interviews in the way described in the text boxes at the start of each section. We refer to this interview schedule as a proforma to reflect the fact that the content covers the breadth of issues we will want to investigate but questions will not be relevant for everyone at each time point.

At present none of the partnerships have representatives who are care home residents or family members. If membership changes the proforma will be adapted for interviewing these members.

A. Introductory information

My name is XXXXXX. Thank you for agreeing to take part in the Creating Care Partnerships (CCP) study.

[name of partnership] has been selected as one of the case studies for the CCP study. We are interviewing members of [name of partnership] to get a better understanding of your experiences of implementing the RPP approach in care homes in England; how, why and in what circumstances the RPP approach contributes to enhancing the quality of research and research use in care homes in England; and the costs and benefits of delivering RPPs.

The interview should last about an hour. During the interview I will ask you about how your partnership is developing its capacity for doing and using research to benefit practice in care homes, the types of activities you do as a partnership and the consequences of those activities. I will also ask you about importance of external events and local conditions in influencing how your partnership works and its ability to achieve its goals.

I am sure there will be some areas you have more knowledge about. Where there are gaps in your knowledge it would be helpful if you could identify people or key documents that may help us to better understand that part of partnership working.

Malley J, et al. BMJ Open 2022; 12:e068651. doi: 10.1136/bmjopen-2022-068651

Informed Consent

1. Can I just check that you received the study information sheet and that you have had a chance to read it?

   No - [Review the study information sheet in detail]
   Yes - *Good* [Review the study information sheet briefly]

2. Do you have any questions about the wider study or about the interview we will be conducting today?

3. If you are happy to please can you sign the consent form that I sent you and email it back to me for our records. The consent form is a standard form used in social research and is used to ensure that:

- you understand the aims of the study
- you understand what your participation in the study will involve
- you are happy for the discussion to be audio recorded
- you know that you can change your mind about taking part in the study at any time – you can request for the discussion to stop at any time and if at any point you want to withdraw from the study you can request this by using the contact information provided to you today
- you know that your views will be kept confidential and your name will never be used in anything that is written about the study
- you consent to take part.

[Note: if the participant is not able to sign and return the consent form then consent can be recorded orally.]

[Start recording] Software will request participant to give permission to start recording. If face-to-face then request permission to start recording

B. Introductory questions

1. Can you tell me about your current professional role at [name of organisation] and how long you have been in post?

2. What's a typical day for you?

C. Contextual information about the partnering organisations

The aim of this section is to gain some contextual information about [name of partnership] members and the organisation they are employed by. We also want to

Malley J, *et al. BMJ Open* 2022; 12:e068651. doi: 10.1136/bmjopen-2022-068651

explore the personal and organisational motivations for participating in [name of partnership], and understand the background to the relationships in the partnership.

i) Information about the organisation

HEI interviewees

1. Can you me a bit about your department's research interests and ongoing projects? What other organisations does it normally collaborate with?

2. How does your university relate to other organisations/universities in the region? Does it have a strategy with respect to how it works with organisations in its region? Is the university involved in any major regional cross-sector partnerships, e.g. ARCs?

Care Home/local authority/third sector interviewees

1. How would you describe your organisation? What organisations does it normally collaborate with?
   Probe around:
- Organisational type
- The area the organisation serves
- Population it serves/demographic

ii) Reasons for participation in the partnership

1. Can you tell me why your organisation wanted to participate in the [name of partnership]?

2. Can you describe how the partners came together to start the [name of partnership] and respond to the EOI?
   Probe around understanding the initial power dynamics:
- Whether one person or partner took the lead and brought all the partners together or whether partners / people were suggested by different members
- Whether they came together at all, had meetings over the phone, who was involved in the decisions and writing the EOI.
- Whether they discussed resources and how those would be held and parcelled out.
- Whether they discussed a structure for the management and organisation of the partnership, what roles people would have
- Whether they discussed what they wanted to achieve and how they would achieve it
- Whether they discussed ways of working

Malley J, et al. BMJ Open 2022; 12:e068651. doi: 10.1136/bmjopen-2022-068651

D. Perspectives of the partnership member on their role and the partnership

The aim of this section is to explore the personal motivations for participating in [name of partnership], understand the relationships in the partnership and the member's role and capacity to fulfil that role in the partnership.

i) Reasons for participation in the partnership

1. Can you tell me about why you personally wanted to participate in the [name of partnership]?

ii) Experience of research-practice partnership working and relationships between members (previous and existing)

The aim of this section is to collect data on the their views about the potential for research to inform and improve practice, the relationships between partnership members and how these evolve over the course of the partnership. In early interviews we will ask about the formation of relationships prior to the official start of the partnership, but in later interviews we will look at how perceptions of partners are changing.

The issues to dig into are the shifting power dynamics as the partnership progresses, in what respects different partners hold power, and how has it been relinquished, contested, and held onto. We also want to explore the cycles of relationship building, maintenance, breakdown and repair as the partnership progresses and how these feed into future partnership work.

3. Before you started/joined this partnership how familiar were you were working in partnerships with other organisations to produce research for practice improvement? What view did you have about the potential for research to inform improvements in practice? Do you think your previous experience has had an influence on how you have approached this partnership?
Note: for academic partners we are interested in understanding about their experience of previously working with partners from practice around research
Probe around:

- What your previous experiences were like and what you learnt from those previous experiences, focus on experiences or research / experience of working with practitioners to co-produce research

- If no direct experiences, then explore whether they learnt from the experience of others and who they connected with / what they may have read
- How the previous experience/experiences of others influences how you approached this partnership
- Whether working in the partnership has changed their mindset, whether they see a greater / different potential for using research to inform practice

4. Before you started/joined this partnership how familiar were you with your partner organisations in [name of partnership] or members of those organisations? (academic researchers/care homes/social care) Do you think this has had an influence on how your partnership is developing?

Probe around:

- Your initial perceptions of your partners? What influenced these perceptions?
- Whether the previous relationships were personal or in the context of work, and what the previous work was
- Why they decided to work with the organisation

5. Have relationships been something you have discussed as a partnership, and if so why? Have you felt that it would be helpful to get to know members of your partnership better? Why did you think this would be helpful?

6. Have you decided to do anything as a partnership to get to know each other better or change the way you relate to each other? What have you done and how has this affected the partnership?

Probe around:

- Ways to involve people in the work
- Ways to ensure people have more power and voice in processes and decision-making forums
- Communication pathways to facilitate involvement

7. Have there been any difficult moments within the partnership, where people have disagreed over the direction of the partnership? How have these moments of conflict been managed and resolved?

Probe around:

- How did you felt during these times
- Strategies developed to deal with conflict
- Whether the types of conflicts and methods of resolution change over time
- Role of power in this process (dominance, resistance, power plays)

iii) <u>What the member brings to the partnership and their role in it</u>

Malley J, et al. BMJ Open 2022; 12:e068651. doi: 10.1136/bmjopen-2022-068651

The aim of this section is to explore the way in which individual's capacity to conduct partnership work has built up over time.

In the early stages we will concentrate more on the pre-existing skills, capacities and knowledge of partners before moving on to look at the types of individual dispositions, skills and capacities that were needed to engage in partnership work and whether their previous experiences enabled or inhibited this. In the later stages we will concentrate on how skills and capabilities for conducting research and joint working developed over time and the influence of the partnership members and infrastructure, wider networks and resources.

1. What skills and knowledge do you think you bring to the [name of partnership] and why do you think they will be/are helpful?
   Probe around:
- Research skills/experience
- Knowledge of care homes, service delivery, patient/resident population, etc.
- Partnership working experience

2. Can you describe the role you occupy in the [name of partnership]? What role do you expect to occupy?
   Probe around:
- Formal roles and informal roles – what responsibilities they have or have taken on
- Elements of your usual professional role that are most useful to this role
- New aspects/perspectives you needed to take on to engage in your partnership role
- Attitudes/dispositions/values/skills you think make a good partnership worker
- Experience of conflict or difficulties when taking on this new role and how you managed those

3. Can you describe how your role in the partnership has developed over time? Why did/didn't it develop? Would you like your role to develop further?
   Probe around:
- How skills and knowledge have developed over time in relation to the roles played
- Whether the person had any explicit training to support their role development/ mentoring
- How have previous experiences affected ability to do role, the skills/capacities needed
- Skills and capabilities you would still like to develop

4. What have you learnt about how to perform your role in the [name of partnership], the kinds of skills, knowledge and attitude that is needed? What has helped or hindered you?

Probe around:

- Particular knowledge that helps, e.g. learning about each other's org culture
- Particular skills that help or know-how, e.g. experience merging or balancing these perspectives and skills
- Relationships and developing a common language, purpose etc
- Other people being key, e.g. new roles crossing over org boundaries
- Infrastructure being key, e.g. meetings/forums for collaborating
- Support being key, e.g. training, mentoring, activities of CCP team

E. Developing and delivering the partnership: strategic and operational aspects

The aim of this section is to explore the ways in which the partnership is developing. This includes the initial setup, building trust and relationships, and individual level and partnership capabilities.

i) Understanding of the RPP approach and the current strategy for implementing it

The purpose of this section is to capture how the RPP approach is understood, and the goals and priorities for partnerships. It will be important to understand how the work programme is negotiated and agreed and changes as the partnerships develops.

Questions around understanding of the RPP approach and goals are for everyone but the strategy question is just for leadership / those involved in the decision-making

1. What were your first impressions of the RPP approach? Can you describe the RPP approach in your own words?
   Probe around:

- Main aims and objectives
- How similar the RPP idea is to their initial thinking about what the partnership might look like
- What do they think is most important to get right?
- What has influenced their understanding, e.g. previous experience, reading they have done, activities and literature from co-design team, implementation team
- Whether they have learnt anything about this type of working, as they have started on the journey

2. Can you tell me what the goals are for the partnership from your perspective?
   Probe around:
   - How this has changed over time
   - Whether these goals are reflected in a strategy document or have emerged from strategic discussions
   - If there is a strategy document then ask for it if not already seen
   - What the reasons were for choosing these goals, e.g. unmet need, influence of policy / economic context, influence of key stakeholders etc
   - Whose interests were reflected in the goals, how they were negotiated

3. Do you have a main focus of work for [name of partnership] for the short term (e.g. next 6 months or so)? Can you talk me through the process through which your partnership decided on this focus/foci for the work?
   Probe around
   - Who was involved in the decision making?
   - Why this focus was chosen over others, e.g. gap/problem identified, other aspects contingent on this, quick win, advocated by a particular partner, met the needs of a particular stakeholder, influence of university or senior management at provider, or other external players?
   - Whether there were any differences in opinion and what they were about, how they were resolved
   - If there is more than one focus then explore how this is managed?
   - Try to get them to focus on aspects other than the research agenda, e.g. communications about partnership or more generally, capacity-building of staff, building relationships and trust, participation/addressing inequalities of power, bringing in missing voices

ii) Understanding how the partnership's plans will be delivered: structure, organisation and resources within the partnership

The purpose of this section is to capture how the RPP approach is being delivered, whether there is a plan and overarching framework/structure for delivery, whether people have specific roles, what activities are being carried out, by whom and with what resources. It will be important to understand how the partners contribute to explore the degree of participation and power relationships. We will also want to track how the goals and priorities for partnerships change as the partnership develops.<br><br>This does not need to be asked of everyone; mainly for leaders

1. Can you describe the plan for how you will deliver the partnership's work programme?

Malley J, et al. BMJ Open 2022; 12:e068651. doi: 10.1136/bmjopen-2022-068651

Probe around:
- Whether the plan is written down (ask for it to be shared)
- Whether the plan is long-term or short-term (e.g. next six months)
- What the content of the plan is: infrastructure building, capacity-building/career development, research agenda
- How the work is organised, e.g. workstreams or cycles for core partnership activities, research projects

2. Can you describe how you are managing the delivery of the work programme for the partnership?
   Probe around:
   - Governance structure, i.e. accountabilities, leadership, critical friends/oversight
   - Key functions and structures for delivery, e.g. communications, administration, finances
   - Leadership of the work
   - Development of new roles, allocation of roles and responsibilities for the different aspects of the work / is it more informal e.g. people volunteer
   - What is supporting people to work together / deliver the work programme, e.g. regular meetings,

3. Can you describe how you are resourcing the work programme for the partnership?
   Probe around:
   - Explore people – who is involved and which organisation they come from
   - Explore finance and where the money is coming from (e.g. CCP grant, partners own budgets, other sources)
   - Any other resources that need to be brought in, e.g. software/technical expertise, website etc

iii) Understanding the capacity of the partnership to deliver the work programme and intentions to build capacity

The aim of this section is to explore the way in which organisational capacity to conduct partnership work has built up over time.

The issues and themes to dig into are around whether the partnership has the right membership composition in term of skills and knowledge, whether the partnership feels cohesive and members identity with it are the individual level capabilities needed, the types of capabilities needed by different partners and how these are balanced. We are also interested in the relationship between individual level capabilities and the building of partnership level capabilities that can lead to a functioning and sustainable entity.

1. Thinking about the ability of the [name of partnership] to deliver its goals, what would you say are its current strengths and weaknesses?
   Probe around:

- Composition of the partnership? Knowledge, skills or roles missing?
- Sense of partnership being cohesive? Identity and shared language/values/mission etc
- Other resources for the partnership, e.g. sufficient time to dedicate, money, data availability/accessibility
- Strength of organisational capabilities/routines/infrastructure to support partnership activities rather than individuals, e.g. procedures and processes embedded in routines, robust to staff leaving
- Pacing – too fast or too slow

2. Are there plans to address the weaknesses? What do you think could be done to strengthen the partnership?

Probe:

- around whether they have formally assessed strengths and weaknesses
- written plan

3. Can you describe any factors that enable/inhibit [name of partnership] from building up its ability or capacity to deliver its goals?
   Probe around:

- Wider objectives, strategies and priorities within social care or HEI system
- Existing infrastructure, i.e. ENRICH, ARCs
- Organisational types, i.e. private vs local authority care homes
- Professional level/individual level factors
- Resources
- Communication and understanding

4. Do you have a sense of how your organisation and other organisations perceive the [name of partnership]? What do you think their perception is of the partnership and what is your opinion based on?
   Probe around:
   - Interest from other organisations
   - Recognition in local news, organisation meetings/news

F. Doing and using research for real-world change

This section explores the way in which research is being done and used by the partnership and the impact the partnership's research is having on practice more broadly. Key aspects to explore are power dynamics, the degree of participation in all aspects of the research by partnership members and how these activities are being supported by the partnership and routinised.

1. Can you describe the process through which you developed your research agenda? To what extent is there an established process for deciding the research agenda?

Probe around:

- What drove the decisions/source of ideas: problems of practice, other internal organisational factors, external factors, previous research done
- process: generating ideas, negotiating around ideas, developing questions, planning the research, including methods and timescales, resourcing the research
- routinisation: What forums did this take place? Are there standard processes?
- Explore role of power, interests balanced, how and to what extent people were involved in the process

2. Can you describe your research agenda? Are you satisfied with the agenda? What would you have preferred to be different? Do you think others feel the same way?

Probe around:

- The process for developing the agenda
- Whether there are specific projects, and the content of projects,
- The focus and nature of the research
- The quality of the research plan
- Considerations around time
- Involvement of different people in the planned research

3. I'd like to understand the research process in more depth, so can we choose two research projects and you can describe to me how these projects are progressing?
   NOTE: Choose projects that are different either because they vary in size and scope, involve different people or different methods, have been more or less successful. Talk through each project separately covering the questions below to move from developing and doing the research, to interpreting and using the research.

4. First, can you describe to me how the research is being done?
   Probe around:

- the type of research – methods and designs

- who is involved, to what extent, and how has the research been managed to get greater involvement
- Power dynamics: people involved, degree of participation in the process, who is leading, and attempts to change dynamics

5. Have you had to make any compromises when doing the research? What have these been and why did you make them? Who was involved in the decision?
   Probe around:
   - Types of compromise and how this was negotiated/decided: exclusion of people, types of methods, design and research quality
   - Internal drivers, e.g. time and resources; pacing issues related to skills, experience and expertise of partnership members; availability of data
   - External drivers, e.g. needs of management, HEI, policy environment, local concerns,

6. What was the output of the research? How have the findings been summarised and communicated?
   Probe around:
   - Whether outputs are more academic (e.g. reports and articles) or more practice oriented (guidelines, etc), or innovations
   - Methods of communication that crossed different org boundaries
   - The role of key individuals in this process and the role of leaders
   - The role of existing organisational communication pathways
   - Role of power- who controlled the communication process

7. What has the reaction of the partnership been to this research? Have there been discussions about it?

   Probe around:

   - How has the partnership made sense of the findings: formal and informal activities
   - What the focus of sensemaking was e.g. language, purpose, action, link to partnership goals
   - Who was involved in discussions, how were inputs from everyone managed and facilitated
   - Routinisation of sensemaking and research use: meetings, processes for considering research

8. Have there been any actions as a consequence of the research? Can you explain the ways in which you personally/[name of partnership]/[name of organisation] were influenced by the research findings?
   Probe around:
- In what forums/meetings/situations are decisions about changes to services/care/strategies made within care homes
- Changed priorities, agendas and solutions
- Co-design of materials/tools/service innovation, new processes/routines
- Explicitly making a decision based on evidence
- Justified previous decisions/whose interests did the use of research serve

9. Has the research been recognised by anyone who isn't part of the partnership or by any organisation that isn't part of the partnership? Have you tried to interest other people and organisations beyond the partnership in your work?
   Probe around:
   - Other academic institutions, providers, local authorities, NHS, funders, knowledge intermediaries
   - How other found out about it: channels of communication, key people, networks, deliberate strategies for spread
   - Strategies used: upscaling research projects, communication channels and materials, individuals/roles

10. Have you been satisfied with how this research project has gone/is going? What would you have preferred to be different? Do you think others feel the same way?

Probe around:

- The process for doing the research
- The quality of the research
- Considerations around time and resources
- Involvement of different people in the planned research
- Process for sensemaking
- Degree to which research was used, made a difference

11. Can you talk about what types of things made the research project difficult to deliver or helped the research to succeed in its aims?
    Probe around:
- Research approach/design used
- Time and resource to use research
- Organisational culture/pre-existing knowledge
- Leadership and communication pathways internal to care home

Malley J, *et al. BMJ Open* 2022; 12:e068651. doi: 10.1136/bmjopen-2022-068651

- [name of partnership] composition
- Political factors/local authority policy
- Wider HEI/care home/social care context

12. What, if anything, do you think the partnership has learnt from this research project about doing research that is relevant for practice and leads to real world change? How do you think that learning will be used to inform future projects?
    Probe around:
    - What types of things have been learnt, e.g. strategies for involvement, elevating power, ways of communicating, etc
    - What processes are in place to capture learning and ensure it informs future practice
    - Have processes changed as a result, have practices changed as a result

G. Monitoring, evaluation and learning

1. Have you thought about what success would look like for the partnership? Can you describe what success would look like, at one year, two years etc?

2. Do you have any processes in place for monitoring and evaluating what you are doing as a partnership? How are you using that data?
   Probe around:
   - What evidence / data is collected, by whom, e.g. just about research projects or also reflections on how they are working, what is working well / less well
   - Where evidence / data is from, e.g. CCP evaluation team, implementation team, their own data/ tools
   - How is evidence collected: formally through monitoring tools, evaluation, end of project reflection and learning
   - Used to celebrate success, demonstrate success to funders/partner organisations, learning and improvement

3. What do you think are the most important things you have learnt as a partnership? Can you give any examples of how the partnership has changed what it is doing in response to learning?
   Probe around:
   - Learning directly from outputs, e.g. informed practice
   - Changed ways of working to address problems, limitations, improve processes
   - Changed views about who should be involved and to what extent, at what point

Malley J, *et al. BMJ Open* 2022; 12:e068651. doi: 10.1136/bmjopen-2022-068651

- Changed perspective about potential for the partnership, e.g. changed view about the potential for research to inform practice, scope to have impact on partners and wider system

H. Successes and failures, barriers and facilitators

5. What would you say are your partnership's main achievements so far? What have you learnt from this and how will you take this forward into future partnership activities?

6. Is there anything that has gone less well for your partnership? What have you learnt from this and how will you take this forward into future partnership activities?

7. Have there been any challenges or has anything prevented your partnership making the progress you would have liked? How have these impacted on your partnership's progress?
   Probe around:

- Leadership
- Communication
- trust
- Resources
- Internal politics of partnership or organisation
- External factors, e.g. HEI, organisation, wider political / economic context, structures and wider priorities, CCP funding

8. Have you found anything, anyone or any organisation particularly helpful or supportive? How have these had a positive impact on your partnership's progress?
   Probe around:
   - CCP support offered from co-design or implementation
   - Support from partner organisations, e.g. contribution of resources, changing practices, encouragement
   - Support from other organisations, e.g. regional organisations
   - Peer network for partnership projects
   - Products / previous activities of your partnership

9. Can you describe the interactions you have had the with the co-design and the implementation team? What impact has this had on the partnership?
   Probe around:
   - Whether they made a request or were approached

- Types of help you received
- Types of questions you had/frequency
- Other support that would be useful

I. Concluding questions

1. Is there anything that we haven't covered that you would like to mention?

2. Are there any people or organisations that you work with that you think we should speak to as part of our research?

3. Are there any documents that you think we should look at as part of our research?

Malley J, *et al. BMJ Open* 2022; 12:e068651. doi: 10.1136/bmjopen-2022-068651

Stakeholder interview proforma

This proforma will be used for stakeholders connected to the three case study partnership sites. Stakeholders include anyone who has had influence over strategic direction and operations of one the partnership or is part of an organisation that has interest and could be directly influenced by partnership work. This could include university leadership, local authority leaders or commissioners, social workers, directors of care homes, local trade associations, CQC inspectors, local Applied Research Collaborations (ARCs) or other research collaborations, Enabling Research in Care Homes (ENRICH) members, or Clinical Research Networks. We need to adapt the proforma based on who we are speaking to, as not all of these questions will be relevant to all interviewees. As issues emerge, we may need to add questions in to ensure all relevant issues are covered.

The aim of the stakeholder interviews is to explore the way in which those external to the partnership but with a vested interest interact, use, and value the RPP.
In the early-stage interviews, we will focus on gaining insight into the types of organisations and key people who have an interest or strategic oversight of the operations of the partnership and contextual factors relating to their organisation and networks. Early interviews will also seek to gain insight on the level awareness and communication they have of partnership work.

In later interviews, we will look to explore more directly how stakeholders are making sense of partnership goals and research outputs, whether research is useful and useable, and whether the partnership is valuable from an external perspective.

J. Introductory information

My name is XXXXXX. Thank you for agreeing to take part in the Creating Care Partnerships (CCP) study.

We are interviewing stakeholders from the [name of partnership] lead to get a better understanding of your experiences of working with [name of partnership]. The interview should last an hour. During the interview, I will ask you about your level of involvement with the partnership, how you use and value the work produced by the partnership, and external events and local conditions influencing how the partnership works and its ability to achieve its goals.

I am sure there will be some areas you have more knowledge about. Where there are gaps in your knowledge it would be helpful if you could identify people or key documents that may help us to better understand the way your organisation works with the [name of partnership].

Informed Consent

4. Can I just check that you received the study information sheet and that you have had a chance to read it?

   No - [Review the study information sheet in detail]
   Yes - *Good* [Review the study information sheet briefly]

5. Do you have any questions about the wider study or about the interview we will be conducting today?

6. If you are happy to please can you sign the consent form that I sent you and email it back to me for our records. The consent form is a standard form used in social research and is used to ensure that:

- you understand the aims of the study
- you understand what your participation in the study will involve
- you are happy for the discussion to be audio recorded
- you know that you can change your mind about taking part in the study at any time – you can request for the discussion to stop at any time and if at any point you want to withdraw from the study you can request this by using the contact information provided to you today
- you know that your views will be kept confidential and your name will never be used in anything that is written about the study
- you consent to take part.

[Note: if the participant is not able to sign and return the consent form then consent can be recorded orally.]

[Start recording] Software will request participant to give permission to start recording. If face-to-face then request permission to start recording

K. Introductory questions

The aim of this section is to gain some contextual information about the stakeholder and their organisation, as well as the relationship between themselves/their organisation and the [name of partnership].

3. Can you tell me about your current professional role at [name of organisation] and how long you have been in post?

4. What's a typical day for you?

5. How would you describe your organisation? What is its purpose / mission? Probe around:
   - Organisational type
   - The area the organisation serves
   - Population it serves/demographic

Malley J, *et al. BMJ Open* 2022; 12:e068651. doi: 10.1136/bmjopen-2022-068651

L. Understanding of the partnership and the RPP approach

1. How were you first introduced to / come to hear about the [name of partnership]? First thoughts and impressions

2. Can you tell me about the relationship between yourself / your organisation and the [name of partnership]?
   Probe around:
   - Nature of relationship

3. How familiar are you with partnerships between research and practice? Is this partnership different to others you have seen, or other collaborations between researchers and people who work in social care organisations? Can you describe the ways in which it is different or similar?
   Probe around:
   - Do they perceive it as having a specific approach
   - Do they perceive an intention for real world change, equality, etc (see other principles)

4. What is your perception of what [name of partnership] is trying to achieve? What do you think its goals and ambitions are? Do you have any thoughts about how achievable these goals are?
   Probe around:
   - What they think might be barriers in the wider system
   - What they think might help the partnership
   - How well it fits in the system, its potential for social care and the production and use of evidence to inform and improve practice

M. Interaction with the [name of partnership]

The aim of this section is to understand more about the ways in which stakeholders are engaging within the [name of partnership]. If they are using the research then we will explore whether they are able to access and make sense of research findings and how this could be improved.

1. Can you describe the ways in which your organisation has worked with or alongside the [name of partnership]?

Malley J, *et al. BMJ Open* 2022; 12:e068651. doi: 10.1136/bmjopen-2022-068651

Probe around:

- Understanding the joint activities
- How communication takes place
- Whether there was a focus on doing or using research

Note: if there is a focus on doing and using research, explore this in more detail

2. Why did you decide to work with [name of partnership]?
   Probe around:

- Understanding what the stakeholder brings / partnership brings to stakeholder, e.g. resources, people, different perspective, potential to scale research etc
- Strategic alignment?

3. How have you found working with [name of partnership]? Have there been difficult points? What have you learnt? Would you like to work with them more?
   Probe around:

- Points of contention/consensus
- How have issues been resolved
- Learning about doing and using research in different ways, learning about practice context
- What they would like to continue working with them around

N. Exploring influence of [name of partnership] on stakeholders and vice-versa

The aim of this section is to understand the wider impact of [name of partnership] and the influence of stakeholders on the [name of partnership].

1. In what ways, if at all, has [name of partnership] had an influence on your organisation or the way you work?
   Probe around:

- outputs of the partnership e.g. used as evidence to inform decision-making
- partnership ways of working, e.g. copying partnership practices, innovations, new infrastructure etc
- Change mindsets e.g. see a greater potential for research and evidence to be used to inform practice

2. Thinking specifically about the research that [name of partnership] is doing / has done, have you found the research valuable? Can you give an example of how you have used their research and the influence this has had on your organisation or the way you work?

Malley J, *et al. BMJ Open* 2022; 12:e068651. doi: 10.1136/bmjopen-2022-068651

Probe around:

- Research agenda and priorities are of wider interest or narrow
- Look for different uses of research evidence: Changes in ideas/priorities, Direct decisions, Further research, Drawn upon materials/tools/service innovations, Justifying existing decisions
- Influence may be in changing the way they think about the potential for using evidence

3. In what ways, if at all, has [name of partnership] had an influence on the way you or your organisation think about the potential for research to inform practice? Do you have any examples of how you have changed how you work or how others work?
   Probe around:
   - Impact on their ways of working
   - Impact on the wider system

4. Do you think your organisation has had an influence on the [name of partnership]? Can you describe how you have influenced the partnership?

5. In what ways, if at all, has the work conducted in the partnership built capacity within your organisation?
   Probe around:

- Research capacity
- Other skills and knowledge
- Knowledge exchange

O. Exploring the stakeholder's views about the [name of partnership] and its value

The aim of this section is to understand how the stakeholder views the [name of partnership] and whether they see it as successful or less successful.

6. From the perspective of your role/organisation, in what ways has the [name of partnership] been successful/unsuccessful? What could they do better?
   Probe around:
   - Has it changed the way you value the RPP approach?
   - What could be improved?

Note: if there is a focus on doing and using research, explore this in more detail

7. How would you describe the [name of partnership] as a whole?
   Probe around:

- Identity/coherence

Malley J, et al. BMJ Open 2022; 12:e068651. doi: 10.1136/bmjopen-2022-068651

- Purpose
- Value
- Infrastructure

8. Can you describe the main challenges presented by factors external to [name of partnership] to it being successful?
   Probe around:
- Funding/resources
- Local and national policy
- Wider priorities and strategies within the wider social care/HEI system

9. Can you think of anything that might help the [name of partnership] to be more successful or address any challenges it is facing?
   Probe around:
- Funding/resources
- Local and national policy
- Wider priorities and strategies within the wider social care/HEI system

Malley J, *et al. BMJ Open* 2022; 12:e068651. doi: 10.1136/bmjopen-2022-068651

Co-design team interview proforma

This proforma will be used for members of the co-design team within the Creating Care Partnerships (CCP) project.

P. Introductory information

My name is XXXXXX. Thank you for agreeing to take part in the Creating Care Partnerships (CCP) study.

We are interviewing members of the codesign team to get a better understanding of the role of the co-design team within the CCP project, how your view of the RPP approach has changed over time and the support you gave to the three partnership sites.

The interview should last an hour. During the interview I will ask you about your role within the co-design team, what types of events and activities were held for the first phase of codesign and how this informed your work going forward with the sites. I will also ask you about your work with the sites, the principles you employed, and your reflections on how the sites were working to build their partnership.

I am sure there will be some areas you have more knowledge about. Where there are gaps in your knowledge it would be helpful if you could identify people or key documents that may help us to better understand the role of the co-design element of the project in supporting the sites to develop their partnerships.

Informed Consent

7. Can I just check that you received the study information sheet and that you have had a chance to read it?

   No – [Review the study information sheet in detail]
   Yes – *Good* [Review the study information sheet briefly]

8. Do you have any questions about the wider study or about the interview we will be conducting today?

9. If you are happy to please can you sign the consent form that I sent you and email it back to me for our records. The consent form is a standard form used in social research and is used to ensure that:

- you understand the aims of the study
- you understand what your participation in the study will involve
- you are happy for the discussion to be audio recorded
- you know that you can change your mind about taking part in the study at any time – you can request for the discussion to stop at any time and if at any point you want to withdraw from the study you can request this by using the contact information provided to you today

INTERVIEW PROFORMA, CCP Evaluation, Co-design team, v1.0, 13/05/2022

- you know that your views will be kept confidential and your name will never be used in anything that is written about the study
- you consent to take part.

[Note: if the participant is not able to sign and return the consent form then consent can be recorded orally.]

[Start recording] Software will request participant to give permission to start recording. If face-to-face then request permission to start recording

Q. Introductory questions about co-design team members and the role of co-design in the CCP project

The aim of this section is to gain some contextual information about the co-design team member's role in relation to the Creating Care Partnerships (CCP) project.

6. Can you tell me about your current professional role at [name of organisation] and how long you have been in post?

   How were you first introduced to the RPP approach? First thoughts and impressions

- Can you tell me in your own words what the RPP approach is and what it is trying to achieve?

- Can you tell me in your own words what the role of co-design is in the CCP project?

Explore around:
- Your role in relation to the overall aims of the project
- Your role in relation to the other CCP project teams, i.e. implementation, evaluation, spread and sustainability, lived experience reference group
- Your role in relation to the three sites that are implementing the RPP approach
- Any differences in how you have approached and carried out co-design in the CCP project compared to other projects, e.g. steps/method, aims/goals, outputs

R. First phase of co-design

The aim of this section to explore the types of activities taking place in the first phase of co-design work, what was learnt about the RPP approach from this phase, and how this learning fed into the co-design work with the case study sites.

1. Can you describe the main aims of the first phase of co-design?

2. Can you describe the process of taking the insights from the co-design activities to develop an RPP approach that is suitable for the English care home context?
   Explore around:
- How you balanced pre-existing knowledge/insights from the literature with stakeholder insight
- How your thinking changed about what the RPP approach is

3. How do you think implementing the RPP approach will play out in the sites? How will it be influenced by or influence elements of social care/HEI context? What kinds of challenges will the sites face? Do you think any elements of the RPP approach will be more achievable?
   Note: discuss social care and HEI context separately

   Explore around different levels of the system:
   - sectoral/political
   - organisational
   - professional

   Explore around the influence in relation to the following areas:
   - Building trust and relationships within RPPs
   - Individual skills, knowledge, capacity for engaging in partnership work
   - Organisational capabilities for doing research for practice improvement
   - Organisational capabilities for using research for service and system improvement

S. Second phase of co-design

The aim of this section is to explore the types of activities taking place in the second phase of co-design work, what types of support the local sites needed and the challenges of developing RPPs within the local context.

1. Can you describe the main aims of the co-design phase with the sites?

2. Can you describe how you organised the co-design sessions and your decision-making around the structure and content of the different sessions?

Probe around:
- What type of co-design activities did you run?
- What principles did you follow during this phase?
- Adapting to time and resources available within the sites, and the attitudes of the sites

3. How have the sites responded to the co-design process and activities?

Note: discuss each site separately

Probe around:

- What challenges did they have/what seemed to come easier?
    - in relation to the co-design materials and the activities
    - in relation to their understanding of the RPP approach
    - in relation to their understanding of how to develop their partnership in a way that is consistent with the RPP approach
- How did the different members of the partnership work together and with the co-design team? Any differences in degree of participation? Were some people more vocal? Did people defer to particular members of the partnership?

4. Do you have any sense of how the sites have used/are using the co-design sessions to inform the way they are developing their partnership?

Note: discuss each site separately

Probe around:

- Any feedback from the sites / follow-up
- Any indications from discussions in the sessions
- Did they bring plans or start planning in the sessions?

5. Did you have any sense of how the site's local context might be influencing their engagement with the co-design work?

Note: discuss each site separately

Probe around:

- What they want to discuss?
- Ability to work with the co-design team or together?

6. You have said your approach to working with the sites is to be more facilitative, so the co-design team do not become an extension of the partnership at each site. How has that intention worked in practice?

Note: discuss each site separately

Probe around:

- Was there an evolution from front-loaded support to gradually stepping back?
- Have the sites approached you for additional support, e.g. where progress has stalled? How have you managed that process?
- How did you decide when to give support and when to step back?
- Have there been any really challenging moments? Can you describe what the issue was and how it played out?

Malley J, *et al. BMJ Open* 2022; 12:e068651. doi: 10.1136/bmjopen-2022-068651

CCP implementation team interview proforma

This proforma will be used for members of the implementation team within the Creating Care Partnerships (CCP) project.

T. Introductory information

My name is XXXXXX. Thank you for agreeing to take part in the Creating Care Partnerships (CCP) study.

We are interviewing members of the implementation team to get a better understanding of the role of the implementation team within the CCP project and support you have given the sites as they are developing their partnership.

The interview should last an hour. During the interview, I will ask you about your role in the implementation team, how you view the RPP approach and the influence of wider contextual factors on applying this approach within social care. I will also ask you about the activities you have carried out to support the sites and your reflections on how the sites are developing their partnership.

I am sure there will be some areas you have more knowledge about. Where there are gaps in your knowledge it would be helpful if you could identify people or key documents that may help us to better understand the implementation element of the project.

Informed Consent

10. Can I just check that you received the study information sheet and that you have had a chance to read it?

    No - [Review the study information sheet in detail]
    Yes - *Good* [Review the study information sheet briefly]

11. Do you have any questions about the wider study or about the interview we will be conducting today?

12. If you are happy to please can you sign the consent form that I sent you and email it back to me for our records. The consent form is a standard form used in social research and is used to ensure that:

- you understand the aims of the study
- you understand what your participation in the study will involve
- you are happy for the discussion to be audio recorded
- you know that you can change your mind about taking part in the study at any time – you can request for the discussion to stop at any time and if at any point you want to withdraw from the study you can request this by using the contact information provided to you today
- you know that your views will be kept confidential and your name will never be used in anything that is written about the study

INTERVIEW PROFORMA, CCP Evaluation, Implementation team, v1.0, 13/05/2022

- you consent to take part.

[Note: if the participant is not able to sign and return the consent form then consent can be recorded orally.]

[Start recording] Software will request participant to give permission to start recording. If face-to-face then request permission to start recording

U. Introductory questions about co-design team members and the role of implementation team in the CCP project

The aim of this section is to gain some contextual information about the implementation team member's role in relation to the Creating Care Partnerships (CCP) project. It is also to gain an understanding of how the implementation team view the RPP approach and the ways it will relate to the social care/HEI context.

1. Can you tell me about your current professional role at [name of organisation] and how long you have been in post?

2. Can you tell me in your own words what the role of the implementation team is in the CCP project?

Probe around:
- Your role in relation to the overall aims of the project
- Your role in relation to the other CCP project teams, i.e. co-design, evaluation, spread and sustainability, lived experience reference group
- Your role in relation to the three sites that are implementing the RPP approach

3. Can you tell me in your own words what the RPP approach is and what it is trying to achieve?

   Probe around:
   - First thoughts and impressions
   - Core principles
   - Whether your views on this have changed following co-design work

4. How do you think implementing the RPP approach will play out in the sites? How will it be influenced by or influence elements of social care/HEI context? What kinds of challenges will the sites face? Do you think any elements of the RPP approach will be more achievable?
   Note: discuss social care and HEI context separately

   Explore around different levels of the system:
   - sectoral/political
   - organisational
   - professional

Explore around the influence in relation to the following areas:
- Building trust and relationships within RPPs
- Individual skills, knowledge, capacity for engaging in partnership work
- Organisational capabilities for doing research for practice improvement
- Organisational capabilities for using research for service and system improvement

C. Main activities and ways of working with the sites

The aim of this section is to explore the types of activities that the implementation team are engaged in to support the sites, the main challenges, and the impact.

1. Can you describe the main aims of the implementation work you are doing with the sites?
   Probe around whether there are any differences across the sites

2. Can you tell me about the activities you have been involved in to support sites and the main aims of these activities?
   Probe around whether there are any differences across the sites and around the following areas:
- Infrastructure support
- Research skills
- Agenda setting
- Communication skills
- Building relationships/managing conflict

3. Can you tell me about the types of support that the sites have asked for?
   Probe around whether there are any differences across the sites and around the following areas:
- Types of questions
- Formal/informal support
- Important/least important support needed
- Difference in support needs depending on academic/care home partners

4. How are the sites responding to the support you have given?

Note: discuss each site separately
Probe around:
- Your experiences of how receptive the sites are to suggestions and ideas
- How sites approach their interactions with you, i.e. collaborative, antagonistic, distant
- Difference between partners in how much they engage
- What challenges did they have/what seemed to come easier?
    - in relation to the co-design materials and the activities

INTERVIEW PROFORMA, CCP Evaluation, Implementation team, v1.0, 13/05/2022

   - in relation to their understanding of the RPP approach
   - in relation to their understanding of how to develop their partnership in a way that is consistent with the RPP approach

5. Can you describe how the local partnership context is influencing the type of support they need?
   Note: discuss each site separately
   Probe around:
- Local geography/particular needs of the population served
- Composition of partnership
- Care home type
- Existing infrastructure

6. Do you have any sense of how the sites have used/are using the implementation support to inform the way they are developing their partnership?
   Note: discuss each site separately
   Probe around:
- Any feedback from the sites / follow-up
- Any indications from discussions in the sessions
- Did they bring plans or start planning in the sessions?

7. You have said your approach to working with the sites is to be more facilitative, so the implementation team do not become an extension of the partnership at each site. How has that intention worked in practice?
   Note: discuss each site separately
   Probe around:
   - Was there an evolution from front-loaded support to gradually stepping back?
   - Have the sites approached you for additional support, e.g. where progress has stalled? How have you managed that process?
   - How did you decide when to give support and when to step back?
   - Have there been any really challenging moments? Can you describe what the issue was and how it played out?

D. Reflections on how the partnerships are working in practice

The aim of this section is to understand how the implementation team perceive the way in which partnership sites are working together, their ability to assess their own needs, and how contextual factors may be influencing their success.

1. Can you describe how partnership members are working together?
   Note: discuss each site separately
   Probe around:
   - Ways in which trust and relationships are building
   - Power dynamics between the partners, are some members more dominant than others?

INTERVIEW PROFORMA, CCP Evaluation, Implementation team, v1.0, 13/05/2022

- Whether partnerships have developed intentional strategies to manage unequal power dynamics

2. How well do the sites understand the RPP approach? What types of strategies are they putting in place to implement the approach?
   Note: discuss each site separately
   Probe around:
   - Strategies for conducting research to meet the problems of practice
   - Level of understanding and strategies for setting organisational and broader goals
   - Building capacity for joint boundary work
   - Focus on organisational goals vs wider system impact
   - Variation between different types of partners

3. How well are sites able to identify problems and the support they need?
   Note: discuss each site separately
   Probe around:
   - Ability to understand the knowledge, expertise & skills of different partners
   - Overcoming differences of opinion or ensuring the right mix of voices are there

4. In your opinion do the sites need more or different types of support to what has been identified? Are there problems within the partnership that have not been raised?
   Note: discuss each site separately
   Probe around:
   - Organisational contextual factors
   - Partnership composition
   - Partnership internal dynamics

5. In what ways do you think the sites have been affected by the social care/HEI context, and how has this affected their success in implementing the RPP approach?
   Note: discuss each site separately, and discuss social care & HEI context separately

Lived experience lead interview proforma

This proforma will be used for the Lived Experience Reference Group (LERG) of the Creating Care Partnerships (CCP) project.

V. Introductory information

My name is XXXXXX. Thank you for agreeing to take part in the Creating Care Partnerships (CCP) study.

We are interviewing the lived experience reference group lead to get a better understanding of the role of the lived experience reference group within the CCP project and the support the group has given the sites as they are developing their partnership.

The interview should last an hour. During the interview, I will ask you about the role of the lived experience reference group, how you view the RPP approach and the influence of wider contextual factors on applying this approach within social care. I will also ask you about the activities the group has carried out to support the CCP sites and your reflections on how the sites are developing their partnership.

I am sure there will be some areas you have more knowledge about. Where there are gaps in your knowledge it would be helpful if you could identify people or key documents that may help us to better understand the lived experience element of the project.

Informed Consent

13. Can I just check that you received the study information sheet and that you have had a chance to read it?

    No - [Review the study information sheet in detail]
    Yes - *Good* [Review the study information sheet briefly]

14. Do you have any questions about the wider study or about the interview we will be conducting today?

15. If you are happy to please can you sign the consent form that I sent you and email it back to me for our records. The consent form is a standard form used in social research and is used to ensure that:

- you understand the aims of the study
- you understand what your participation in the study will involve
- you are happy for the discussion to be audio recorded
- you know that you can change your mind about taking part in the study at any time – you can request for the discussion to stop at any time and if at any

point you want to withdraw from the study you can request this by using the contact information provided to you today
- you know that your views will be kept confidential and your name will never be used in anything that is written about the study
- you consent to take part.

[Note: if the participant is not able to sign and return the consent form then consent can be recorded orally.]

[Start recording] Software will request participant to give permission to start recording. If face-to-face then request permission to start recording

W. Introductory questions about the lived experience reference group and the role of the group in the CCP project

The aim of this section is to gain some contextual information about the LERG's role in relation to the Creating Care Partnerships (CCP) project. It is also to gain an understanding of how the LERG lead views the RPP approach and the ways it will interact with the social care/HEI context.

5. Can you tell me about your current professional role at [name of organisation] and how long you have been in post?

6. How were you first introduced to the RPP approach? First thoughts and impressions

7. Can you tell me in your own words what the RPP approach is and what it is trying to achieve?
   Explore around:
   - Core principles
   - Whether your views on this have changed following co-design work

8. Can you tell me in your own words what the role of the lived experience reference group within the CCP project?

Explore around:
- How members were recruited and what you were looking for
- The group's role in relation to the overall aims of the project
- The group's role in relation to the other CCP project teams, i.e. co-design, evaluation, implementation, spread and sustainability
- The group's role in relation to the three sites that are implementing the RPP approach
- What's not within their remit

9. How do you think implementing the RPP approach will play out in the sites? How will it be influenced by or influence elements of social care/HEI context?

INTERVIEW PROFORMA, CCP Evaluation, Lived experience lead, v1.0, 13/05/2022

What kinds of challenges will the sites face? Do you think any elements of the RPP approach will be more achievable?
Note: discuss social care and HEI context separately
Explore around different levels of the system:

- sectoral/political
- organisational
- professional

Explore around the influence in relation to the following areas:

- Building trust and relationships within RPPs
- Individual skills, knowledge, capacity for engaging in partnership work
- Organisational capabilities for doing research for practice improvement
- Organisational capabilities for using research for service and system improvement

C. Main activities and ways of working with CPP teams and the sites

The aim of this section is to explore the types of activities that the LERG are taking part in to support the sites, the challenges of this, and the impact.

8. Can you tell me about the activities you have been involved in to support the sites?
Note: discuss the sites separately, if applicable
Probe around:

- Site specific feedback on development of partnership, their research etc
- Indirect support to sites through working with the co-design or implementation team to make activities accessible, etc

9. Can you tell me about the types of support that the sites have asked for or the wider CCP team to support their work with the sites?
Note: discuss the sites separately, if applicable
Probe around:

- Types of issues
- Difficulties with any requests

10. Can you describe the benefit/value of the work the group has done so far?
Note: discuss the sites separately, if applicable
Probe around:

- Ways in which advice was received and incorporated
- Times when it has been less valuable
- Additional ways that the group could assist the sites or CCP team members in working with the sites

INTERVIEW PROFORMA, CCP Evaluation, Lived experience lead, v1.0, 13/05/2022

11. How are the sites responding to the support you have given?
    Note: this question relates just to the sites so as to avoid evaluating CCP colleagues
    Probe around:

- Your experiences of how receptive the sites are to suggestions and ideas
- How sites approach their interactions with you, i.e. collaborative, antagonistic, distant
- Difference between partners (care home/academic) in how much they engage

12. Can you describe any challenges faced by the lived experience reference group?
    Probe around:

- Occasions when feedback was resisted or not taken on board
- Skills or specialisms of LERG members

13. Can you describe how the local partnership context influenced the type of support the sites needed?
    Note: discuss each site separately
    Probe around:

- Local geography/particular needs of the population served
- Composition of partnership
- Care home type
- Existing infrastructure and lived experience input

14. Can you describe how the RPP approach influenced the type of support the LERG provided to the sites/the wider CCP project team to support the sites?
    Note: discuss the sites separately, if applicable
    Probe around*:*
    - Core principles of RPP
    - Idea that it is based on bottom-up development
    - Ways this may differ to providing lived experience advice on other research projects

E. Reflections on how the partnerships are working in practice

The aim of this section is to understand how the lived experience lead perceives the way in which the sites are working with residents and family members, their ability of the sites to assess their own needs and how contextual factors may be influencing their success.

6. Can you describe how partnership members are working with care home residents and their families/friends?
   Note: discuss each site separately

Probe around:
- Ways in which trust and relationships are building
- Power dynamics between the partners, are some members more dominant than others?
- Whether partnerships have developed intentional strategies to manage unequal power dynamics

7. How well were sites able to identify problems and what lived experience support they needed?
   Note: discuss each site separately
   Probe around:
   - Ability to assess where they were missing lived experience input and the consequences this was having

8. In your opinion what types of lived experience support do the sites need but have not identified? What are the barriers to this?
   Note: discuss each site separately
   Probe around:
   - Organisational contextual factors
   - Partnership composition
   - Partnership internal dynamics
   - Factors which relate to the social care context

INTERVIEW PROFORMA, CCP Evaluation, Lived experience lead, v1.0, 13/05/2022
