# Supplementary material for: Theory-based evaluation of three research–practice partnerships designed to deliver novel, sustainable collaborations between adult social care research and practice in the UK: a research protocol for a ‘layered’ contributions analysis and realist evaluation
Source: BMJ Open. 2022 Nov 25;12(11):e068651. doi: 10.1136/bmjopen-2022-068651 (PMC9703321; doi:10.1136/bmjopen-2022-068651)
Supplement: Author's manuscript [file bmjopen-2022-068651.draft_revisions.pdf]

BMJ Open is committed to open peer review. As part of this commitment we make the peer review history of every article we publish publicly available.

When an article is published we post the peer reviewers' comments and the authors' responses online. We also post the versions of the paper that were used during peer review. These are the versions that the peer review comments apply to.

The versions of the paper that follow are the versions that were submitted during the peer review process. They are not the versions of record or the final published versions. They should not be cited or distributed as the published version of this manuscript.

BMJ Open is an open access journal and the full, final, typeset and author-corrected version of record of the manuscript is available on our site with no access controls, subscription charges or pay-per-view fees (http://bmjopen.bmj.com).

If you have any questions on BMJ Open's open peer review process please email info.bmjopen@bmj.com

BMJ Open

A theory-based evaluation of three Research Practice Partnerships designed to deliver novel, sustainable collaborations between adult social care research and practice in the UK: a research protocol

Journal:	*BMJ Open*
Manuscript ID	bmjopen-2022-068651
Article Type:	Protocol
Date Submitted by the Author:	26-Sep-2022

Complete List of Authors:	Malley, Juliette; The London School of Economics and Political Science, Care Policy and Evaluation Centre<br>Bauer, Annette; London School of Economics and Political Science, Care Policy and Evaluation Centre<br>Boaz, Annette; London School of Hygiene & Tropical Medicine<br>Kendrick, Hannah; London School of Economics and Political Science, Care Policy and Evaluation Centre<br>Knapp, Martin; The London School of Economics and Political Science, Care Policy and Evaluation Centre

Keywords:	Health policy < HEALTH SERVICES ADMINISTRATION & MANAGEMENT, Organisational development < HEALTH SERVICES ADMINISTRATION & MANAGEMENT, Quality in health care < HEALTH SERVICES ADMINISTRATION & MANAGEMENT

SCHOLARONE™
Manuscripts

Full title: A theory-based evaluation of three Research Practice Partnerships designed to deliver novel, sustainable collaborations between adult social care research and practice in the UK: a research protocol

Short title: Creating Care Partnerships Evaluation

Juliette Malley[1], Annette Bauer[2], Annette Boaz[3], Hannah Kendrick[4], Martin Knapp[5]

Corresponding Author:

[1] Juliette Malley j.n.malley@lse.ac.uk; Care Policy and Evaluation Centre (CPEC), London School of Economics and Political Science, Houghton Street, London WC2A 2AE, UK

Co-authors:

[2] Annette Bauer a.bauer@lse.ac.uk; Care Policy and Evaluation Centre (CPEC), London School of Economics and Political Science, Houghton Street, London WC2A 2AE, UK

[3] Annette Boaz annette.boaz@lshtm.ac.uk; London School of Hygiene and Tropical Medicine, 15-17 Tavistock Place, London WC1H 9SH, UK

[4] Hannah Kendrick h.kendrick@lse.ac.uk; Care Policy and Evaluation Centre (CPEC), London School of Economics and Political Science, Houghton Street, London WC2A 2AE, UK

[5] Martin Knapp M.Knapp@lse.ac.uk; Care Policy and Evaluation Centre (CPEC), London School of Economics and Political Science, Houghton Street, London WC2A 2AE, UK

KEYWORDS

care homes; research-practice partnerships; theory-based evaluation; economic evaluation

ACKNOWLEDGEMENTS

We would like to acknowledge and thank our Creating Care Partnerships project colleagues (Bev Fitzsimons, Joe Langley, Stuart Muirhead, Becca Partridge, Naomi Raszyk, Lisa Smith, Melanie Weatherley and Claire Williams), participants in the co-design workshops and the project public advisors for their contribution to the development of the theory of change and data collection materials.

For peer review only - http://bmjopen.bmj.com/site/about/guidelines.xhtml

Word count: 5,660 words

ABSTRACT

Introduction

Research practice partnerships (RPPs) are long-term collaborations between research and practice that aim to conduct research that can be used to make practice-based improvements. They intentionally bring together diverse experience in decision making and seek to shift power dynamics so that all partners have a say. The Creating Care Partnerships project aims to explore whether the RPP approach developed within the US educational context can be successfully applied to the English care home context. The project involves a programme of co-design, implementation, and evaluation within three case study sites. This protocol set outs the aims, research design, and governance of the evaluation.

Methods and analysis

The evaluation takes a theory-based approach to explore how, why and in what circumstances RPPs in the care home context contribute to enhancing research and research use in local care homes and informing wider improvement efforts. A mixed methods design will be used for each case study, including semi-structured interviews, observations of RPP events and meetings, an online survey, activity diary and review of local data and documents. Data collection will proceed in waves, with the theory of change (ToC) being continually refined and used to guide further data collection and analysis. Insights will be drawn using Contribution Analysis, Realist Evaluation, and systems perspectives to assess the contribution made by the case study sites to achieving outcomes and the influence of contextual factors. Economic consequences will be identified through the ToC, using a narrative economic analysis to assess costs, consequences, and value for money.

Ethics and dissemination

The study has undergone ethics review by HRA Research Ethics Committee. It does not pose major ethical issues. A final report will be published and articles will be submitted to international journals.

STRENGTHS AND LIMITATIONS OF THIS STUDY

- A theory-based approach allows for greater explanation of how RRPs work, whilst appreciating the complexity and non-linearity of implementation.
- The use of mixed methods allows us to draw on the strengths of different methods, improving the credibility of evaluation findings.

For peer review only - http://bmjopen.bmj.com/site/about/guidelines.xhtml

- An economic evaluation will allow policymakers and funders to make evidence-based decisions about the value of further investment in RPPs for the care home sector.
- The evaluation period may not be long enough to capture the extent to which outcomes have been achieved within either local care homes or the wider care ecosystem.
- Theory-based approaches, including contributions analysis and realist evaluation that are used here, are not as well-established as other approaches for establishing effectiveness. This study may draw criticism for not being as rigorous as experimental approaches.

For peer review only

INTRODUCTION

An important question for adult social care (ASC) is how investment in research can be optimised to support improvements for people drawing on social care services. Despite significant investment and a growing evidence base [1], the evidence produced is in general poorly used by the ASC practice community (e.g. social care commissioners, providers and the workforce) [2]. There is a growing interest in approaches that focus on building relationships and stronger links between those who produce research, those who use research and the intended beneficiaries to complement and augment existing efforts and infrastructure investments [3–5]. The Creating Care Partnerships (CCP) project aims to re-design for the care home context and implement a promising approach called Research Practice Partnerships (RPPs) in three sites across England [6]. Reflecting the central place learning has in the CCP project, it also includes an evaluation of the new RPPs. This paper describes the evaluation protocol.

RPPs are a specific form of partnership that offer a different way of producing and mobilising research that fundamentally challenges the status quo. They are long-term collaborations between research and practice communities that aim to bring about real-world change through the use of research evidence. RPPs can vary in scope and size and may have different strengths, but not all research-practice collaborations are RPPs [7,8]. To be an RPP, collaborative efforts must extend beyond the life of a single research study or project and must engage with research as a core activity. Another feature of RPPs is the intentional integration of expertise from two communities -- practice and research – that are often disconnected. Relatedly RPPs engage in activities to shift power relations to ensure everyone has a say in the research endeavours; people from practice communities are involved from the outset, and both communities contribute equitably to shaping the direction of the work and supporting the use of what is learned from the research [8]. Although a substantial corpus of research has developed that describes the core principles of an RPP with lessons for those seeking to reproduce it (see NNERPP RPP knowledge clearinghouse https://nnerpp.rice.edu/rpp-knowledge-clearinghouse/, William T. Grant microsite https://rpp.wtgrantfoundation.org/), questions remain around what effective partnering looks like [8,9]. How well RPPs meet their goals and the conditions that support or hinder their progress are seen as pressing issues for research [8,10,11].

The main question for the CCP evaluation is whether this approach, which has been developed within the US educational context, can be successfully adapted for and implemented within the English care home context with similarly positive results in terms of driving improvements in practice and in the wellbeing of recipients of social care services. The primary aim of the CCP evaluation is therefore to provide evidence about the effectiveness of RPPs in the care home context; but, with a view to ensuring a legacy from the project, a second aim is to gather evidence about how to implement and sustain the approach so it can be reproduced elsewhere. The English care home context is very different to the US education context – a key difference being the lack of professionalisation and lower educational attainment of the majority of the care home workforce compared to educators, but there are also differences in the higher education contexts between the two countries and the

For peer review only - http://bmjopen.bmj.com/site/about/guidelines.xhtml

research and innovation infrastructure. We expect this evaluation to deepen understanding of the way in which local conditions affect how RPPs function, the kinds of strategies they need to leverage to enact the RPP guiding principles and, possibly, what RPPs look like, with lessons for the international RPP community. Given the economic context and existing investments in research and knowledge mobilisation, a third aim of the evaluation is to understand the desirability of further investment in RPPs given the costs and the value that flows from the investment. Since the question of the economic value of RPPs is only beginning to be considered [12], this element of the evaluation is novel and will contribute to developing schemas for assessing value.

The CCP co-design work has produced a set of guiding principles for RPPs operating in the care home context that will be operationalised in different ways by each new RPP. Reflecting the strongly theoretical and complex nature of RPPs, our evaluation perspective is theory-based and draws on a systems perspective [13]. It addresses the following questions (and sub-questions):

1. How, why and in what circumstances do RPPs in the care home context contribute to enhancing research and research use in local care homes and informing wider care home improvement efforts?
    a. To what extent have the main outcomes been achieved?
    b. How significant is the contribution of the CCP partnership to the main outcomes, given other factors?
    c. How, why and in what circumstances do the CCP partnerships contribute to each outcome?
    d. To what extent is the way the CCP partnerships operate consistent with the RPP approach?
2. What are the costs of delivering RPPs in the care home context, and are they good value for money?

It is not yet standard practice to publish protocols for evaluations of the kind outlined here. Our intention in publishing this protocol is to increase transparency in our methods and encourage discussion around them.

THEORETICAL FRAMEWORK

We use the evidence-based framework developed by Henrick and colleagues [10] and adapted to the care home context as a framework for the evaluation. It identifies five dimensions of outcomes for successful RPPs: (1) building trust and cultivating partner relationships, (2) producing relevant research that is used, (3) supporting the practice organization in achieving its goals, (4) producing knowledge that can inform social care practice improvements more broadly, and (5) building the capacity of participants to engage in the partnership work [9]. This framework provides a focus for measurement of RPP effectiveness and the integration of findings across the sites, but it can also inform the development and sustainability of such partnerships and theories of change [14–16].

A key strength of the framework is that it is flexible enough to allow multiple theories to inform the evaluation, which existing research indicates will be necessary to understand how and why RPPs work. RPPs have been conceptualised in a variety of

For peer review only - http://bmjopen.bmj.com/site/about/guidelines.xhtml

ways [11,15], but there is a consensus around the notion of RPPs as engaged in joint work at boundaries [17,18]. In addition to theories of boundary infrastructure (including boundary spanners, practices and objects), scholars have drawn on organisational theories (including absorptive capacity, organisational learning and organisational routines) to explain how RPPs successfully produce and use research that improves practice within the practice organisation and has wider sectoral impacts [17–19]. Since working across boundaries is challenging [11], many of the conceptual contributions also focus on relational aspects, including building trust, redistributing power, conflict and consensus, identity and role negotiation, and leadership [18,20–22]. Theoretical contributions that help to analyse different types of power and how they are distributed [23] and how new identities are formed or resisted [24] may prove useful.

Evidence from existing RPPs suggests that it takes time for them become productive and embedded within the wider higher education and practice ecosystems [14]. As RPPs start to have an impact on and beyond the partnering organisations it may be useful to conceptualise them as 'events in systems' [25]. This perspective draws attention to the fact that RPPs are not neutral additions to the ecosystem but through their intention to have broader impact on care practice and how research is done, they challenge the status quo and in complex ways will interact with, shape and be shaped by these wider contexts. Understanding how the RPP and other players in the wider ecosystem (e.g. higher education institutions, funding bodies, local authorities) interact with each other over time is key to understanding the potential for sustainability and spread of RPPs, as to endure RPPs must become resilient to shocks from within (e.g. organisational turnover, conflicts, and competing organisational norms) and, crucially, shocks from outwith the partnership (e.g. changes in policy, economic shocks, changes in funding) [26].

DESIGN AND METHODS FOR THE THEORY-BASED EVALUATION

The evaluation is theory-based and employs a multiple case study design with longitudinal data collection. There are various approaches to theory-based evaluation, which differ in their methods for constructing a valid ToC or programme theory and the types of questions for which they are designed to respond [27–31]. In this evaluation, we use contribution analysis, and realist evaluation sequentially as 'layered tactics' to address different sub-questions [32].

The contribution analysis lens addresses whether the RPPs make a meaningful contribution to enhancing research and research use in care homes and the wider system (1b), by taking into account other factors and rival explanations [29,33,34]. It produces credible causal claims about the contribution RPPs make to observed outcomes allowing us to draw conclusions about whether RPPs are a promising approach in the English care home context. By contrast through realist methods, we can probe in greater depth the different ways in which the CCP partnerships may implement the RPP approach, the circumstances that may affect the choices they make and the outcomes observed (1c). Realist evaluation explores causality through developing and testing programme theories as context-mechanism-outcome (CMO)

For peer review only - http://bmjopen.bmj.com/site/about/guidelines.xhtml

configurations, which explain how and why RPPs might trigger different change mechanisms across different contexts to achieve (or not achieve) outcomes [31,35]. The three partnerships were selected through an open competition to offer contrasting situations. They differ in the size, scope, types of partners and local context, which will allow us to explore conducive or inhibiting contexts for RPPs [36]. Should it prove useful for mapping the complexity of the RPPs' context and refining our ToC (e.g. because the RPPs are actively seeking to have impact beyond their sites) we will add a third soft systems lens [36].

Stage 1: Development of the initial theory of change and hypotheses

For theory-based evaluations, the theory of change (ToC) or programme theory plays a central role in assuring the quality of the evaluation. It guides the measurement of concepts and the investigation of causal relationships between the activities of RPPs, outputs and outcomes. There is guidance for developing and testing ToC/programme theory for contributions analysis and realist evaluation that we will follow [33,37–42]. The ToC and hypotheses about CMO combinations will be informed by the literature on RPPs in the US education context, similar partnerships between research and practice in ASC and related fields, and insights about how the approach might translate to the English care home context gathered through the co-design work conducted as part of the first stage of the CCP project.

Based on an initial review, Figure 1 sets out a ToC for RPPs in the care home context. In setting out the theoretical causal chain through which activities/outputs lead to improvements in care practice in the care homes and the wider care ecosystem and the assumptions that need to be met for this chain of events to come about, we draw heavily on Farrell et al.'s work [18]. Influencing factors are drawn from our knowledge of the sector and discussions with the co-design workshops. We also illustrate in Figure 1 the relationship between the ToC and the five dimensions of effectiveness, which guide measurement [10].

[Figure 1: A theory of change for research-practice partnerships in adult social care]

Figure 1 is a preliminary ToC and a stepping-stone towards developing initial CMO hypotheses. The ToC does not illustrate well the trajectories to economic impact, hypotheses about how the context might trigger certain mechanisms and outcomes, nor our expectation that the journeys for each RPP will resemble a 'ripple effect', in that outcomes from earlier activities may form the context for later activities and outcomes [43]. Ultimately, systems diagrams with feedback loops to identify how inputs, activities and the outputs of those activities affect stocks of trust, and organisational capabilities to produce and use research, may be useful, as might participatory systems and ToC mapping approaches with the sites to identify economic value and understand complex local systems [43,44]. The use of such approaches, however, will depend on willingness and progress made by the sites.

For peer review only - http://bmjopen.bmj.com/site/about/guidelines.xhtml

Stage 2: Conducting the evaluation through testing and refining theory

We will use a mixed-methods design for each case study [45], including semi-structured interviews, observation of RPP events and meetings, an online survey, activity diary and review of local data and documents. The different methods allow for evaluation of a broader range of outcomes, unintended consequences, and provide greater confidence in the measurement of key constructs and the evidencing of claims about the effectiveness of the new CCP partnerships. This approach is common among empirical studies of research use to make sure it is not over-estimated [46], and has been widely employed in RPP studies [7,10,47]. Data will be collected in multiple rounds and will guided by ongoing refinement of the ToC/programme theory.

To ensure we gather a range of perspectives on the development of the CCP partnerships, we will seek insights from members of RPPs (i.e. researchers, residents and family members, care home staff and other professionals who participate in the partnership) and wider stakeholders. These are people not directly involved in partnership work but who have a key stake in its success or influence its progress. They could include the university leadership, local authority staff (commissioners, social workers), owners or directors of care home groups, local trade associations, CQC inspectors, etc.

Survey

A web-based survey, designed using Qualtrics, will be sent to all RPP members at each partnership, on a roughly six-monthly basis, starting at baseline and around three to four further times over the project. As partnerships are small (we expect around ten people), the survey will take an enumeration sample of partnership members. The research team will send two reminders over a 6-week period to maximise response rates.

The questionnaire builds on a validated tool to evaluate the progress of US RPPs in the education context against the five outcome dimensions [14], but includes adaptations and changes to fit this context and address the research questions. The questionnaire captures trust and perceptions of relationships; whether the partnership has routines for doing and using research (capturing organisational capabilities); participation in partnership activities (capturing boundary working); relevance of research to practice; whether the partnership is achieving its goals; the impact of the partnership on the care home practitioners and their practice, and the wider sector; evidence of investment in the partnership and its members to enable everyone to participate fully.

To capture contextual factors of influence, we also included a question on individual skills and knowledge to participate in the partnership; a set of questions of particular relevance to practitioners on attitudes to research [48], and four-item personal research skills and knowledge sub-scale from the R&D culture index [49]; a set of questions of particular relevance to researchers on personal co-production skills and knowledge [50]; a set of questions on their employer’s (not the RPP’s) research

For peer review only - http://bmjopen.bmj.com/site/about/guidelines.xhtml

culture [48] and culture with respect to co-production; and a set of questions on identification with and commitment to the RPP that draw on Mael and Ashforth's [51] six-item scale of organisational identification and four items from Meyer and Allen's [52] affective commitment scale, dropping an item that could not be translated to this context and another that is not considered part of affective commitment [53]. In the final survey wave, for those questions that ask respondents to judge their skill or experience level, we will consider using retrospective pre-tests that allow respondents to rate themselves retrospectively from the beginning of the partnership compared with at the time of the final survey. This has been found to remove response shift bias and provide a more valid result than traditional pre- and post-test ratings when respondents are providing self-evaluations of their knowledge [54].

A survey of stakeholders might be warranted to capture what they view as the significance and value of partnership work. As its value depends on progress of the RPPs, it is not currently planned.

Interviews with CCP partnership members, wider stakeholders and CCP team members

For each partnership, we will hold one-hour long semi-structured interviews with CCP partnership members and stakeholders at the start of the partnership process and at three points thereafter. The number of interviews will be determined on a case-by-case basis, based on involvement in activities of the partnership, influence over the operation of the partnership and their ability to inform the research, but for CCP partnership members, it is likely to be around seven to eight interviews at each wave and for stakeholders around three. Initially RPP members and stakeholders will be chosen in consultation with the main site contact, but in order to minimise selection bias and the marginalization of people, a "snowballing" identification practice will be implemented.

Inevitably, as the partnerships develop the focus of the interviews will shift from capturing the setting up of the partnerships, to doing research as an RPP, and then to using research for organisational learning, wider knowledge exchange and impact beyond the partnership. Topic guides will be informed by the ToC and hypotheses about CMO combinations. Prompts and probes will ensure we explore power dynamics, trust, and the wider organisational and system context characterised by competing interests and values in shaping the trajectory of these partnerships.

We will also conduct one-hour semi-structured interviews with members of the CCP team who are leading the co-design, implementation and user and stakeholder involvement activities shortly after the co-design process has ended, and about three further times over the course of the implementation phase in broad alignment with the timing of support activities. The aim of these interviews is to capture the CCP team members' experience of delivering support activities to the partnerships and their views on how the partnerships are responding to the support, developing and using the support to shape their partnerships.

All interviews will be audio-recorded and transcribed.

For peer review only - http://bmjopen.bmj.com/site/about/guidelines.xhtml

Observation

We will conduct observation of partnership events for each case study to understand how the work of RPPs is being carried out in practice. The observations will focus on interactions, such as how the RPP members and attendees at events work together, make decisions and put their ideas and strategies into practice, and will be informed by an observational framework. Field notes will be written up for each event observed.

Activity diaries

To capture the time RPP members spend on different partnership activities, they will complete an activity diary. To facilitate entry in real-time, we propose that they use an existing time tracking app (Harvest, http://www.getharvest.com). Data will be visualised on an ongoing basis using the app and downloaded on a monthly basis.

We have included a development and testing phase to explore the best way of reporting activities and the feasibility of using the app at each site. Given the different types of activities that partnerships might use to enact RPP principles, we will use this phase to build up a categorisation for data entry. It will also help us understand the best way to integrate the collection of these data into RPP members' routines to ensure high quality data [55]. We will hold a workshop with each CCP partnership to develop solutions to these and other issues that members may have, including access to smartphones.

We will develop tailored guidance for the CCP partnerships and training that can be rolled out if new members join the partnership during the evaluation.

Routine and project-related data and local documents

Routine data, data related to research projects and documents produced by, for or about the partnership will be collected from each partnership on a regular basis. The aim of collecting this information is to provide insight into the plans for and activities of the partnership, research it is producing and using, and the relationship between the partnership and its parent and other organisations.

At this stage it is difficult to say what the information might look like, as it will be highly dependent on the plans and research agenda for each site. Based on learning from the interviews and observations, we will develop a template of the types of information we will request from the CCP partnerships on a quarterly basis. Examples might include, meeting minutes, data analysis notes, tools developed, grant proposals. This will ensure a degree of consistency in what we request from sites. The template will be reviewed and revised as the partnerships develop.

Reflecting on evaluation practice

The aim of the evaluation is not to provide a definitive judgement about the effectiveness of each RPP. We recognise that RPPs are on a journey and judgements about their value would be time-bound and unstable [56]. Instead, we

For peer review only - http://bmjopen.bmj.com/site/about/guidelines.xhtml

aim to learn more about what can be achieved through the RPP approach as it is introduced in a new context and how the principles can be successfully enacted.

Although we are not providing a definitive judgement about the effectiveness of each new RPP, we will need to reflect critically on our practices and be sensitive to the ways in which they and the evidence we produce might influence the ways in which the CCP partnerships develop [57]. In this vein, we do not take a formative approach to the evaluation. This is in part to ensure a degree of independence, but more importantly, it ensures that our activity does not prevent the new partnerships from developing their own capacity to monitor and evaluate their work, since this might in the long-run undermine the sustainability of the partnership. We do recognise, however, that our evaluative judgements will be of value to the sites and have planned several feedback sessions. This will need to be situated within processes for learning and action, and will be delivered with the CCP implementation, and sustainability and spread teams. These workshops will enable sites to learn from the evaluation and use the information to improve how their partnerships are working.

PPI statement

The CCP project includes a public member as part of the Management Team, who has the role of Involvement Lead. The involvement lead contributed to the development of the proposal, methods, and will advise the team throughout the various phases of the project.

The study has a lived experience reference group, comprising people with lived experience of receiving and giving care. Their main role is to provide support to the sites to involve the public and people with lived experience of giving and receiving care in their work. They also support the main CCP team, and have provided feedback on the data collection tools to ensure they are accessible. They will continue to advise the project team on the accessibility of data collection tools and outputs related to the project. We thank the advisors for their input to the evaluation.

ANALYSIS AND SYNTHESIS OF THE DATA

The aim of the analysis is to provide evidence about whether RPPs are a promising approach for driving improvements in practice in the care home context and to understand how, why and in what circumstances RPPs contribute to enhancing research and research use in local care homes and informing wider care home improvement efforts. Since this is a longitudinal evaluation, data will be gathered in waves and analysis will proceed iteratively, using evidence gathered from previous waves to inform subsequent data collection. Following each data collection wave findings will be updated to generate a picture of how the CCP partnerships are developing over time, and the ToC/programme theory refined as we learn more about how and why the CCP partnerships are working and the kinds of impact they are having. At each wave the available data will be analysed in stages.

For peer review only - http://bmjopen.bmj.com/site/about/guidelines.xhtml

The first stage is to prepare descriptive profiles for each site. Each dataset will be analysed independently initially. We will use framework analysis [58], supported by Nvivo software to index the qualitative data (interviews, observation, document analysis) and identify evidence for outcomes, outputs, key constructs (e.g. boundary infrastructure), activities or strategies being enacted by the partnership. To inform decisions about whether or not data can be considered as evidence for or against outcomes, outputs and key constructs we will draw on theory and studies of research use [47,59]. Working within-case study sites, we will then compare across data types to triangulate evidence for each outcome, output and activity in a first stage of synthesis. This will enable us to develop outcome, output and activity profiles for each site, which will be used for the economic analysis.

Subsequently analysis will focus on the sub-questions, working first within case study sites then comparing across case study sites. The outcomes profile will enable us to assess sub-question 1a -- the extent to which outcomes have been achieved by each RPP. To address sub-question 1b and determine how significant a contribution the CCP partnership is making to the observed outcomes, we will use contribution analysis. We will follow the analytical steps outlined by Mayne and practical guidance [40–42] to use the evidence we gather to assemble and assess the contribution stories for how the partnerships have led to research being produced that is used to improve practice within the site and care improvements beyond the site. An important part of this analysis will be to understand the influence of the CCP co-design and implementation support teams. Comparing across case studies to identify whether patterns are consistent or are specific to particular CCP partnership will be important for ToC refinement.

We will complement our use of contribution analysis by drawing on realist methods to explore in more depth how, why and the circumstances in which the CCP partnerships contribute to each outcome (sub-question 1c). The focus will be on developing and refining links between CMOs, following guidance for realist evaluation [37], as well as exploring narrower aspects of causality within the broader ToC [60]. As the analysis progresses, we will explore how later CMOs relate to and might depend on earlier CMOs [61]. We will also investigate whether these patterns occur regardless of context, or are specific to particular CCP partnerships by comparing across sites. This analysis will provide insight, for example, into whether certain strategies are more suited to particular contexts.

Finally, we will explore whether the way in which the CCP partnerships are operating is consistent with the RPP approach (sub-question d). Additional coding schemes will be developed to capture who is involved in the activities, their context and purpose, the way in which they are being enacted (e.g. power differentials are present and not addressed), their consequences, and the contextual factors influencing the initiation and progress of the activities/strategies. As coding proceeds, the team will write memos to capture thinking around whether activities/strategies can be considered as faithful to the RPP approach, the applicability of the RPP approach to the social care context and what these new partnerships can tell us about whether the core principles underpinning RPPs need to be adapted.

For peer review only - http://bmjopen.bmj.com/site/about/guidelines.xhtml

ECONOMIC EVALUATION

The economic evaluation will focus on exploring some of the more tangible economic consequences and utilise knowledge on indicators to model economic consequences for different types of outcomes. The analytical objectives for this stream are to establish the costs and economic consequences of RPPs, which combined will be used to derive an understanding of economic value of the RPP approach. The economic evaluation aligns with the theory-based evaluation and will draw on the data collection and analysis, using in particular the activity, output and outcomes profiles.

Full cost-effectiveness analysis would not be appropriate. Instead, we will use a 'narrative' economic analysis to examine both the costs of delivering the RPPs and some of the potential economic consequences. This method, widely used in the social care context draws on simulation modelling and cost-consequence analysis techniques [62–64]. It provides information on the estimated costs of an initiative and the estimated cost of alternatives enabling the decision maker to determine whether a course of action is worth investing in given the particular context in which they operate.

There are two parts to the analysis: part one, assesses the costs of delivering RPPs, and part two, models the economic consequences of RPPs. The two parts are subsequently synthesised to assess the value for money for each of the RPPs. We will take a health and social care and broader societal value perspective taken in the economic analysis. The latter will consider improvements in (health- or social care-related) quality of life, productivity and unpaid care.

Assessing costs

To cost the RRPs, we will use both bottom-up and top-down approaches [65]. Unit costs will be attached to each activity in the activity profile, using local sources where possible or – where this is not possible – adapted from national sources to reflect local salaries, overheads and capital costs. Budget information will be used where it is not possible to obtain bottom-up data on activities and for other resource use. Descriptive costs profiles will be developed for each site.

Modelling economic consequences

Potential economic consequences will be established through the ToC development and refinement process. As a first step, this will therefore include the further development of the outputs and outcomes profiles, to derive economic indicators, and expected trajectories to potential economic impacts. From this, economic vignettes will be drawn for each site.

In a next step, monetary values will be assigned to outputs and outcomes identified in the vignettes as being linked to economic impacts. For some of the economic impacts it will be possible to attach monetary values either directly, or based on data

For peer review only - http://bmjopen.bmj.com/site/about/guidelines.xhtml

from published sources (through modelling). An example of a consequence with direct monetary value is the income gained from a joint grant activity. An example of a consequence that would require further modelling to assign a monetary value is the implementation of an evidence-based intervention as part of service and quality improvements known to be cost-effective (such as the implementation of cognitive stimulation-therapy for people with dementia [66]). Modelling will use (where available) local data or information from the sites, and published data.

Since some of the economic gains will be realised during the research period whilst others will take place in the future, the analysis will have different time horizons (e.g. short-, medium-, long-term) reflecting differences in the certainty of (potential) economic gains. For example, it may be the case that a research project completed during the study period with known economic consequences for the care homes, but in another site a research project may only just have started or may still be at the planning stage, but nevertheless with expected but uncertain future economic consequences.

Cross-site comparison and synthesising costs and economic consequences

As economic consequences are likely to differ across sites, we need a way of structuring and categorising them to facilitate a narrative comparison between RPPs. Our starting point it to use the 'Payback Framework', which has been developed for examining the impact of health research [67]. It offers a multi-dimensional categorisation of benefits ranging from more traditional academic benefits of knowledge production to wider benefits to society, but it may need some adaptation to this context.

ETHICS AND DISSEMINATION

The study has undergone ethics review by the HRA Research Ethics Committee (REC) and has been reviewed in accordance with the London School of Economics (LSE) Research Ethics Policy and Procedure. The study does not pose major ethical issues or chance of harm for participants. Key issues relate to the observational component of the research and working with care home providers and ensuring steps to maintain confidentiality.

Processes for consent

Although the partnerships are obliged to participate in the evaluation, participation of individuals is voluntary. We will obtain written informed consent from all partnership members and stakeholders for all research activities that they will participate in. Consent will be obtained at the start of the research and again at the start of each research activity, with participants able to withdraw at any time.

For peer review only - http://bmjopen.bmj.com/site/about/guidelines.xhtml

Consent for observations of events and other activities related to the partnerships that involve people who are not closely connected to the partnership (and therefore have not previously given consent to be involved in research activities) will be achieved through negotiated and privileged access to the field and implied consent. An information sheet will be sent to participants in advance with the papers for events and a script prepared for the event Chair to introduce the researchers. Participants will have an opportunity to raise concerns at the start of any event and refuse permission for observation. Discussion of the CCP evaluation aims and objectives with the partnerships at the outset of their work and through personal conversations with the local evaluators will contribute to raising awareness and enabling implied consent.

Data Management and anonymisation

Data will be stored and managed in accordance with university and national rules and regulations as described in the project data management plan. Steps will be taken to minimise any risk of breaching confidentiality of research or personal data. Any personal information that could identify participants (such as name or job title) will be removed or changed before results are made public. All data collected from the activity diary and the survey will be reported at an appropriate level of aggregation so individuals cannot be identified.

Dissemination

Outputs will include interim case study reports and a comparative report for each analysis phase. The economic analysis will be conducted towards the end of the evaluation timeframe so will be included in the final report. An economic framework will be produced that can be used by those who want to replicate the analyses of economic value of RPPs.

The final analysis and synthesis will be published as a final report and articles covering the different aspects of the evaluation will be submitted to international journals. The final report will feed into three workshops to be held at the end of the project. These workshops will focus on the sustainability and spread of the RPP approach beyond the CCP sites to the rest of the UK, and sharing leaning from the study with interest groups, thought leaders, and senior policymakers in social care from across the UK.

AUTHORS' CONTRIBUTION

All authors contributed to conceptualisation of the study, with leadership from ABoaz for the CCP project, JM for the evaluation and ABauer for the economic evaluation. JM wrote the first draft with inputs from HK and ABauer. MK and ABoaz critically reviewed the manuscript and provided comments to improve it. All authors have read and approved the final manuscript.

For peer review only - http://bmjopen.bmj.com/site/about/guidelines.xhtml

COMPETING INTERESTS STATEMENT

The authors have no competing interests to declare.

FUNDING STATEMENT

This work was supported by the National Institute for Health and Care Research (NIHR) under the Health and Social Care Delivery research programme, grant number NIHR131335. The views expressed are those of the authors and not necessarily those of the NIHR or the Department of Health and Social Care.

For peer review only

For peer review only - http://bmjopen.bmj.com/site/about/guidelines.xhtml

REFERENCES

1 Knapp M, Manthorpe J, Mehta A, *et al.* Developing the evidence base for adult social care practice: The NIHR School for Social Care Research. *J Care Serv Manag* 2010;**4**:167–79. doi:10.1179/175016810x12670238442101

2 Ghate D, Hood R. Using evidence in social care. In: Boaz A, Davies H, Fraser A, *et al.*, eds. *What works now? : evidence-informed policy and practice*. Bristol, UK: : Policy Press 2019. 89–109.

3 Metz A, Boaz A, Robert G. Co-creative approaches to knowledge production: What next for bridging the research to practice gap? *Evidence and Policy* 2019;**15**:331–7. doi:10.1332/174426419X15623193264226

4 Davies SL, Goodman C, Manthorpe J, *et al.* Enabling research in care homes: an evaluation of a national network of research ready care homes. *BMC Medical Research Methodology 2014 14:1* 2014;**14**:1–8. doi:10.1186/1471-2288-14-47

5 Walter I, Nutley S, Percy-Smith J, *et al.* Improving the use of research in social care practice. SCIE Knowledge Review 7. 2004.

6 Boaz A, Bauer A, Fitzsimons B, *et al.* Do Research Practice Partnerships offer a promising approach to knowledge production? . *Journal of Long-Term Care*

7 Penuel WR, Hill HC. Building a Knowledge Base on Research-Practice Partnerships: Introduction to the Special Topic Collection. *AERA Open* 2019;**5**:233285841989195. doi:10.1177/2332858419891950

8 Farrell C, Penual W, Coburn C, *et al.* Research-Practice Partnerships in Education: The State of the Field. 2021.

9 Arce-Trigatti P, Farrell C. Research–Practice Partnerships in Education Within The United States. *Education (Chula Vista)* Published Online First: 21 April 2021. doi:10.1093/OBO/9780199756810-0274

10 Henrick E, Cobb P, Penuel W, *et al.* Assessing Research-Practice Partnerships Five Dimensions of Effectiveness. New York, NY: 2017.

11 Booker L, Conaway C, Schwartz N. Five Ways RPPs Can Fail and How to Avoid Them: Applying Conceptual Frameworks to Improve RPPs. New York, NY: 2019.

12 Connolly F. Measuring the Value of a Research-Practice Partnership. *NNERP Extra* 2019;**1**:6–9.http://nnerppextra.rice.edu/measuring-the-value-of-an-rpp/ (accessed 24 Apr 2022).

13 Skivington K, Matthews L, Simpson SA, *et al.* A new framework for developing and evaluating complex interventions: update of Medical Research Council guidance. *BMJ* 2021;**374**. doi:10.1136/BMJ.N2061

14 Penuel W, Soland J, Farrell CC, *et al.* Assessing the outcomes of Research-Practice Partnerships: An Evaluation Argument. 2021.

For peer review only - http://bmjopen.bmj.com/site/about/guidelines.xhtml

15 Tseng V. The Next Big Leap for Research-Practice Partnerships: Building and Testing Theories to Improve Research Use. New York: 2017. http://wtgrantfoundation.org/library/uploads/2017/12/Next-Big-Leap_Vivian-Tseng_WTG_2017.pdf (accessed 24 Apr 2022).

16 Penuel W, Furtak EM, Farrell C. Research-practice partnerships in education. Advancing an evolutionary logic of systems improvement. *Die deutsche Schule* 2021;**113**:45–62.https://www.pedocs.de/volltexte/2021/22074/pdf/DDS_2021_1_Penuel_et_al_Research-Practice_Partnerships.pdf (accessed 24 Apr 2022).

17 Penuel W, Allen AR, Coburn C, *et al.* Conceptualizing Research–Practice Partnerships as Joint Work at Boundaries. *J Educ Stud Placed Risk* 2015;**20**:182–97. doi:10.1080/10824669.2014.988334

18 Farrell C, Penuel W, Allen A, *et al.* Learning at the Boundaries of Research and Practice: A Framework for Understanding Research–Practice Partnerships. *Educational Researcher* 2022;**51**:197–208. doi:10.3102/0013189X211069073

19 Farrell C, Coburn C. Absorptive capacity: A conceptual framework for understanding district central office learning. *Journal of Educational Change 2016 18:2* 2016;**18**:135–59. doi:10.1007/S10833-016-9291-7

20 Farrell CC, Harrison C, Coburn CE. “What the Hell Is This, and Who the Hell Are You?” Role and Identity Negotiation in Research-Practice Partnerships. *AERA Open* 2019;**5**:233285841984959. doi:10.1177/2332858419849595

21 Denner J, Bean S, Campe S, *et al.* Negotiating Trust, Power, and Culture in a Research–Practice Partnership: *https://doi.org/101177/2332858419858635* 2019;**5**:233285841985863. doi:10.1177/2332858419858635

22 Henrick E, McGee S, Penuel W. Attending to Issues of Equity in Evaluating RPP Outcomes. *NNERPP Extra* 2019;**1**:8–13.http://nnerppextra.rice.edu/attending-to-issues-of-equity-in-evaluating-rpps/ (accessed 4 Jun 2022).

23 Stanton MC, Ali SB, The SUSTAIN Center Team. A typology of power in implementation: Building on the exploration, preparation, implementation, sustainment (EPIS) framework to advance mental health and HIV health equity: *Implement Res Pract* 2022;**3**:263348952110642. doi:10.1177/26334895211064250

24 Kreindler S, Dowd D, Star N, *et al.* Silos and Social Identity: The Social Identity Approach as a Framework for Understanding and Overcoming Divisions in Health Care. *Milbank Q* 2012;**90**:347–74.

25 Hawe P, Shiell A, Riley T. Theorising interventions as events in systems. In: *American Journal of Community Psychology*. 2009. 267–76. doi:10.1007/s10464-009-9229-9

26 Farrell CC, Penuel WR, Allen A, *et al.* Learning at the Boundaries of Research and Practice: A Framework for Understanding Research–Practice Partnerships. *Educational Researcher* 2022;:0013189X211069073.

For peer review only - http://bmjopen.bmj.com/site/about/guidelines.xhtml

27 Blamey A, Mackenzie M. Theories of Change and Realistic Evaluation: Peas in a Pod or Apples and Oranges? *Evaluation* 2007;**13**:439–55. doi:10.1177/1356389007082129

28 Weiss C. Theory-based evaluation: past, present and future. *New Dir Eval* 1997;**76**:41–55. doi:10.1002/ev.1086

29 Mayne J. Contribution analysis: Coming of age?: *http://dx.doi.org/101177/1356389012451663* 2012;**18**:270–80. doi:10.1177/1356389012451663

30 Brousselle A, Buregeya JM. Theory-based evaluations: Framing the existence of a new theory in evaluation and the rise of the 5th generation: *https://doi.org/101177/1356389018765487* 2018;**24**:153–68. doi:10.1177/1356389018765487

31 Pawson R, Tilley N. *Realistic evaluation*. Sage Publications 1997.

32 Amon E, Hill S, Blake J, *et al.* Brokering Community-Based Research. *Mich J Community Serv Learn* 2020;**26**. doi:https://doi.org/10.3998/mjcsloa.3239521.0026.107

33 Mayne J. Theory of Change Analysis: Building Robust Theories of Change. *Canadian Journal of Program Evaluation* 2017;**32**:155–73. doi:10.3138/CJPE.31122

34 Morton S. Progressing research impact assessment: A ‘contributions’ approach. *Res Eval* 2015;**24**:405–19. doi:10.1093/RESEVAL/RVV016

35 Pawson R. Evidence-based policy: A realist perspective. In: *Making Realism Work: Realist Social Theory and Empirical Research*. 2004. doi:10.4324/9780203624289

36 Dalkin S, Lhussier M, Williams L, *et al.* Exploring the use of Soft Systems Methodology with realist approaches: A novel way to map programme complexity and develop and refine programme theory: *https://doi-org.gate3.library.lse.ac.uk/101177/1356389017749036* 2018;**24**:84–97. doi:10.1177/1356389017749036

37 Wong G, Westhorp G, Greenhalgh J, *et al.* Quality and reporting standards, resources, training materials and information for realist evaluation: the RAMESES II project. *Health Services and Delivery Research* 2017;**5**:1–108. doi:10.3310/HSDR05280

38 Jagosh J. Realist Synthesis for Public Health: Building an Ontologically Deep Understanding of How Programs Work, For Whom, and In Which Contexts. *Annu Rev Public Health* 2019;**40**:361–72. doi:10.1146/ANNUREV-PUBLHEALTH-031816-044451

39 Rycroft-Malone J, McCormack B, Hutchinson AM, *et al.* Realist synthesis: illustrating the method for implementation research. *Implement Sci* 2012;**7**. doi:10.1186/1748-5908-7-33

For peer review only

For peer review only - http://bmjopen.bmj.com/site/about/guidelines.xhtml

40 Delahais T, Toulemonde J. Applying contribution analysis: Lessons from five years of practice: *http://dx.doi.org/101177/1356389012450810* 2012;**18**:281–93. doi:10.1177/1356389012450810

41 Lemire ST, Nielsen SB, Dybdal L. Making contribution analysis work: A practical framework for handling influencing factors and alternative explanations. *Evaluation* 2012;**18**:294–309. doi:10.1177/1356389012450654

42 Mayne J. Contribution Analysis: Addressing Cause and Effect. In: Marra M, ed. *Evaluating the Complex*. New York: : Routledge 2011. 65–108. doi:10.4324/9781315147406-8/CONTRIBUTION-ANALYSIS-ADDRESSING-CAUSE-E

43 Barbrook-Johnson P, Penn A. Participatory systems mapping for complex energy policy evaluation: *https://doi.org/101177/1356389020976153* 2021;**27**:57–79. doi:10.1177/1356389020976153

44 Chazdon S, Emery M, Hansen D, *et al. A Field Guide to Ripple Effects Mapping*. University of Minnesota Libraries Publishing 2017. http://conservancy.umn.edu/handle/11299/190639 (accessed 17 Sep 2022).

45 Yin RK. *Case study research and applications —Design and methods*. 6th ed. Newbury Pk, CA: : Sage 2018.

46 Gitomer DH, Crouse K. Studying the Use of Research Evidence: A Review of Methods. 2019.

47 Farrell C, Davidson K, Repko-Erwin M, *et al.* A Descriptive Study of the IES Researcher-Practitioner Partnerships in Education Research Program: Final Report. Technical Report No. 3. National Center for Research in Policy and Practice. Web site: http://ncrpp.org/ 2018. www.ncrpp.org, (accessed 29 Sep 2021).

48 Penuel W, Briggs D, Davidson K, *et al.* Survey of practitioner's use of research. 2016. https://osf.io/84hbu/ (accessed 1 Jun 2022).

49 Watson B, Clarke C, Swallow V, *et al.* Exploratory factor analysis of the research and development culture index among qualified nurses. *J Clin Nurs* 2005;**14**:1042–7. doi:10.1111/J.1365-2702.2005.01214.X

50 Francis-Smythe J, Haase S, Steele C, *et al.* Competencies and continuing professional development (CPD) for academics in knowledge exchange (KE) activity. 2006. https://eprints.worc.ac.uk/id/eprint/667 (accessed 17 Sep 2022).

51 Mael F, Ashforth BE. Alumni and their alma mater: A partial test of the reformulated model of organizational identification. *J Organ Behav* 1992;**13**:103–23. doi:10.1002/JOB.4030130202

52 Meyer JP, Allen NJ. A three-component conceptualization of organizational commitment. *Human Resource Management Review* 1991;**1**:61–89. doi:10.1016/1053-4822(91)90011-Z

For peer review only - http://bmjopen.bmj.com/site/about/guidelines.xhtml

53 Conway JM, Lance CE. What Reviewers Should Expect from Authors Regarding Common Method Bias in Organizational Research. *Journal of Business and Psychology 2010 25:3* 2010;**25**:325–34. doi:10.1007/S10869-010-9181-6

54 Howard GS, Dailey PR, Gulanick NA. The Feasibility of Informed Pretests in Attenuating Response-Shift Bias: *http://dx.doi.org/101177/014662167900300406* 2016;**3**:481–94. doi:10.1177/014662167900300406

55 Skivington K, Matthews L, Simpson SA, *et al.* A new framework for developing and evaluating complex interventions: Update of Medical Research Council guidance. *The BMJ* 2021;**374**. doi:10.1136/bmj.n2061

56 Schwandt TA, Gates EF. *Evaluating and valuing in social research*. New York: : Guilford Press 2021. https://www.guilford.com/books/Evaluating-and-Valuing-in-Social-Research/Schwandt-Gates/9781462547326 (accessed 4 Jun 2022).

57 Gates EF. Toward Valuing With Critical Systems Heuristics: *https://doi.org/101177/1098214017703703* 2017;**39**:201–20. doi:10.1177/1098214017703703

58 Ritchie J, Spencer L. Qualitative data analysis for applied policy research. In: Huberman AM, Miles MB, eds. *The qualitative researcher's companion*. Sage Publications 2002. 410.https://books.google.co.uk/books?hl=en&lr=&id=46jfwR6y5joC&oi=fnd&pg=PA305&dq=ritchie+spencer+framework+analysis&ots=soEWJQuuQR&sig=L5CSWX3hHT8XyZGhkbxdBF3JLgc#v=onepage&q=ritchie%20spencer%20framework%20analysis&f=false (accessed 6 Jun 2019).

59 Honig MI, Venkateswaran N, McNeil P. Research Use as Learning: The Case of Fundamental Change in School District Central Offices: *https://doi.org/103102/0002831217712466* 2017;**54**:938–71. doi:10.3102/0002831217712466

60 Rolfe S. Combining Theories of Change and Realist Evaluation in practice: Lessons from a research on evaluation study: *Evaluation* 2019;**25**:294–316. doi:10.1177/1356389019835229

61 Jagosh J, Bush PL, Salsberg J, *et al.* A realist evaluation of community-based participatory research: Partnership synergy, trust building and related ripple effects. *BMC Public Health* 2015;**15**:1–11. doi:10.1186/S12889-015-1949-1/TABLES/2

62 Knapp M, Bauer A, Perkins M, *et al.* Building community capital in social care: is there an economic case? *Community Dev J* 2013;**48**:313–31. doi:10.1093/CDJ/BSS021

63 Bauer A, Wistow G, Dixon J, *et al.* Investing in advocacy for parents with learning disabilities: what is the economic argument? *Br J Learn Disabil* 2015;**43**:66–74. doi:10.1111/BLD.12089

64 Bauer A, Taggart L, Rasmussen J, *et al.* Access to health care for older people with intellectual disability: A modelling study to explore the cost-effectiveness of

For peer review only - http://bmjopen.bmj.com/site/about/guidelines.xhtml

health checks. *BMC Public Health* 2019;**19**:1–16. doi:10.1186/S12889-019-6912-0/TABLES/7

65 Beecham J. Unit Costs-Not Exactly Child's Play. A guide to estimating unit costs for children's social care. Canterbury, Kent: 2000. https://www.pssru.ac.uk/pub/B062.pdf (accessed 4 Jun 2022).

66 D'Amico F, Rehill A, Knapp M, *et al.* Maintenance Cognitive Stimulation Therapy: An Economic Evaluation Within a Randomized Controlled Trial. *J Am Med Dir Assoc* 2015;**16**:63–70. doi:10.1016/J.JAMDA.2014.10.020

67 Donovan C, Hanney S. The 'Payback Framework' explained. *Res Eval* 2011;**20**:181–3. doi:10.3152/095820211X13118583635756

For peer review only

For peer review only - http://bmjopen.bmj.com/site/about/guidelines.xhtml

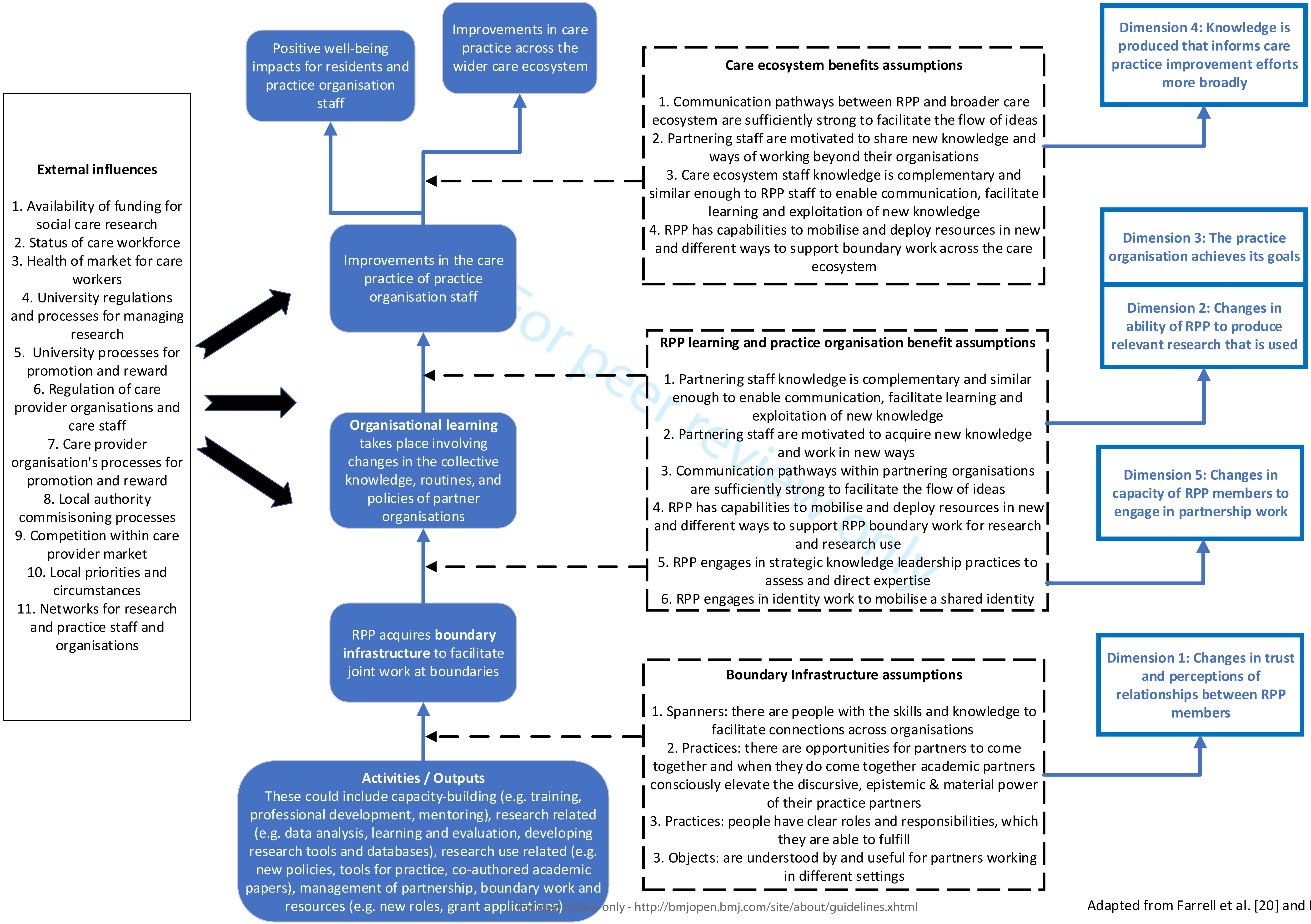

Adapted from Farrell et al. [20] and Henrick et al [10]

BMJ Open

A theory-based evaluation of three Research Practice Partnerships designed to deliver novel, sustainable collaborations between adult social care research and practice in the UK: a research protocol for a 'layered' contributions analysis and realist evaluation

Journal:	*BMJ Open*
Manuscript ID	bmjopen-2022-068651.R1
Article Type:	Protocol
Date Submitted by the Author:	28-Oct-2022
Complete List of Authors:	Malley, Juliette; The London School of Economics and Political Science, Care Policy and Evaluation Centre<br>Bauer, Annette; London School of Economics and Political Science, Care Policy and Evaluation Centre<br>Boaz, Annette; London School of Hygiene & Tropical Medicine<br>Kendrick, Hannah; London School of Economics and Political Science, Care Policy and Evaluation Centre<br>Knapp, Martin; The London School of Economics and Political Science, Care Policy and Evaluation Centre
**Primary Subject Heading**:	Health services research
Secondary Subject Heading:	Evidence based practice, Research methods
Keywords:	Health policy < HEALTH SERVICES ADMINISTRATION & MANAGEMENT, Organisational development < HEALTH SERVICES ADMINISTRATION & MANAGEMENT, Quality in health care < HEALTH SERVICES ADMINISTRATION & MANAGEMENT

SCHOLARONE™
Manuscripts

Full title: A theory-based evaluation of three Research Practice Partnerships designed to deliver novel, sustainable collaborations between adult social care research and practice in the UK: a research protocol for a 'layered' contributions analysis and realist evaluation

Short title: Creating Care Partnerships Evaluation

Juliette Malley[1], Annette Bauer[2], Annette Boaz[3], Hannah Kendrick[4], Martin Knapp[5]

Corresponding Author:

[1] Juliette Malley j.n.malley@lse.ac.uk; Care Policy and Evaluation Centre (CPEC), London School of Economics and Political Science, Houghton Street, London WC2A 2AE, UK

Co-authors:

[2] Annette Bauer a.bauer@lse.ac.uk; Care Policy and Evaluation Centre (CPEC), London School of Economics and Political Science, Houghton Street, London WC2A 2AE, UK

[3] Annette Boaz annette.boaz@lshtm.ac.uk; London School of Hygiene and Tropical Medicine, 15-17 Tavistock Place, London WC1H 9SH, UK

[4] Hannah Kendrick h.kendrick@lse.ac.uk; Care Policy and Evaluation Centre (CPEC), London School of Economics and Political Science, Houghton Street, London WC2A 2AE, UK

[5] Martin Knapp M.Knapp@lse.ac.uk; Care Policy and Evaluation Centre (CPEC), London School of Economics and Political Science, Houghton Street, London WC2A 2AE, UK

KEYWORDS

care homes; research-practice partnerships; theory-based evaluation; economic evaluation

Word count: 5,687 words

For peer review only

For peer review only - http://bmjopen.bmj.com/site/about/guidelines.xhtml

ABSTRACT

Introduction

Research practice partnerships (RPPs) are long-term collaborations between research and practice that aim to conduct research that can be used to make practice-based improvements. They intentionally bring together diverse experience in decision making and seek to shift power dynamics so that all partners have a say. The Creating Care Partnerships project aims to explore whether the RPP approach developed within the US educational context can be successfully applied to the English care home context. The project involves a programme of co-design, implementation, and evaluation within three case study sites. This protocol set outs the aims, research design, and governance of the evaluation.

Methods and analysis

The evaluation takes a theory-based approach to explore how, why and in what circumstances RPPs in the care home context contribute to enhancing research and research use in local care homes and informing wider improvement efforts. A mixed methods design will be used for each case study, including semi-structured interviews, observations of RPP events and meetings, an online survey, activity diary and review of local data and documents. Data collection will proceed in waves, with the theory of change (ToC) being continually refined and used to guide further data collection and analysis. Insights will be drawn using Contribution Analysis, Realist Evaluation, and systems perspectives to assess the contribution made by the case study sites to achieving outcomes and the influence of contextual factors. Economic consequences will be identified through the ToC, using a narrative economic analysis to assess costs, consequences, and value for money.

Ethics and dissemination

The study has undergone ethics review by HRA Research Ethics Committee. It does not pose major ethical issues. A final report will be published and articles will be submitted to international journals.

STRENGTHS AND LIMITATIONS OF THIS STUDY

- A theory-based approach allows for greater explanation of how RRPs work, whilst appreciating the complexity and non-linearity of implementation.
- The use of mixed methods allows us to draw on the strengths of different methods, improving the credibility of evaluation findings.
- An economic evaluation will allow policymakers and funders to make evidence-based decisions about the value of further investment in RPPs for the care home sector.

For peer review only - http://bmjopen.bmj.com/site/about/guidelines.xhtml

- The evaluation period may not be long enough to capture the extent to which outcomes have been achieved within either local care homes or the wider care ecosystem.
- Theory-based approaches, including contributions analysis and realist evaluation that are used here, are not as well-established as other approaches for establishing effectiveness. This study may draw criticism for not being as rigorous as experimental approaches.

For peer review only

For peer review only - http://bmjopen.bmj.com/site/about/guidelines.xhtml

INTRODUCTION

An important question for adult social care (ASC) is how investment in research can be optimised to support improvements for people drawing on social care services. Despite significant investment and a growing evidence base [1], the evidence produced is in general poorly used by the ASC practice community (e.g. social care commissioners, providers and the workforce) [2]. There is a growing interest in approaches that focus on building relationships and stronger links between those who produce research, those who use research and the intended beneficiaries to complement and augment existing efforts and infrastructure investments [3–5]. The Creating Care Partnerships (CCP) project aims to re-design for the care home context and implement a promising approach called Research Practice Partnerships (RPPs) in three sites across England [6]. Reflecting the central place learning has in the CCP project, it also includes an evaluation of the new RPPs. This paper describes the evaluation protocol.

RPPs are a specific form of partnership that offer a different way of producing and mobilising research that fundamentally challenges the status quo. They are long-term collaborations between research and practice communities that aim to bring about real-world change through the use of research evidence. RPPs can vary in scope and size and may have different strengths, but not all research-practice collaborations are RPPs [7,8]. To be an RPP, collaborative efforts must extend beyond the life of a single research study or project and must engage with research as a core activity. Another feature of RPPs is the intentional integration of expertise from two communities -- practice and research – that are often disconnected. Relatedly RPPs engage in activities to shift power relations to ensure everyone has a say in the research endeavours; people from practice communities are involved from the outset, and both communities contribute equitably to shaping the direction of the work and supporting the use of what is learned from the research [8]. Although a substantial corpus of research has developed that describes the core principles of an RPP with lessons for those seeking to reproduce it (see NNERPP RPP knowledge clearinghouse https://nnerpp.rice.edu/rpp-knowledge-clearinghouse/, William T. Grant microsite https://rpp.wtgrantfoundation.org/), questions remain around what effective partnering looks like [8,9]. How well RPPs meet their goals and the conditions that support or hinder their progress are seen as pressing issues for research [8,10,11].

The main question for the CCP evaluation is whether this approach, which has been developed within the US educational context, can be successfully adapted for and implemented within the English care home context with similarly positive results in terms of driving improvements in practice and in the wellbeing of recipients of social care services. The primary aim of the CCP evaluation is therefore to provide evidence about the effectiveness of RPPs in the care home context; but, with a view to ensuring a legacy from the project, a second aim is to gather evidence about how to implement and sustain the approach so it can be reproduced elsewhere. The English care home context is very different to the US education context – a key difference being the lack of professionalisation and lower educational attainment of the majority of the care home workforce compared to educators, but there are also differences in the higher education contexts between the two countries and the

For peer review only - http://bmjopen.bmj.com/site/about/guidelines.xhtml

research and innovation infrastructure. We expect this evaluation to deepen understanding of the way in which local conditions affect how RPPs function, the kinds of strategies they need to leverage to enact the RPP guiding principles and, possibly, what RPPs look like, with lessons for the international RPP community. Given the economic context and existing investments in research and knowledge mobilisation, a third aim of the evaluation is to understand the desirability of further investment in RPPs given the costs and the value that flows from the investment. Since the question of the economic value of RPPs is only beginning to be considered [12], this element of the evaluation is novel and will contribute to developing schemas for assessing value.

The CCP co-design work has produced a set of guiding principles for RPPs operating in the care home context that will be operationalised in different ways by each new RPP. Reflecting the strongly theoretical and complex nature of RPPs, our evaluation perspective is theory-based and draws on a systems perspective [13]. It addresses the following questions (and sub-questions):

1. How, why and in what circumstances do RPPs in the care home context contribute to enhancing research and research use in local care homes and informing wider care home improvement efforts?
    a. To what extent have the main outcomes been achieved?
    b. How significant is the contribution of the CCP partnership to the main outcomes, given other factors?
    c. How, why and in what circumstances do the CCP partnerships contribute to each outcome?
    d. To what extent is the way the CCP partnerships operate consistent with the RPP approach?
2. What are the costs of delivering RPPs in the care home context, and are they good value for money?

It is not yet standard practice to publish protocols for evaluations of the kind outlined here. Our intention in publishing this protocol is to increase transparency in our methods and encourage discussion around them.

THEORETICAL FRAMEWORK

We use the evidence-based framework developed by Henrick and colleagues [10] and adapted to the care home context as a framework for the evaluation. It identifies five dimensions of outcomes for successful RPPs: (1) building trust and cultivating partner relationships, (2) producing relevant research that is used, (3) supporting the practice organization in achieving its goals, (4) producing knowledge that can inform social care practice improvements more broadly, and (5) building the capacity of participants to engage in the partnership work [9]. This framework provides a focus for measurement of RPP effectiveness and the integration of findings across the sites, but it can also inform the development and sustainability of such partnerships and theories of change [14–16].

A key strength of the framework is that it is flexible enough to allow multiple theories to inform the evaluation, which existing research indicates will be necessary to understand how and why RPPs work. RPPs have been conceptualised in a variety of

For peer review only - http://bmjopen.bmj.com/site/about/guidelines.xhtml

ways [11,15], but there is a consensus around the notion of RPPs as engaged in joint work at boundaries [17,18]. In addition to theories of boundary infrastructure (including boundary spanners, practices and objects), scholars have drawn on organisational theories (including absorptive capacity, organisational learning and organisational routines) to explain how RPPs successfully produce and use research that improves practice within the practice organisation and has wider sectoral impacts [17–19]. Since working across boundaries is challenging [11], many of the conceptual contributions also focus on relational aspects, including building trust, redistributing power, conflict and consensus, identity and role negotiation, and leadership [18,20–22]. Theoretical contributions that help to analyse different types of power and how they are distributed [23] and how new identities are formed or resisted [24] may prove useful.

Evidence from existing RPPs suggests that it takes time for them become productive and embedded within the wider higher education and practice ecosystems [14]. As RPPs start to have an impact on and beyond the partnering organisations it may be useful to conceptualise them as 'events in systems' [25]. This perspective draws attention to the fact that RPPs are not neutral additions to the ecosystem but through their intention to have broader impact on care practice and how research is done, they challenge the status quo and in complex ways will interact with, shape and be shaped by these wider contexts. Understanding how the RPP and other players in the wider ecosystem (e.g. higher education institutions, funding bodies, local authorities) interact with each other over time is key to understanding the potential for sustainability and spread of RPPs, as to endure RPPs must become resilient to shocks from within (e.g. organisational turnover, conflicts, and competing organisational norms) and, crucially, shocks from outwith the partnership (e.g. changes in policy, economic shocks, changes in funding) [26].

DESIGN AND METHODS FOR THE THEORY-BASED EVALUATION

The evaluation is theory-based and employs a multiple case study design with longitudinal data collection. There are various approaches to theory-based evaluation, which differ in their methods for constructing a valid ToC or programme theory and the types of questions for which they are designed to respond [27–31]. In this evaluation, we use contribution analysis, and realist evaluation sequentially as 'layered tactics' to address different sub-questions [32].

The contribution analysis lens addresses whether the RPPs make a meaningful contribution to enhancing research and research use in care homes and the wider system (1b), by taking into account other factors and rival explanations [29,33,34]. It produces credible causal claims about the contribution RPPs make to observed outcomes allowing us to draw conclusions about whether RPPs are a promising approach in the English care home context. By contrast through realist methods, we can probe in greater depth the different ways in which the CCP partnerships may implement the RPP approach, the circumstances that may affect the choices they make and the outcomes observed (1c). Realist evaluation explores causality through developing and testing programme theories as context-mechanism-outcome (CMO)

For peer review only - http://bmjopen.bmj.com/site/about/guidelines.xhtml

configurations, which explain how and why RPPs might trigger different change mechanisms across different contexts to achieve (or not achieve) outcomes [31,35]. The three partnerships -- Research and Practice Development Care Partnership (RPDCP) in north-west England, Care and Research North East, and Lancashire partnership -- were selected through an open competition to offer contrasting situations. They differ in the size, scope, types of partners and local context, which will allow us to explore conducive or inhibiting contexts for RPPs [36]. Should it prove useful for mapping the complexity of the RPPs' context and refining our ToC (e.g. because the RPPs are actively seeking to have impact beyond their sites) we will add a third soft systems lens [36].

Stage 1: Development of the initial theory of change and hypotheses

For theory-based evaluations, the theory of change (ToC) or programme theory plays a central role in assuring the quality of the evaluation. It guides the measurement of concepts and the investigation of causal relationships between the activities of RPPs, outputs and outcomes. There is guidance for developing and testing ToC/programme theory for contributions analysis and realist evaluation that we will follow [33,37–42]. The ToC and hypotheses about CMO combinations will be informed by the literature on RPPs in the US education context, similar partnerships between research and practice in ASC and related fields, and insights about how the approach might translate to the English care home context gathered through the co-design work conducted as part of the first stage of the CCP project.

Based on an initial review, Figure 1 sets out a ToC for RPPs in the care home context. In setting out the theoretical causal chain through which activities/outputs lead to improvements in care practice in the care homes and the wider care ecosystem and the assumptions that need to be met for this chain of events to come about, we draw heavily on Farrell et al.'s work [18]. Influencing factors are drawn from our knowledge of the sector and discussions with the co-design workshops. We also illustrate in Figure 1 the relationship between the ToC and the five dimensions of effectiveness, which guide measurement [10].

[Figure 1: A theory of change for research-practice partnerships in adult social care]

Figure 1 is a preliminary ToC and a stepping-stone towards developing initial CMO hypotheses. The ToC does not illustrate well the trajectories to economic impact, hypotheses about how the context might trigger certain mechanisms and outcomes, nor our expectation that the journeys for each RPP will resemble a 'ripple effect', in that outcomes from earlier activities may form the context for later activities and outcomes [43]. Ultimately, systems diagrams with feedback loops to identify how inputs, activities and the outputs of those activities affect stocks of trust, and organisational capabilities to produce and use research, may be useful, as might participatory systems and ToC mapping approaches with the sites to identify

For peer review only - http://bmjopen.bmj.com/site/about/guidelines.xhtml

economic value and understand complex local systems [43,44]. The use of such approaches, however, will depend on willingness and progress made by the sites.

Stage 2: Conducting the evaluation through testing and refining theory

We will use a mixed-methods design for each case study [45], including semi-structured interviews, observation of RPP events and meetings, an online survey, activity diary and review of local data and documents. The different methods allow for evaluation of a broader range of outcomes, unintended consequences, and provide greater confidence in the measurement of key constructs and the evidencing of claims about the effectiveness of the new CCP partnerships. This approach is common among empirical studies of research use to make sure it is not over-estimated [46], and has been widely employed in RPP studies [7,10,47]. Data will be collected from August 2022 to October 2024 in multiple rounds and will guided by ongoing refinement of the ToC/programme theory.

To ensure we gather a range of perspectives on the development of the CCP partnerships, we will seek insights from members of RPPs (i.e. researchers, residents and family members, care home staff and other professionals who participate in the partnership) and wider stakeholders. These are people not directly involved in partnership work but who have a key stake in its success or influence its progress. They could include the university leadership, local authority staff (commissioners, social workers), owners or directors of care home groups, local trade associations, CQC inspectors, etc.

Survey

A web-based survey, designed using Qualtrics, will be sent to all RPP members at each partnership, on a roughly six-monthly basis, starting at baseline and around three to four further times over the project (see Supplementary file 1). As partnerships are small (we expect around ten people), the survey will take an enumeration sample of partnership members. The research team will send two reminders over a 6-week period to maximise response rates.

The questionnaire builds on a validated tool to evaluate the progress of US RPPs in the education context against the five outcome dimensions [48], but includes adaptations and changes to fit this context and address the research questions. The questionnaire captures trust and perceptions of relationships; whether the partnership has routines for doing and using research (capturing organisational capabilities); participation in partnership activities (capturing boundary working); relevance of research to practice; whether the partnership is achieving its goals; the impact of the partnership on the care home practitioners and their practice, and the wider sector; evidence of investment in the partnership and its members to enable everyone to participate fully.

To capture contextual factors of influence, we also included a question on individual skills and knowledge to participate in the partnership; a set of questions of particular relevance to practitioners on attitudes to research [49], and four-item personal research skills and knowledge sub-scale from the R&D culture index [50]; a set of

For peer review only - http://bmjopen.bmj.com/site/about/guidelines.xhtml

questions of particular relevance to researchers on personal co-production skills and knowledge [51]; a set of questions on their employer's (not the RPP's) research culture [49] and culture with respect to co-production; and a set of questions on identification with and commitment to the RPP that draw on Mael and Ashforth's [52] six-item scale of organisational identification and four items from Meyer and Allen's [53] affective commitment scale, dropping an item that could not be translated to this context and another that is not considered part of affective commitment [54]. In the final survey wave, for those questions that ask respondents to judge their skill or experience level, we will consider using retrospective pre-tests that allow respondents to rate themselves retrospectively from the beginning of the partnership compared with at the time of the final survey. This has been found to remove response shift bias and provide a more valid result than traditional pre- and post-test ratings when respondents are providing self-evaluations of their knowledge [55].

A survey of stakeholders might be warranted to capture what they view as the significance and value of partnership work. As its value depends on progress of the RPPs, it is not currently planned.

Interviews with CCP partnership members, wider stakeholders and CCP team members

For each partnership, we will hold one-hour long semi-structured interviews with CCP partnership members and stakeholders at the start of the partnership process and at three points thereafter. The number of interviews will be determined on a case-by-case basis, based on involvement in activities of the partnership, influence over the operation of the partnership and their ability to inform the research, but for CCP partnership members, it is likely to be around seven to eight interviews at each wave and for stakeholders around three. Initially RPP members and stakeholders will be chosen in consultation with the main site contact, but in order to minimise selection bias and the marginalization of people, a "snowballing" identification practice will be implemented.

Inevitably, as the partnerships develop the focus of the interviews will shift from capturing the setting up of the partnerships, to doing research as an RPP, and then to using research for organisational learning, wider knowledge exchange and impact beyond the partnership. Topic guides will be informed by the ToC and hypotheses about CMO combinations. Prompts and probes will ensure we explore power dynamics, trust, and the wider organisational and system context characterised by competing interests and values in shaping the trajectory of these partnerships.

We will also conduct one-hour semi-structured interviews with members of the CCP team who are leading the co-design, implementation and user and stakeholder involvement activities shortly after the co-design process has ended, and about three further times over the course of the implementation phase in broad alignment with the timing of support activities. The aim of these interviews is to capture the CCP team members' experience of delivering support activities to the partnerships and their views on how the partnerships are responding to the support, developing and using the support to shape their partnerships.

For peer review only - http://bmjopen.bmj.com/site/about/guidelines.xhtml

All interviews will be audio-recorded and transcribed. (See supplementary file 2 for interview proformas.)

Observation

We will conduct observation of partnership events for each case study to understand how the work of RPPs is being carried out in practice. The observations will focus on interactions, such as how the RPP members and attendees at events work together, make decisions and put their ideas and strategies into practice, and will be informed by an observational framework. Field notes will be written up for each event observed.

Activity diaries

To capture the time RPP members spend on different partnership activities, they will complete an activity diary. To facilitate entry in real-time, we propose that they use an existing time tracking app (Harvest, http://www.getharvest.com). Data will be visualised on an ongoing basis using the app and downloaded on a monthly basis.

We have included a development and testing phase to explore the best way of reporting activities and the feasibility of using the app at each site. Given the different types of activities that partnerships might use to enact RPP principles, we will use this phase to build up a categorisation for data entry. It will also help us understand the best way to integrate the collection of these data into RPP members' routines to ensure high quality data [56]. We will hold a workshop with each CCP partnership to develop solutions to these and other issues that members may have, including access to smartphones.

We will develop tailored guidance for the CCP partnerships and training that can be rolled out if new members join the partnership during the evaluation.

Routine and project-related data and local documents

Routine data, data related to research projects and documents produced by, for or about the partnership will be collected from each partnership on a regular basis. The aim of collecting this information is to provide insight into the plans for and activities of the partnership, research it is producing and using, and the relationship between the partnership and its parent and other organisations.

At this stage it is difficult to say what the information might look like, as it will be highly dependent on the plans and research agenda for each site. Based on learning from the interviews and observations, we will develop a template of the types of information we will request from the CCP partnerships on a quarterly basis. Examples might include, meeting minutes, data analysis notes, tools developed, grant proposals. This will ensure a degree of consistency in what we request from sites. The template will be reviewed and revised as the partnerships develop.

For peer review only

Reflecting on evaluation practice

The aim of the evaluation is not to provide a definitive judgement about the effectiveness of each RPP. We recognise that RPPs are on a journey and judgements about their value would be time-bound and unstable [57]. Instead, we aim to learn more about what can be achieved through the RPP approach as it is introduced in a new context and how the principles can be successfully enacted.

Although we are not providing a definitive judgement about the effectiveness of each new RPP, we will need to reflect critically on our practices and be sensitive to the ways in which they and the evidence we produce might influence the ways in which the CCP partnerships develop [58]. In this vein, we do not take a formative approach to the evaluation. This is in part to ensure a degree of independence, but more importantly, it ensures that our activity does not prevent the new partnerships from developing their own capacity to monitor and evaluate their work, since this might in the long-run undermine the sustainability of the partnership. We do recognise, however, that our evaluative judgements will be of value to the sites and have planned several feedback sessions. This will need to be situated within processes for learning and action, and will be delivered with the CCP implementation, and sustainability and spread teams. These workshops will enable sites to learn from the evaluation and use the information to improve how their partnerships are working.

Patient and public involvement

The CCP project includes a public member as part of the Management Team, who has the role of Involvement Lead. The involvement lead contributed to the development of the proposal, methods, and will advise the team throughout the various phases of the project.

The study has a lived experience reference group, comprising people with lived experience of receiving and giving care. Their main role is to provide support to the sites to involve the public and people with lived experience of giving and receiving care in their work. They also support the main CCP team, and have provided feedback on the data collection tools to ensure they are accessible. They will continue to advise the project team on the accessibility of data collection tools and outputs related to the project. We thank the advisors for their input to the evaluation.

ANALYSIS AND SYNTHESIS OF THE DATA

The aim of the analysis is to provide evidence about whether RPPs are a promising approach for driving improvements in practice in the care home context and to understand how, why and in what circumstances RPPs contribute to enhancing research and research use in local care homes and informing wider care home improvement efforts. Since this is a longitudinal evaluation, data will be gathered in waves and analysis will proceed iteratively, using evidence gathered from previous waves to inform subsequent data collection. Following each data collection wave

For peer review only - http://bmjopen.bmj.com/site/about/guidelines.xhtml

findings will be updated to generate a picture of how the CCP partnerships are developing over time, and the ToC/programme theory refined as we learn more about how and why the CCP partnerships are working and the kinds of impact they are having. At each wave the available data will be analysed in stages.

The first stage is to prepare descriptive profiles for each site. Each dataset will be analysed independently initially. We will use framework analysis [59], supported by Nvivo software to index the qualitative data (interviews, observation, document analysis) and identify evidence for outcomes, outputs, key constructs (e.g. boundary infrastructure), activities or strategies being enacted by the partnership. To inform decisions about whether or not data can be considered as evidence for or against outcomes, outputs and key constructs we will draw on theory and studies of research use [47,60]. Working within-case study sites, we will then compare across data types to triangulate evidence for each outcome, output and activity in a first stage of synthesis. This will enable us to develop outcome, output and activity profiles for each site, which will be used for the economic analysis.

Subsequently analysis will focus on the sub-questions, working first within case study sites then comparing across case study sites. The outcomes profile will enable us to assess sub-question 1a -- the extent to which outcomes have been achieved by each RPP. To address sub-question 1b and determine how significant a contribution the CCP partnership is making to the observed outcomes, we will use contribution analysis. We will follow the analytical steps outlined by Mayne and practical guidance [40–42] to use the evidence we gather to assemble and assess the contribution stories for how the partnerships have led to research being produced that is used to improve practice within the site and care improvements beyond the site. An important part of this analysis will be to understand the influence of the CCP co-design and implementation support teams. Comparing across case studies to identify whether patterns are consistent or are specific to particular CCP partnership will be important for ToC refinement.

We will complement our use of contribution analysis by drawing on realist methods to explore in more depth how, why and the circumstances in which the CCP partnerships contribute to each outcome (sub-question 1c). The focus will be on developing and refining links between CMOs, following guidance for realist evaluation [37], as well as exploring narrower aspects of causality within the broader ToC [61]. As the analysis progresses, we will explore how later CMOs relate to and might depend on earlier CMOs [62]. We will also investigate whether these patterns occur regardless of context, or are specific to particular CCP partnerships by comparing across sites. This analysis will provide insight, for example, into whether certain strategies are more suited to particular contexts.

Finally, we will explore whether the way in which the CCP partnerships are operating is consistent with the RPP approach (sub-question d). Additional coding schemes will be developed to capture who is involved in the activities, their context and purpose, the way in which they are being enacted (e.g. power differentials are present and not addressed), their consequences, and the contextual factors influencing the initiation and progress of the activities/strategies. As coding proceeds, the team will write memos to capture thinking around whether activities/strategies can be considered as faithful to the RPP approach, the

For peer review only - http://bmjopen.bmj.com/site/about/guidelines.xhtml

applicability of the RPP approach to the social care context and what these new partnerships can tell us about whether the core principles underpinning RPPs need to be adapted.

ECONOMIC EVALUATION

The economic evaluation will focus on exploring some of the more tangible economic consequences and utilise knowledge on indicators to model economic consequences for different types of outcomes. The analytical objectives for this stream are to establish the costs and economic consequences of RPPs, which combined will be used to derive an understanding of economic value of the RPP approach. The economic evaluation aligns with the theory-based evaluation and will draw on the data collection and analysis, using in particular the activity, output and outcomes profiles.

Full cost-effectiveness analysis would not be appropriate. Instead, we will use a 'narrative' economic analysis to examine both the costs of delivering the RPPs and some of the potential economic consequences. This method, widely used in the social care context draws on simulation modelling and cost-consequence analysis techniques [63–65]. It provides information on the estimated costs of an initiative and the estimated cost of alternatives enabling the decision maker to determine whether a course of action is worth investing in given the particular context in which they operate.

There are two parts to the analysis: part one, assesses the costs of delivering RPPs, and part two, models the economic consequences of RPPs. The two parts are subsequently synthesised to assess the value for money for each of the RPPs. We will take a health and social care and broader societal value perspective taken in the economic analysis. The latter will consider improvements in (health- or social care-related) quality of life, productivity and unpaid care.

Assessing costs

To cost the RRPs, we will use both bottom-up and top-down approaches [66]. Unit costs will be attached to each activity in the activity profile, using local sources where possible or – where this is not possible – adapted from national sources to reflect local salaries, overheads and capital costs. Budget information will be used where it is not possible to obtain bottom-up data on activities and for other resource use. Descriptive costs profiles will be developed for each site.

Modelling economic consequences

Potential economic consequences will be established through the ToC development and refinement process. As a first step, this will therefore include the further development of the outputs and outcomes profiles, to derive economic indicators,

For peer review only - http://bmjopen.bmj.com/site/about/guidelines.xhtml

and expected trajectories to potential economic impacts. From this, economic vignettes will be drawn for each site.

In a next step, monetary values will be assigned to outputs and outcomes identified in the vignettes as being linked to economic impacts. For some of the economic impacts it will be possible to attach monetary values either directly, or based on data from published sources (through modelling). An example of a consequence with direct monetary value is the income gained from a joint grant activity. An example of a consequence that would require further modelling to assign a monetary value is the implementation of an evidence-based intervention as part of service and quality improvements known to be cost-effective (such as the implementation of cognitive stimulation-therapy for people with dementia [67]). Modelling will use (where available) local data or information from the sites, and published data.

Since some of the economic gains will be realised during the research period whilst others will take place in the future, the analysis will have different time horizons (e.g. short-, medium-, long-term) reflecting differences in the certainty of (potential) economic gains. For example, it may be the case that a research project completed during the study period with known economic consequences for the care homes, but in another site a research project may only just have started or may still be at the planning stage, but nevertheless with expected but uncertain future economic consequences.

Cross-site comparison and synthesising costs and economic consequences

As economic consequences are likely to differ across sites, we need a way of structuring and categorising them to facilitate a narrative comparison between RPPs. Our starting point it to use the ‘Payback Framework’, which has been developed for examining the impact of health research [68]. It offers a multi-dimensional categorisation of benefits ranging from more traditional academic benefits of knowledge production to wider benefits to society, but it may need some adaptation to this context.

ETHICS AND DISSEMINATION

The study has undergone ethics review by the HRA Research Ethics Committee (REC) and has been reviewed in accordance with the London School of Economics (LSE) Research Ethics Policy and Procedure. The study does not pose major ethical issues or chance of harm for participants. Key issues relate to the observational component of the research and working with care home providers and ensuring steps to maintain confidentiality.

For peer review only - http://bmjopen.bmj.com/site/about/guidelines.xhtml

Processes for consent

Although the partnerships are obliged to participate in the evaluation, participation of individuals is voluntary. We will obtain written informed consent from all partnership members and stakeholders for all research activities that they will participate in. Consent will be obtained at the start of the research and again at the start of each research activity, with participants able to withdraw at any time.

Consent for observations of events and other activities related to the partnerships that involve people who are not closely connected to the partnership (and therefore have not previously given consent to be involved in research activities) will be achieved through negotiated and privileged access to the field and implied consent. An information sheet will be sent to participants in advance with the papers for events and a script prepared for the event Chair to introduce the researchers. Participants will have an opportunity to raise concerns at the start of any event and refuse permission for observation. Discussion of the CCP evaluation aims and objectives with the partnerships at the outset of their work and through personal conversations with the local evaluators will contribute to raising awareness and enabling implied consent.

Data Management and anonymisation

Data will be stored and managed in accordance with university and national rules and regulations as described in the project data management plan. Steps will be taken to minimise any risk of breaching confidentiality of research or personal data. Any personal information that could identify participants (such as name or job title) will be removed or changed before results are made public. All data collected from the activity diary and the survey will be reported at an appropriate level of aggregation so individuals cannot be identified.

Dissemination

Outputs will include interim case study reports and a comparative report for each analysis phase. The economic analysis will be conducted towards the end of the evaluation timeframe so will be included in the final report. An economic framework will be produced that can be used by those who want to replicate the analyses of economic value of RPPs.

The final analysis and synthesis will be published as a final report and articles covering the different aspects of the evaluation will be submitted to international journals. The final report will feed into three workshops to be held at the end of the project. These workshops will focus on the sustainability and spread of the RPP approach beyond the CCP sites to the rest of the UK, and sharing leaning from the study with interest groups, thought leaders, and senior policymakers in social care from across the UK.

For peer review only - http://bmjopen.bmj.com/site/about/guidelines.xhtml

AUTHORS' CONTRIBUTION

All authors contributed to conceptualisation of the study, with leadership from ABoaz for the CCP project, JM for the evaluation and ABauer for the economic evaluation. JM wrote the first draft with inputs from HK and ABauer. MK and ABoaz critically reviewed the manuscript and provided comments to improve it. All authors have read and approved the final manuscript.

COMPETING INTERESTS STATEMENT

The authors have no competing interests to declare.

FUNDING STATEMENT

This work was supported by the National Institute for Health and Care Research (NIHR) under the Health and Social Care Delivery research programme, grant number NIHR131335. The views expressed are those of the authors and not necessarily those of the NIHR or the Department of Health and Social Care.

ACKNOWLEDGEMENTS

We would like to acknowledge and thank our Creating Care Partnerships project colleagues (Ursula Ankeny, Bev Fitzsimons, Joe Langley, Becki Meakin, Stuart Muirhead, Becca Partridge, Naomi Raszyk, Lisa Smith, Melanie Weatherley and Claire Williams), participants in the co-design workshops and the project public advisors for their contribution to the development of the theory of change and data collection materials.

For peer review only

For peer review only - http://bmjopen.bmj.com/site/about/guidelines.xhtml

REFERENCES

1 Knapp M, Manthorpe J, Mehta A, *et al.* Developing the evidence base for adult social care practice: The NIHR School for Social Care Research. *J Care Serv Manag* 2010;**4**:167–79. doi:10.1179/175016810x12670238442101

2 Ghate D, Hood R. Using evidence in social care. In: Boaz A, Davies H, Fraser A, *et al.*, eds. *What works now? : evidence-informed policy and practice*. Bristol, UK: : Policy Press 2019. 89–109.

3 Metz A, Boaz A, Robert G. Co-creative approaches to knowledge production: What next for bridging the research to practice gap? *Evidence and Policy* 2019;**15**:331–7. doi:10.1332/174426419X15623193264226

4 Davies SL, Goodman C, Manthorpe J, *et al.* Enabling research in care homes: an evaluation of a national network of research ready care homes. *BMC Medical Research Methodology 2014 14:1* 2014;**14**:1–8. doi:10.1186/1471-2288-14-47

5 Walter I, Nutley S, Percy-Smith J, *et al.* Improving the use of research in social care practice. SCIE Knowledge Review 7. 2004.

6 Boaz A, Bauer A, Fitzsimons B, *et al.* Do Research Practice Partnerships offer a promising approach to knowledge production? . *Journal of Long-Term Care*

7 Penuel WR, Hill HC. Building a Knowledge Base on Research-Practice Partnerships: Introduction to the Special Topic Collection. *AERA Open* 2019;**5**:233285841989195. doi:10.1177/2332858419891950

8 Farrell C, Penual W, Coburn C, *et al.* Research-Practice Partnerships in Education: The State of the Field. 2021.

9 Arce-Trigatti P, Farrell C. Research–Practice Partnerships in Education Within The United States. *Education (Chula Vista)* Published Online First: 21 April 2021. doi:10.1093/OBO/9780199756810-0274

10 Henrick E, Cobb P, Penuel W, *et al.* Assessing Research-Practice Partnerships Five Dimensions of Effectiveness. New York, NY: 2017.

11 Booker L, Conaway C, Schwartz N. Five Ways RPPs Can Fail and How to Avoid Them: Applying Conceptual Frameworks to Improve RPPs. New York, NY: 2019.

12 Connolly F. Measuring the Value of a Research-Practice Partnership. *NNERP Extra* 2019;**1**:6–9.http://nnerppextra.rice.edu/measuring-the-value-of-an-rpp/ (accessed 24 Apr 2022).

13 Skivington K, Matthews L, Simpson SA, *et al.* A new framework for developing and evaluating complex interventions: update of Medical Research Council guidance. *BMJ* 2021;**374**. doi:10.1136/BMJ.N2061

14 Penuel W, Soland J, Farrell CC, *et al.* Assessing the outcomes of Research-Practice Partnerships: An Evaluation Argument. 2021.

For peer review only - http://bmjopen.bmj.com/site/about/guidelines.xhtml

15 Tseng V. The Next Big Leap for Research-Practice Partnerships: Building and Testing Theories to Improve Research Use. New York: 2017. http://wtgrantfoundation.org/library/uploads/2017/12/Next-Big-Leap_Vivian-Tseng_WTG_2017.pdf (accessed 24 Apr 2022).

16 Penuel W, Furtak EM, Farrell C. Research-practice partnerships in education. Advancing an evolutionary logic of systems improvement. *Die deutsche Schule* 2021;**113**:45–62.https://www.pedocs.de/volltexte/2021/22074/pdf/DDS_2021_1_Penuel_et_al_Research-Practice_Partnerships.pdf (accessed 24 Apr 2022).

17 Penuel W, Allen AR, Coburn C, *et al.* Conceptualizing Research–Practice Partnerships as Joint Work at Boundaries. *J Educ Stud Placed Risk* 2015;**20**:182–97. doi:10.1080/10824669.2014.988334

18 Farrell C, Penuel W, Allen A, *et al.* Learning at the Boundaries of Research and Practice: A Framework for Understanding Research–Practice Partnerships. *Educational Researcher* 2022;**51**:197–208. doi:10.3102/0013189X211069073

19 Farrell C, Coburn C. Absorptive capacity: A conceptual framework for understanding district central office learning. *Journal of Educational Change 2016 18:2* 2016;**18**:135–59. doi:10.1007/S10833-016-9291-7

20 Farrell CC, Harrison C, Coburn CE. "What the Hell Is This, and Who the Hell Are You?" Role and Identity Negotiation in Research-Practice Partnerships. *AERA Open* 2019;**5**:233285841984959. doi:10.1177/2332858419849595

21 Denner J, Bean S, Campe S, *et al.* Negotiating Trust, Power, and Culture in a Research–Practice Partnership: *https://doi.org/101177/2332858419858635* 2019;**5**:233285841985863. doi:10.1177/2332858419858635

22 Henrick E, McGee S, Penuel W. Attending to Issues of Equity in Evaluating RPP Outcomes. *NNERPP Extra* 2019;**1**:8–13.http://nnerppextra.rice.edu/attending-to-issues-of-equity-in-evaluating-rpps/ (accessed 4 Jun 2022).

23 Stanton MC, Ali SB, The SUSTAIN Center Team. A typology of power in implementation: Building on the exploration, preparation, implementation, sustainment (EPIS) framework to advance mental health and HIV health equity: *Implement Res Pract* 2022;**3**:263348952110642. doi:10.1177/26334895211064250

24 Kreindler S, Dowd D, Star N, *et al.* Silos and Social Identity: The Social Identity Approach as a Framework for Understanding and Overcoming Divisions in Health Care. *Milbank Q* 2012;**90**:347–74.

25 Hawe P, Shiell A, Riley T. Theorising interventions as events in systems. In: *American Journal of Community Psychology*. 2009. 267–76. doi:10.1007/s10464-009-9229-9

26 Farrell CC, Penuel WR, Allen A, *et al.* Learning at the Boundaries of Research and Practice: A Framework for Understanding Research–Practice Partnerships. *Educational Researcher* 2022;:0013189X211069073.

For peer review only - http://bmjopen.bmj.com/site/about/guidelines.xhtml

27 Blamey A, Mackenzie M. Theories of Change and Realistic Evaluation: Peas in a Pod or Apples and Oranges? *Evaluation* 2007;**13**:439–55. doi:10.1177/1356389007082129

28 Weiss C. Theory-based evaluation: past, present and future. *New Dir Eval* 1997;**76**:41–55. doi:10.1002/ev.1086

29 Mayne J. Contribution analysis: Coming of age?: *http://dx.doi.org/101177/1356389012451663* 2012;**18**:270–80. doi:10.1177/1356389012451663

30 Brousselle A, Buregeya JM. Theory-based evaluations: Framing the existence of a new theory in evaluation and the rise of the 5th generation: *https://doi.org/101177/1356389018765487* 2018;**24**:153–68. doi:10.1177/1356389018765487

31 Pawson R, Tilley N. *Realistic evaluation*. Sage Publications 1997.

32 Amon E, Hill S, Blake J, *et al.* Brokering Community-Based Research. *Mich J Community Serv Learn* 2020;**26**. doi:https://doi.org/10.3998/mjcsloa.3239521.0026.107

33 Mayne J. Theory of Change Analysis: Building Robust Theories of Change. *Canadian Journal of Program Evaluation* 2017;**32**:155–73. doi:10.3138/CJPE.31122

34 Morton S. Progressing research impact assessment: A 'contributions' approach. *Res Eval* 2015;**24**:405–19. doi:10.1093/RESEVAL/RVV016

35 Pawson R. Evidence-based policy: A realist perspective. In: *Making Realism Work: Realist Social Theory and Empirical Research*. 2004. doi:10.4324/9780203624289

36 Dalkin S, Lhussier M, Williams L, *et al.* Exploring the use of Soft Systems Methodology with realist approaches: A novel way to map programme complexity and develop and refine programme theory: *https://doi-org.gate3.library.lse.ac.uk/101177/1356389017749036* 2018;**24**:84–97. doi:10.1177/1356389017749036

37 Wong G, Westhorp G, Greenhalgh J, *et al.* Quality and reporting standards, resources, training materials and information for realist evaluation: the RAMESES II project. *Health Services and Delivery Research* 2017;**5**:1–108. doi:10.3310/HSDR05280

38 Jagosh J. Realist Synthesis for Public Health: Building an Ontologically Deep Understanding of How Programs Work, For Whom, and In Which Contexts. *Annu Rev Public Health* 2019;**40**:361–72. doi:10.1146/ANNUREV-PUBLHEALTH-031816-044451

39 Rycroft-Malone J, McCormack B, Hutchinson AM, *et al.* Realist synthesis: illustrating the method for implementation research. *Implement Sci* 2012;**7**. doi:10.1186/1748-5908-7-33

For peer review only - http://bmjopen.bmj.com/site/about/guidelines.xhtml

40 Delahais T, Toulemonde J. Applying contribution analysis: Lessons from five years of practice: *http://dx.doi.org/101177/1356389012450810* 2012;**18**:281–93. doi:10.1177/1356389012450810

41 Lemire ST, Nielsen SB, Dybdal L. Making contribution analysis work: A practical framework for handling influencing factors and alternative explanations. *Evaluation* 2012;**18**:294–309. doi:10.1177/1356389012450654

42 Mayne J. Contribution Analysis: Addressing Cause and Effect. In: Marra M, ed. *Evaluating the Complex*. New York: : Routledge 2011. 65–108. doi:10.4324/9781315147406-8/CONTRIBUTION-ANALYSIS-ADDRESSING-CAUSE-E

43 Barbrook-Johnson P, Penn A. Participatory systems mapping for complex energy policy evaluation: *https://doi.org/101177/1356389020976153* 2021;**27**:57–79. doi:10.1177/1356389020976153

44 Chazdon S, Emery M, Hansen D, *et al. A Field Guide to Ripple Effects Mapping*. University of Minnesota Libraries Publishing 2017. http://conservancy.umn.edu/handle/11299/190639 (accessed 17 Sep 2022).

45 Yin RK. *Case study research and applications —Design and methods*. 6th ed. Newbury Pk, CA: : Sage 2018.

46 Gitomer DH, Crouse K. Studying the Use of Research Evidence: A Review of Methods. 2019.

47 Farrell C, Davidson K, Repko-Erwin M, *et al.* A Descriptive Study of the IES Researcher-Practitioner Partnerships in Education Research Program: Final Report. Technical Report No. 3. National Center for Research in Policy and Practice. Web site: http://ncrpp.org/ 2018. www.ncrpp.org, (accessed 29 Sep 2021).

48 National Center for Research in Policy and Practice. Research-Practice Partnerships Outcomes Survey [field test version]. Boulder, CO: 2021.

49 Penuel W, Briggs D, Davidson K, *et al.* Survey of practitioner's use of research. 2016. https://osf.io/84hbu/ (accessed 1 Jun 2022).

50 Watson B, Clarke C, Swallow V, *et al.* Exploratory factor analysis of the research and development culture index among qualified nurses. *J Clin Nurs* 2005;**14**:1042–7. doi:10.1111/J.1365-2702.2005.01214.X

51 Francis-Smythe J, Haase S, Steele C, *et al.* Competencies and continuing professional development (CPD) for academics in knowledge exchange (KE) activity. 2006. https://eprints.worc.ac.uk/id/eprint/667 (accessed 17 Sep 2022).

52 Mael F, Ashforth BE. Alumni and their alma mater: A partial test of the reformulated model of organizational identification. *J Organ Behav* 1992;**13**:103–23. doi:10.1002/JOB.4030130202

53 Meyer JP, Allen NJ. A three-component conceptualization of organizational commitment. *Human Resource Management Review* 1991;**1**:61–89. doi:10.1016/1053-4822(91)90011-Z

For peer review only - http://bmjopen.bmj.com/site/about/guidelines.xhtml

54 Conway JM, Lance CE. What Reviewers Should Expect from Authors Regarding Common Method Bias in Organizational Research. *Journal of Business and Psychology 2010 25:3* 2010;**25**:325–34. doi:10.1007/S10869-010-9181-6

55 Howard GS, Dailey PR, Gulanick NA. The Feasibility of Informed Pretests in Attenuating Response-Shift Bias: *http://dx.doi.org/101177/014662167900300406* 2016;**3**:481–94. doi:10.1177/014662167900300406

56 Skivington K, Matthews L, Simpson SA, *et al.* A new framework for developing and evaluating complex interventions: Update of Medical Research Council guidance. *The BMJ* 2021;**374**. doi:10.1136/bmj.n2061

57 Schwandt TA, Gates EF. *Evaluating and valuing in social research*. New York: : Guilford Press 2021. https://www.guilford.com/books/Evaluating-and-Valuing-in-Social-Research/Schwandt-Gates/9781462547326 (accessed 4 Jun 2022).

58 Gates EF. Toward Valuing With Critical Systems Heuristics: *https://doi.org/101177/1098214017703703* 2017;**39**:201–20. doi:10.1177/1098214017703703

59 Ritchie J, Spencer L. Qualitative data analysis for applied policy research. In: Huberman AM, Miles MB, eds. *The qualitative researcher's companion*. Sage Publications 2002. 410.https://books.google.co.uk/books?hl=en&lr=&id=46jfwR6y5joC&oi=fnd&pg=PA305&dq=ritchie+spencer+framework+analysis&ots=soEWJQuuQR&sig=L5CSWX3hHT8XyZGhkbxdBF3JLgc#v=onepage&q=ritchie%20spencer%20framework%20analysis&f=false (accessed 6 Jun 2019).

60 Honig MI, Venkateswaran N, McNeil P. Research Use as Learning: The Case of Fundamental Change in School District Central Offices: *https://doi.org/103102/0002831217712466* 2017;**54**:938–71. doi:10.3102/0002831217712466

61 Rolfe S. Combining Theories of Change and Realist Evaluation in practice: Lessons from a research on evaluation study: *Evaluation* 2019;**25**:294–316. doi:10.1177/1356389019835229

62 Jagosh J, Bush PL, Salsberg J, *et al.* A realist evaluation of community-based participatory research: Partnership synergy, trust building and related ripple effects. *BMC Public Health* 2015;**15**:1–11. doi:10.1186/S12889-015-1949-1/TABLES/2

63 Knapp M, Bauer A, Perkins M, *et al.* Building community capital in social care: is there an economic case? *Community Dev J* 2013;**48**:313–31. doi:10.1093/CDJ/BSS021

64 Bauer A, Wistow G, Dixon J, *et al.* Investing in advocacy for parents with learning disabilities: what is the economic argument? *Br J Learn Disabil* 2015;**43**:66–74. doi:10.1111/BLD.12089

65 Bauer A, Taggart L, Rasmussen J, *et al.* Access to health care for older people with intellectual disability: A modelling study to explore the cost-effectiveness of

For peer review only - http://bmjopen.bmj.com/site/about/guidelines.xhtml

health checks. *BMC Public Health* 2019;**19**:1–16. doi:10.1186/S12889-019-6912-0/TABLES/7

66 Beecham J. Unit Costs-Not Exactly Child's Play. A guide to estimating unit costs for children's social care. Canterbury, Kent: 2000. https://www.pssru.ac.uk/pub/B062.pdf (accessed 4 Jun 2022).

67 D'Amico F, Rehill A, Knapp M, *et al.* Maintenance Cognitive Stimulation Therapy: An Economic Evaluation Within a Randomized Controlled Trial. *J Am Med Dir Assoc* 2015;**16**:63–70. doi:10.1016/J.JAMDA.2014.10.020

68 Donovan C, Hanney S. The 'Payback Framework' explained. *Res Eval* 2011;**20**:181–3. doi:10.3152/095820211X13118583635756

For peer review only

For peer review only - http://bmjopen.bmj.com/site/about/guidelines.xhtml

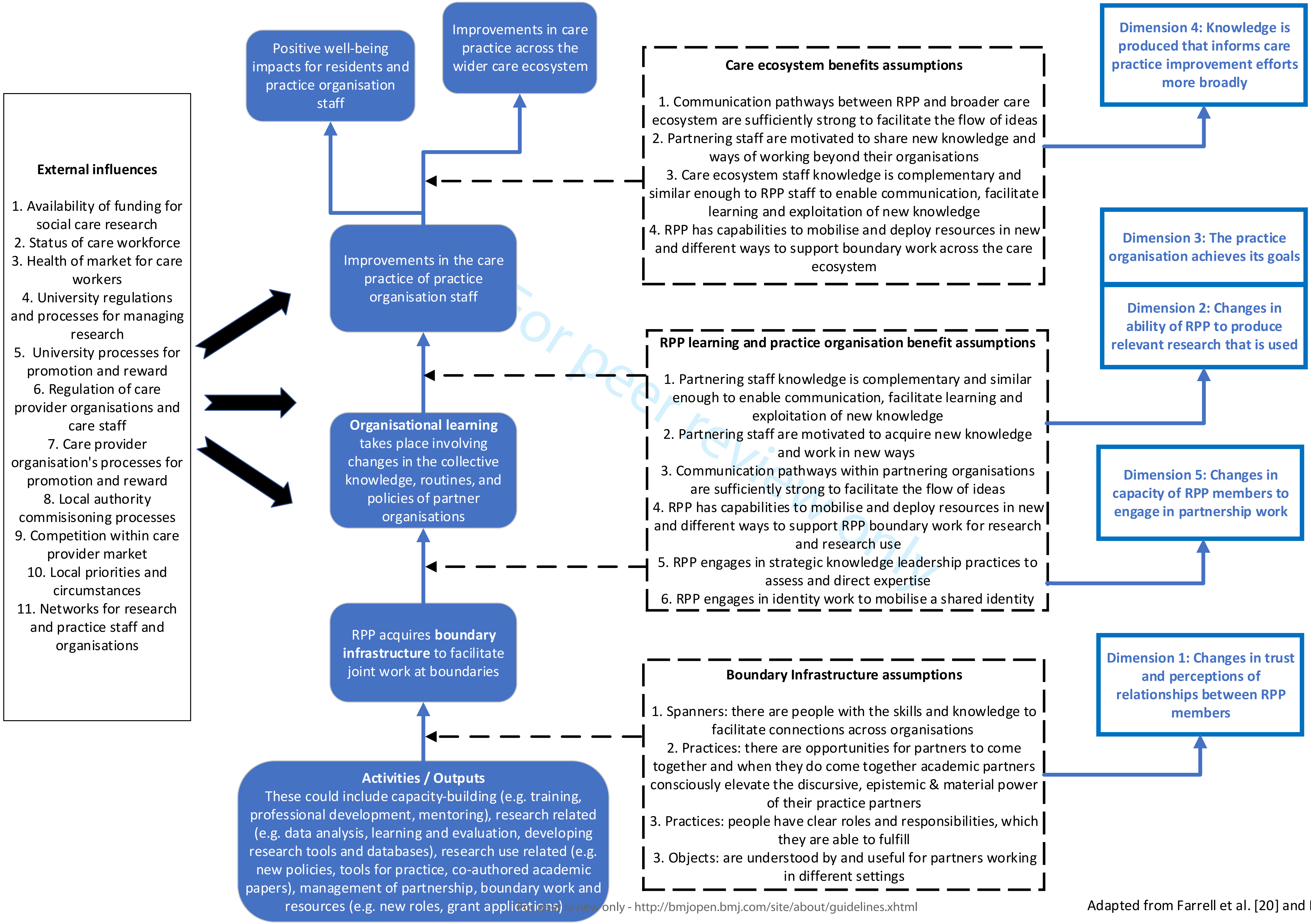

Adapted from Farrell et al. [20] and Henrick et al [10]

For peer review only - http://bmjopen.bmj.com/site/about/guidelines.xhtml

Supplementary file 1: Survey for CCP evaluation

This is a survey for CCP partnership members. It will be distributed by email and will be completed online, using Qualtrics software.

This questionnaire is based on a questionnaire that has been originally developed by the National Center for Research in Policy and Practice, Boulder, Colorado, USA. Full reference: National Center for Research in Policy and Practice. Research-Practice Partnerships Outcomes Survey [field test version]. Boulder, CO: 2021.

Due to the differences in context, some of the questions have been adapted to reflect the English social care/care home context. The questionnaire has been shortened to exclude some questions where data will be collected by other data collection methods. For example, we have excluded questions gathering factual information about partnership activities, as these will be collected via the activity diary. We also excluded questions asking about power dynamics between partners, as this will be captured through interviews and observations. Where the National Center for Research in Policy and Practice survey did not cover areas identified as important within our theory of change, we drew on other validated surveys to add questions. The scales used are cited in the main paper with full references, and are:

- *Questions on practitioners' attitudes to research from a survey by Penuel et al (2016)*
- *A four-item personal research skills and knowledge sub-scale from the R&D culture index by Watson et al (2005)*
- *Questions on the employer's (not the RPP's) research culture from a survey by Penuel et al (2016)*
- *Questions on identification with and commitment to the RPP that draw on Mael and Ashforth's (1992) six-item scale of organisational identification and four items from Meyer and Allen's (1991) affective commitment scale*

A question asking whether the respondent has completed the survey before has been added so that questions that would have the same response at each survey wave will be excluded following the first completion. We have also included q.2 to identify whether respondents identify as being from a research or a practice organisation, which will allow us to route respondents to relevant questions.

The introductory information for the survey has been removed.

For peer review only - http://bmjopen.bmj.com/site/about/guidelines.xhtml

Creating Care Partnerships (CCP) Evaluation Online Survey

Questionnaire

About the partnership and your role in the partnership

A note on how we're using the following terms in this survey:

Partnership: We use the term partnership to refer to the collaboration between research and practice that is part of the Creating Care Partnerships project. We are not referring to any other partnerships that your organisation may be involved in.

Partners: Individuals or groups who are actively involved in the work of the partnership. This might include care home managers, care home staff including nurses, care workers, activity providers, cooks, and other staff, care home residents, family members of care home residents, Local authority commissioners, other local leaders, and researchers.

Non-academic partners: Individuals or groups who are involved in the work of the partnership who are not employed within Higher Education Institutions or other research organisations. This might include care home managers, care home staff including nurses, care workers, activity providers, cooks, and other staff, care home residents, family members of care home residents, Local authority commissioners, other local leaders.

My organisation: This relates the organisation you are employed with.

Stakeholders: Individuals or groups who have a stake or interest in issues relevant to caring within a care home but who are not necessarily actively involved in the partnership.

Research: An activity in which people aim to answer questions by using evidence from observation or experience that has been specifically collected to answer the question. This is different from using data which is already collected for more general purposes by care homes, local authorities or nationally.

Practice: issues of importance to care homes or social care, care delivery, residents/families, and care home staff.

For peer review only

For peer review only - http://bmjopen.bmj.com/site/about/guidelines.xhtml

Q1. Please indicate below if this is your first time completing this survey.

1. Yes
2. No

If Q1=2 then do not ask questions 2-4

Q2. What type of organisation do you work for?

1. Care Home provider
2. Local authority
3. Other social care organisation
4. Higher Education Institution
5. Other research organisation
6. Other (please specify)

Q3. What is the name of the organization or institution that you work for?

[open response]

Q4. How would you identify your primary role in the context of this partnership?

1. Care assistant
2. Senior care assistant
3. Care home deputy / assistant manager
4. Care home manager
5. Care home group leader/manager (central office)
6. Registered nurse
7. Social worker
8. Researcher
9. Research nurse
10. Other, please specify

Q5. Overall, how satisfied are you in your job?

1. Very satisfied
2. Quite satisfied
3. Neither satisfied nor dissatisfied
4. Quite dissatisfied
5. Very dissatisfied

Q6. Do you have a formal leadership role within the partnership?

1. Yes
2. No
3. Not sure

For peer review only - http://bmjopen.bmj.com/site/about/guidelines.xhtml

If Q6 = 2-3 then ask

Q7. Can you describe this role?

Insert free text answer

Building trust and cultivating partnership relationships

If Q1=2 then do not ask

Q8a. Did you know any of the other partnership members before this partnership's work began?

1. Yes, I knew at least one other partnership member.
2. I didn't know any partnership members personally, but another person whom I trusted did.
3. No, I didn't know any partnership members at all.

Display This Question: If Did you know any of the other partnership members before this partnership's work began? = Yes, I knew at least one other partnership member.

If Q1=2 then do not ask

Q8b. In what way did you know other partnership member(s) before this partnership's work began?

Select all that apply.

1. I knew them personally, outside of professional work.
2. I worked with them previously when we were employed within the same organisation
3. I have collaborated with them previously, but not in a formal partnership
4. I worked with them in a formal partnership.

Q9. Indicate how much you agree or disagree with each statement.

	Strongly Disagree	Disagree	Neither agree nor disagree	Agree	Strongly Agree
Our partners follow through when they commit to something.					
Our partners finish tasks when they say they will.					
Our partners' work is reliably of high quality.					
We have supports in place to help people					

For peer review only - http://bmjopen.bmj.com/site/about/guidelines.xhtml

follow through with their commitments.					
I can count on my partners to help me outside of our formal commitments.					

Q10. Indicate how much you agree or disagree with each statement.

	Strongly Disagree	Disagree	Neither agree nor disagree	Agree	Strongly Agree
Partners listen to what each person has to say.					
Partners withhold information that is relevant to the partnership.					
Partners feel comfortable discussing an issue when a conflict arises.					
Even when we disagree, I feel that my opinions have been recognized.					
I don't feel comfortable raising concerns I have.					

Q11. Indicate how much you agree or disagree with each statement.

	Strongly Disagree	Disagree	Neither agree nor disagree	Agree	Strongly Agree
I trust my partners are honest with me					
Even in difficult situations, partners can depend on one another.					
I trust my partners will share important information with me					
Partners tend to do what is in their interest, regardless of whether it benefits the partnership.					

For peer review only - http://bmjopen.bmj.com/site/about/guidelines.xhtml

It is difficult to work in our partnership because of existing mistrust.					

Q12. Indicate how much you agree or disagree with each statement.

	Strongly Disagree	Disagree	Neither agree nor disagree	Agree	Strongly Agree
I feel good about working with other people in this partnership.					
All partners have something valuable to contribute.					
Partners have high and reasonable expectations for each other’s contributions.					
Partners sometimes have to guess about each other's needs.					
Partners treat each other with care.					
Partners are continually learning new things that are useful in their daily lives.					

Conducting relevant research to inform care home practice

Q13. To the best of your knowledge, how often has your partnership discussed the following in partnership meetings in the past year?

	At every meeting	At most Meetings	Occasionally	Rarely	Not at all
Ideas from research conducted in other contexts					
Data collection or analysis strategies					
Quantitative data representations (e.g., graphs)					

CCP partnership member survey, CCP Project, v3.0
10/06/2022

For peer review only

Qualitative data e.g., interview transcripts)					
Findings and implications for action					
Equalities, diversity, and inclusion considerations to the work					

Q14. In our partnership, I have participated in...

(Mark all that apply.)

1. Deciding what topics and issues to research
2. Developing a grant proposal
3. Developing a literature review
4. Creating a research design
5. Gathering new or existing data
6. Developing resources for storing and accessing data
7. Helping to analyse data
8. None of the above

Q15. In our partnership, I have participated in...

(Mark all that apply.)

1. Organising opportunities for partners to discuss research/inquiry findings
2. Sharing findings
3. Sharing my thoughts on research findings
4. Taking on a specific role to communicate findings across academic and non-academic partners
5. Developing new interventions, strategies, or tools based on our findings
6. None of the above

Q16. In our partnership, I have participated in...

(Mark all that apply.)

1. Co-authoring written outputs from the research
2. Co-designing products developed by the partnership, e.g. innovative practices, guidelines, materials etc
3. None of the above

Q17. Indicate how much you agree or disagree with each statement.

Our partnership's work...

For peer review only - http://bmjopen.bmj.com/site/about/guidelines.xhtml

	Strongly Disagree	Disagree	Neither agree nor disagree	Agree	Strongly Agree
Addresses a central concern for care home practice					
Addresses a pressing need of those who have a stake in or are affected by care homes.					
Addresses a gap in the academic literature					

Supporting the non-academic partner organisation in achieving its goals

Q18a. Which best describes the extent to which your partnership has made progress on its goals, aims or objectives?

1. We have not identified goals, **aims or objectives** and do not plan to.
2. We are in the process of identifying goals, **aims or objectives**.
3. We have specified goals, **aims or objectives** to address an issue that is important to non-academic partners
4. We have made progress in working toward our goals **aims or objectives**.
5. We have accomplished some of our goals.
6. We have accomplished and are extending our goals.

Display This Question:

If Which best describes the extent to which your partnership has made progress on its goals? = We have specified to goals to address an issue that is important to non-academic partners.

Or Which best describes the extent to which your partnership has made progress on its goals? = We have made progress in working toward our goals.

Or Which best describes the extent to which your partnership has made progress on its goals? = We have accomplished some of our goals.

Or Which best describes the extent to which your partnership has made progress on its goals? = We have accomplished and are extending our goals.

Q18b Indicate how much you agree or disagree with each statement.

	Strongly Disagree	Disagree	Neither agree nor disagree	Agree	Strongly Agree

For peer review only - http://bmjopen.bmj.com/site/about/guidelines.xhtml

We are working toward a common set of goals.					
We have some goals in common and some goals that differ.					
Partners hold substantially different goals.					
Our goals were set by a narrow group, and stakeholders did not have opportunities to contribute.					
In creating or revising our goals, there were multiple opportunities for stakeholders to be involved.					

Q19. Which is most true for your partnership's impact on the care home partner organisation's decisions?

Our partnership...

1. Is too new to have impacted decisions yet.
2. Aims to impact decisions, but we have run into challenges.
3. Is in the process of informing decisions, but no changes have been made yet.
4. Has impacted decisions in the care home partner organisation
5. Does not aim to impact the care home partner organisation's decisions.

Display This Question:

If Which is most true for your partnership's impact on the care home partner organisation's decisio... = Has impacted decisions in the practice/community organization.

Q20. Due to the partnership's influence, the care home partner organisation has...

(Mark all that apply.)

1. Adopted new policies, programs, or practices
2. Participated in designing new policies, programs, or practices
3. Improved existing policies, programs, and practices
4. Allocated more resources to particular policies, programs, or practices
5. Ended support for existing policies, programs, or practices
6. Designed new professional learning opportunities or training

For peer review only - http://bmjopen.bmj.com/site/about/guidelines.xhtml

For peer review only

7. Created a new framework or set of ideas to help think about how to address care home issues
8. Other (please specify)

Q21. Indicate how much you agree or disagree with each statement.

Because of this partnership, non-academic partners...

	Strongly Disagree	Disagree	Neither agree nor disagree	Agree	Strongly Agree
Are more informed by research in their thinking across a variety of decisions.					
More often turn to research to directly inform the decisions they make.					
More often point to research to persuade others.					
More often point to research to justify a decision that has already been made.					
More often use varied forms of research evidence in making decisions.					

Producing knowledge that can inform care home and social care improvement efforts more broadly

Q22. What is most true about your partnership's efforts to share knowledge created through the partnership's work with audiences outside of the partnership?

1. We actively share knowledge in a range of forms and venues.
2. We share knowledge in some ways but would like to do more.
3. We have plans to share knowledge but have not yet done so.
4. It is not a priority to share knowledge created by our partnership.

For peer review only - http://bmjopen.bmj.com/site/about/guidelines.xhtml

Display This Question:

If What is most true about your partnership's efforts to share knowledge created through the partner... = We actively share knowledge in a range of forms and venues.

Or What is most true about your partnership's efforts to share knowledge created through the partner... = We share knowledge in some ways but would like to do more.

Or What is most true about your partnership's efforts to share knowledge created through the partner... = We have plans to share knowledge but have not yet done so.

Q23. Has your partnership identified external audiences for your work?

1. We have identified multiple audiences, including research, care home/social care, and non-academic audiences for our work
2. We identified either research or care home/social care, non-academic audiences outside of our partnership for our work.
3. We have not begun identifying audiences outside of our partnership, but are interested in doing so.

Display This Question:

If What is most true about your partnership's efforts to share knowledge created through the partner... = We actively share knowledge in a range of forms and venues.

And What is most true about your partnership's efforts to share knowledge created through the partner... = We share knowledge in some ways but would like to do more.

And What is most true about your partnership's efforts to share knowledge created through the partner... = We have plans to share knowledge but have not yet done so.

Q24. In which ways does your partnership share knowledge?

	Already do	Plan to do	Have not done and do not plan to do
Online media (e.g., website, blog, webinars, podcasts, newsletters, social media, etc.)			
Research-focused products (e.g., journal articles)			
Practice, care home/social care or policy-focused products (e.g., magazine articles, briefs, reports)			
Presentations at meetings for research audiences			

For peer review only - http://bmjopen.bmj.com/site/about/guidelines.xhtml

Presentations at meetings for practice, care home/social care, or policy audiences			
Storytelling or theatrical representations			
Makes connections with other networks or expands work to other settings			
Acts as a resource for other partnerships			
Has staff with dedicated communications responsibilities			
Applies for further funding to upscale partnership research project			
Other (please specify)			

Q.25. Has your partnership engaged with any of the following groups or networks to share knowledge?

	Already do	Plan to do	Have not done and do not plan to do
Applied Research Collaborations (ARCs)			
NIHR Enabling Research in Care Homes (ENRICH)			
Local and regional provider associations			
Association of Directors of Adult Social Services (ADASS)			
Professional bodies (e.g. British Association of Social Workers, Royal College of Nursing)			
Research in Practice for Adults (Ripfa)			
Social Care Institute for Excellence (SCIE)			
Other research networks (please specify)			
Local and regional provider associations			
Association of Directors of Adult Social Services (ADASS)			

For peer review only - http://bmjopen.bmj.com/site/about/guidelines.xhtml

Building the capacity of participating researchers, practitioners, care homes, and research organisations to engage in partnership work

Q26. Our partnership has created opportunities for partners to learn more about the following aspects of the research process:

(Mark all that apply.)

1. Asking research/inquiry questions that matter
2. Collecting data using different methods
3. Analysing data and interpreting findings
4. Presenting findings for different audiences
5. Knowing how to link research results to key issues facing decision-makers
6. None of the above

Q27. Our partnership has created opportunities for partners to learn more about the following aspects of co-design between academic and non-academic partners:

(Mark all that apply.)

1. Designing, testing, and/or adapting delivery processes or materials
2. Designing, testing, and/or adapting professional development within care homes
3. None of the above

Q28. Our partnership has created opportunities for partners to learn more about the following aspects of the practice-based issue and local context:

(Mark all that apply.)

1. A broader system perspective (i.e., the range of individuals, organisations, and networks that influence the issue at hand)
2. How decision-making unfolds in care homes and/or local authorities
3. The content of the issue at hand
4. The historical, political, or other equity dimensions of the issue at hand
5. Resources in local communities or organizations
6. None of the above

Q29. As a result of working together, participating organisations have...

(Mark all that apply.)

1. Created new positions (e.g., partnership intermediary, broker, or project manager)
2. Revised job descriptions
3. Submitted grant proposals to support the work
4. Dedicated financial resources to support the partnership
5. Dedicated in-kind resources to support the partnership (e.g., staff time, office space)
6. Changed formal policies (e.g., guidelines for promotion)

For peer review only - http://bmjopen.bmj.com/site/about/guidelines.xhtml

7. Altered or developed new evidence based interventions
8. Adopted routines modelled in the partnership's work
9. Produced outputs that are of value the wider research and practice community
10. None of the above

Q30. Indicate how much you agree or disagree with each statement.

In terms of our research/inquiry activities, all partners...

	Strongly Disagree	Disagree	Neither agree nor disagree	Agree	Strongly Agree
Are committed to doing their part.					
Have the time and resources to do their part.					
Have the knowledge and skills needed to do their part.					
Think that the partnership is worthwhile					
All partners are equally involved					

Q31. Does your partnership have a sense of community among its members?

1. We have not yet considered how this partnership could build a sense of community among its members.
2. We see the benefit of building a sense of community among members but are unsure of how to proceed.
3. We are in the process of building a sense of community among members.
4. Our partnership activities have built a sense of community among members.

Q32. What best describes partners' attention to the overall progress of your partnership?

1. We don't pay attention to the progress made by the partnership.
2. We keep our partnership's progress in mind, but we rarely discuss it.
3. We occasionally discuss the overall progress of our partnership and make adjustments if needed.
4. We regularly discuss the progress of our partnership and make adjustments.

Q33. Indicate how much you agree or disagree with each statement.

For peer review only

For peer review only - http://bmjopen.bmj.com/site/about/guidelines.xhtml

	Strongly Disagree	Disagree	Neither agree nor disagree	Agree	Strongly Agree
When someone criticizes the partnership, it feels like a personal insult					
I am very interested in what others think about the partnership					
When I talk about this partnership, I usually say 'we' rather than 'they'					
This partnership's successes are my successes					
When someone praises this partnership, it feels like a personal compliment.					
If a story in the media criticized the partnership, I would feel embarrassed					

Q34. Indicate how much you agree or disagree with each statement.

	Strongly Disagree	Disagree	Neither agree nor disagree	Agree	Strongly Agree
I do not feel a strong sense of belonging to the partnership					
I do not feel emotionally attached to this partnership					
I do not feel like part of the family at my partnership					
This partnership has a great deal of personal meaning for me					
I would like this partnership to have more personal meaning for me.					

For peer review only - http://bmjopen.bmj.com/site/about/guidelines.xhtml

Q35. Indicate how much you agree or disagree with each statement.

In terms of our research/inquiry activities, I…

	Strongly Disagree	Disagree	Neither agree nor disagree	Agree	Strongly Agree
I am committed to doing my part.					
I have the time and resources to do my part.					
I have the knowledge and skills needed to do my part.					
I feel supported by my organisation to do my part					

If Q2 = 1-3, 6 then ask.

Q36. Indicate how much you agree or disagree with each statement

	Strongly Disagree	Disagree	Neither agree nor disagree	Agree	Strongly Agree
I understand research terminology					
I feel confident about using research in my practice					
I know how practice is influenced by research					
I have the skills to use the library and learning facilities					

If Q2 = 1-3, 6 then ask.

Q37. I feel confident that I have the knowledge and skills to:

1. Find research to inform policy, care delivery change, practices
2. Evaluate the quality of research
3. Interpret the results of research
4. Apply research to policies, programmes, or practice development
5. Design evaluations of policies, programmes, or practices
6. Commission research to support policies, programmes, or practice development

If Q2 = 4-5 then ask

Q38. Indicate how good you are at the following:

For peer review only - http://bmjopen.bmj.com/site/about/guidelines.xhtml

	Excellent	Good	Average	Poor	Very Poor
Acknowledging expertise of non-academic partners					
Being able to work with individuals in non-academic partner organisations					
Building relationships with non-academic partners					
Building rapport through regular contact with non-academic partners					
Being collaborative, not being telling nor controlling					
Engaging with non-academic partners without superior attitude					
Communicating research in plain language					
Explaining complex ideas in a way that non-academic partners can understand					
Understanding the perspectives of non-academic partner's perspectives and showing empathy					

If Q2 = 4-5 then ask

Q39. Indicate how often the following happen:

	Never	Sometimes	Frequently,	All of the time
In my organisation, practitioner's views are seen as a useful source of information for making decisions about what research should be done				

CCP partnership member survey, CCP Project, v3.0
10/06/2022

For peer review only

In my organisation, there is encouragement to work closely with social care practitioners throughout the research process				
In my organisation, it is expected that you will work with practitioners to conduct research that will improve care homes				

If Q2 = 1-3, 6 then ask

Q40. Indicate how often the following happen:

	Never	Sometimes	Frequently,	All of the time
In my organisation, research is seen as a useful source of information				
In my organisation, there is encouragement to use research as part of our ongoing work				
In my organisation, studies are conducted on changes to care delivery/new processes/policies that are implemented to see how they work				
In my organisation, it is expected that if you make a claim in a meeting, you will be able to cite research evidence to back it up				
In my organisation, interaction or collaboration with researchers or research organisations is encouraged				

If Q2 = 1-3, 6 then ask

CCP partnership member survey, CCP Project, v3.0
10/06/2022

For peer review only

Q41. Indicate how much you agree with the following statements:

	Strongly Disagree	Disagree	Neither agree nor disagree	Agree	Strongly Agree
Research helps identify problems facing care homes					
There is a disconnect between the research world and the care home world					
Research addresses questions that help us make better decisions about social care					
When confronted with a new problem or decision, it is valuable to speak with social care researchers					
Social care research is too narrow to be useful to social care leaders or managers					
Social care researchers work in an ivory tower and are isolated from practice					
By the time research findings are published they are no longer useful to me					
Research can address practical problems facing care homes					
Researchers provide a valuable service to social care leaders, managers and workers					
Social care researchers are unbiased					

About You

The remaining questions will allow us to better understand your background. You do not have to answer them if you do not want to.

If Q1=2 then do not ask

Q42. I am:

1. Male
2. Female
3. Non-binary
4. Prefer not to say

CCP partnership member survey, CCP Project, v3.0
10/06/2022

For peer review only - http://bmjopen.bmj.com/site/about/guidelines.xhtml

Q43. Which of the following best describes your ethnic group?

1. White (e.g. English, Irish, Scottish)
2. Mixed/multiple ethic group (White and Black Caribbean, White and Black African, White and Asian)
3. Asian/Asian British (e.g. Indian, Pakistani, Bangladeshi, Chinese)
4. Black/African/Caribbean
5. Other (please specify)

Q44. How old are you?

1. 18 years old or younger
2. 19-25 years old
3. 26-30 years old
4. 31-35 years old
5. 36-40 years old
6. 41-45 years old
7. 46-50 years old
8. 51-55 years old
9. 56-60 years old
10. 61 years old or older

Q.45. What is your highest level qualification?

1. None
2. NVQ Level 1
3. NVQ Level 2
4. NVQ Level 3
5. NVQ Level 4
6. NVQ Level 5
7. GCSE (s) (or equivalent)
8. AS level(s) (or equivalent)
9. Undergraduate degree
10. Masters degree
11. Doctorate

For peer review only

For peer review only - http://bmjopen.bmj.com/site/about/guidelines.xhtml

Supplementary file 2: Interview proformas for CCP evaluation

RPP member interview proforma

This proforma will be used for RPP member interviewees, which includes those involved in the design and implementation of one of the three RPP case study sites. These could include care home managers, university researchers, care staff, nurses, members of staff within the local authority or charitable organisations involved with the RPP.

The questions are largely framed as they will be used for the initial interviews, but these will change for later interviews in the way described in the text boxes at the start of each section. We refer to this interview schedule as a proforma to reflect the fact that the content covers the breadth of issues we will want to investigate but questions will not be relevant for everyone at each time point.

At present none of the partnerships have representatives who are care home residents or family members. If membership changes the proforma will be adapted for interviewing these members.

A. Introductory information

My name is XXXXXX. Thank you for agreeing to take part in the Creating Care Partnerships (CCP) study.

[name of partnership] has been selected as one of the case studies for the CCP study. We are interviewing members of [name of partnership] to get a better understanding of your experiences of implementing the RPP approach in care homes in England; how, why and in what circumstances the RPP approach contributes to enhancing the quality of research and research use in care homes in England; and the costs and benefits of delivering RPPs.

The interview should last about an hour. During the interview I will ask you about how your partnership is developing its capacity for doing and using research to benefit practice in care homes, the types of activities you do as a partnership and the consequences of those activities. I will also ask you about importance of external events and local conditions in influencing how your partnership works and its ability to achieve its goals.

I am sure there will be some areas you have more knowledge about. Where there are gaps in your knowledge it would be helpful if you could identify people or key documents that may help us to better understand that part of partnership working.

For peer review only - http://bmjopen.bmj.com/site/about/guidelines.xhtml

Informed Consent

1. Can I just check that you received the study information sheet and that you have had a chance to read it?

   No - [Review the study information sheet in detail]
   Yes - *Good* [Review the study information sheet briefly]

2. Do you have any questions about the wider study or about the interview we will be conducting today?

3. If you are happy to please can you sign the consent form that I sent you and email it back to me for our records. The consent form is a standard form used in social research and is used to ensure that:

- you understand the aims of the study
- you understand what your participation in the study will involve
- you are happy for the discussion to be audio recorded
- you know that you can change your mind about taking part in the study at any time – you can request for the discussion to stop at any time and if at any point you want to withdraw from the study you can request this by using the contact information provided to you today
- you know that your views will be kept confidential and your name will never be used in anything that is written about the study
- you consent to take part.

[Note: if the participant is not able to sign and return the consent form then consent can be recorded orally.]

[Start recording] Software will request participant to give permission to start recording. If face-to-face then request permission to start recording

B. Introductory questions

1. Can you tell me about your current professional role at [name of organisation] and how long you have been in post?

2. What's a typical day for you?

C. Contextual information about the partnering organisations

The aim of this section is to gain some contextual information about [name of partnership] members and the organisation they are employed by. We also want to

For peer review only - http://bmjopen.bmj.com/site/about/guidelines.xhtml

explore the personal and organisational motivations for participating in [name of partnership], and understand the background to the relationships in the partnership.

i) Information about the organisation

HEI interviewees

1. Can you me a bit about your department's research interests and ongoing projects? What other organisations does it normally collaborate with?

2. How does your university relate to other organisations/universities in the region? Does it have a strategy with respect to how it works with organisations in its region? Is the university involved in any major regional cross-sector partnerships, e.g. ARCs?

Care Home/local authority/third sector interviewees

1. How would you describe your organisation? What organisations does it normally collaborate with?
   Probe around:
- Organisational type
- The area the organisation serves
- Population it serves/demographic

ii) Reasons for participation in the partnership

1. Can you tell me why your organisation wanted to participate in the [name of partnership]?

2. Can you describe how the partners came together to start the [name of partnership] and respond to the EOI?
   Probe around understanding the initial power dynamics:
- Whether one person or partner took the lead and brought all the partners together or whether partners / people were suggested by different members
- Whether they came together at all, had meetings over the phone, who was involved in the decisions and writing the EOI.
- Whether they discussed resources and how those would be held and parcelled out.
- Whether they discussed a structure for the management and organisation of the partnership, what roles people would have
- Whether they discussed what they wanted to achieve and how they would achieve it
- Whether they discussed ways of working

For peer review only - http://bmjopen.bmj.com/site/about/guidelines.xhtml

D. Perspectives of the partnership member on their role and the partnership

The aim of this section is to explore the personal motivations for participating in [name of partnership], understand the relationships in the partnership and the member's role and capacity to fulfil that role in the partnership.

i) Reasons for participation in the partnership

1. Can you tell me about why you personally wanted to participate in the [name of partnership]?

ii) Experience of research-practice partnership working and relationships between members (previous and existing)

The aim of this section is to collect data on the their views about the potential for research to inform and improve practice, the relationships between partnership members and how these evolve over the course of the partnership. In early interviews we will ask about the formation of relationships prior to the official start of the partnership, but in later interviews we will look at how perceptions of partners are changing.

The issues to dig into are the shifting power dynamics as the partnership progresses, in what respects different partners hold power, and how has it been relinquished, contested, and held onto. We also want to explore the cycles of relationship building, maintenance, breakdown and repair as the partnership progresses and how these feed into future partnership work.

3. Before you started/joined this partnership how familiar were you were working in partnerships with other organisations to produce research for practice improvement? What view did you have about the potential for research to inform improvements in practice? Do you think your previous experience has had an influence on how you have approached this partnership?
   Note: for academic partners we are interested in understanding about their experience of previously working with partners from practice around research
   Probe around:

- What your previous experiences were like and what you learnt from those previous experiences, focus on experiences or research / experience of working with practitioners to co-produce research

For peer review only - http://bmjopen.bmj.com/site/about/guidelines.xhtml

- If no direct experiences, then explore whether they learnt from the experience of others and who they connected with / what they may have read
- How the previous experience/experiences of others influences how you approached this partnership
- Whether working in the partnership has changed their mindset, whether they see a greater / different potential for using research to inform practice

4. Before you started/joined this partnership how familiar were you with your partner organisations in [name of partnership] or members of those organisations? (academic researchers/care homes/social care) Do you think this has had an influence on how your partnership is developing?
   Probe around:
- Your initial perceptions of your partners? What influenced these perceptions?
- Whether the previous relationships were personal or in the context of work, and what the previous work was
- Why they decided to work with the organisation

5. Have relationships been something you have discussed as a partnership, and if so why? Have you felt that it would be helpful to get to know members of your partnership better? Why did you think this would be helpful?

6. Have you decided to do anything as a partnership to get to know each other better or change the way you relate to each other? What have you done and how has this affected the partnership?

Probe around:

- Ways to involve people in the work
- Ways to ensure people have more power and voice in processes and decision-making forums
- Communication pathways to facilitate involvement

7. Have there been any difficult moments within the partnership, where people have disagreed over the direction of the partnership? How have these moments of conflict been managed and resolved?
   Probe around:
   - How did you felt during these times
   - Strategies developed to deal with conflict
   - Whether the types of conflicts and methods of resolution change over time
   - Role of power in this process (dominance, resistance, power plays)

iii) <u>What the member brings to the partnership and their role in it</u>

For peer review only - http://bmjopen.bmj.com/site/about/guidelines.xhtml

The aim of this section is to explore the way in which individual's capacity to conduct partnership work has built up over time.

In the early stages we will concentrate more on the pre-existing skills, capacities and knowledge of partners before moving on to look at the types of individual dispositions, skills and capacities that were needed to engage in partnership work and whether their previous experiences enabled or inhibited this. In the later stages we will concentrate on how skills and capabilities for conducting research and joint working developed over time and the influence of the partnership members and infrastructure, wider networks and resources.

1. What skills and knowledge do you think you bring to the [name of partnership] and why do you think they will be/are helpful?
   Probe around:
- Research skills/experience
- Knowledge of care homes, service delivery, patient/resident population, etc.
- Partnership working experience

2. Can you describe the role you occupy in the [name of partnership]? What role do you expect to occupy?
   Probe around:
- Formal roles and informal roles – what responsibilities they have or have taken on
- Elements of your usual professional role that are most useful to this role
- New aspects/perspectives you needed to take on to engage in your partnership role
- Attitudes/dispositions/values/skills you think make a good partnership worker
- Experience of conflict or difficulties when taking on this new role and how you managed those

3. Can you describe how your role in the partnership has developed over time? Why did/didn't it develop? Would you like your role to develop further?
   Probe around:
- How skills and knowledge have developed over time in relation to the roles played
- Whether the person had any explicit training to support their role development/ mentoring
- How have previous experiences affected ability to do role, the skills/capacities needed
- Skills and capabilities you would still like to develop

4. What have you learnt about how to perform your role in the [name of partnership], the kinds of skills, knowledge and attitude that is needed? What has helped or hindered you?

For peer review only - http://bmjopen.bmj.com/site/about/guidelines.xhtml

Probe around:

- Particular knowledge that helps, e.g. learning about each other's org culture
- Particular skills that help or know-how, e.g. experience merging or balancing these perspectives and skills
- Relationships and developing a common language, purpose etc
- Other people being key, e.g. new roles crossing over org boundaries
- Infrastructure being key, e.g. meetings/forums for collaborating
- Support being key, e.g. training, mentoring, activities of CCP team

E. Developing and delivering the partnership: strategic and operational aspects

The aim of this section is to explore the ways in which the partnership is developing. This includes the initial setup, building trust and relationships, and individual level and partnership capabilities.

i) Understanding of the RPP approach and the current strategy for implementing it

The purpose of this section is to capture how the RPP approach is understood, and the goals and priorities for partnerships. It will be important to understand how the work programme is negotiated and agreed and changes as the partnerships develops.

Questions around understanding of the RPP approach and goals are for everyone but the strategy question is just for leadership / those involved in the decision-making

1. What were your first impressions of the RPP approach? Can you describe the RPP approach in your own words?
   Probe around:
   - Main aims and objectives
   - How similar the RPP idea is to their initial thinking about what the partnership might look like
   - What do they think is most important to get right?
   - What has influenced their understanding, e.g. previous experience, reading they have done, activities and literature from co-design team, implementation team
   - Whether they have learnt anything about this type of working, as they have started on the journey

For peer review only - http://bmjopen.bmj.com/site/about/guidelines.xhtml

2. Can you tell me what the goals are for the partnership from your perspective?
   Probe around:
   - How this has changed over time
   - Whether these goals are reflected in a strategy document or have emerged from strategic discussions
   - If there is a strategy document then ask for it if not already seen
   - What the reasons were for choosing these goals, e.g. unmet need, influence of policy / economic context, influence of key stakeholders etc
   - Whose interests were reflected in the goals, how they were negotiated

3. Do you have a main focus of work for [name of partnership] for the short term (e.g. next 6 months or so)? Can you talk me through the process through which your partnership decided on this focus/foci for the work?
   Probe around
   - Who was involved in the decision making?
   - Why this focus was chosen over others, e.g. gap/problem identified, other aspects contingent on this, quick win, advocated by a particular partner, met the needs of a particular stakeholder, influence of university or senior management at provider, or other external players?
   - Whether there were any differences in opinion and what they were about, how they were resolved
   - If there is more than one focus then explore how this is managed?
   - Try to get them to focus on aspects other than the research agenda, e.g. communications about partnership or more generally, capacity-building of staff, building relationships and trust, participation/addressing inequalities of power, bringing in missing voices

ii) Understanding how the partnership's plans will be delivered: structure, organisation and resources within the partnership

The purpose of this section is to capture how the RPP approach is being delivered, whether there is a plan and overarching framework/structure for delivery, whether people have specific roles, what activities are being carried out, by whom and with what resources. It will be important to understand how the partners contribute to explore the degree of participation and power relationships. We will also want to track how the goals and priorities for partnerships change as the partnership develops.<br><br>This does not need to be asked of everyone; mainly for leaders

1. Can you describe the plan for how you will deliver the partnership's work programme?

For peer review only - http://bmjopen.bmj.com/site/about/guidelines.xhtml

Probe around:

- Whether the plan is written down (ask for it to be shared)
- Whether the plan is long-term or short-term (e.g. next six months)
- What the content of the plan is: infrastructure building, capacity-building/career development, research agenda
- How the work is organised, e.g. workstreams or cycles for core partnership activities, research projects

2. Can you describe how you are managing the delivery of the work programme for the partnership?
   Probe around:
   - Governance structure, i.e. accountabilities, leadership, critical friends/oversight
   - Key functions and structures for delivery, e.g. communications, administration, finances
   - Leadership of the work
   - Development of new roles, allocation of roles and responsibilities for the different aspects of the work / is it more informal e.g. people volunteer
   - What is supporting people to work together / deliver the work programme, e.g. regular meetings,

3. Can you describe how you are resourcing the work programme for the partnership?
   Probe around:
   - Explore people – who is involved and which organisation they come from
   - Explore finance and where the money is coming from (e.g. CCP grant, partners own budgets, other sources)
   - Any other resources that need to be brought in, e.g. software/technical expertise, website etc

iii) Understanding the capacity of the partnership to deliver the work programme and intentions to build capacity

The aim of this section is to explore the way in which organisational capacity to conduct partnership work has built up over time.

The issues and themes to dig into are around whether the partnership has the right membership composition in term of skills and knowledge, whether the partnership feels cohesive and members identity with it are the individual level capabilities needed, the types of capabilities needed by different partners and how these are balanced. We are also interested in the relationship between individual level capabilities and the building of partnership level capabilities that can lead to a functioning and sustainable entity.

For peer review only - http://bmjopen.bmj.com/site/about/guidelines.xhtml

1. Thinking about the ability of the [name of partnership] to deliver its goals, what would you say are its current strengths and weaknesses?
   Probe around:
- Composition of the partnership? Knowledge, skills or roles missing?
- Sense of partnership being cohesive? Identity and shared language/values/mission etc
- Other resources for the partnership, e.g. sufficient time to dedicate, money, data availability/accessibility
- Strength of organisational capabilities/routines/infrastructure to support partnership activities rather than individuals, e.g. procedures and processes embedded in routines, robust to staff leaving
- Pacing – too fast or too slow

2. Are there plans to address the weaknesses? What do you think could be done to strengthen the partnership?

Probe:

- around whether they have formally assessed strengths and weaknesses
- written plan

3. Can you describe any factors that enable/inhibit [name of partnership] from building up its ability or capacity to deliver its goals?
   Probe around:
- Wider objectives, strategies and priorities within social care or HEI system
- Existing infrastructure, i.e. ENRICH, ARCs
- Organisational types, i.e. private vs local authority care homes
- Professional level/individual level factors
- Resources
- Communication and understanding

4. Do you have a sense of how your organisation and other organisations perceive the [name of partnership]? What do you think their perception is of the partnership and what is your opinion based on?
   Probe around:
   - Interest from other organisations
   - Recognition in local news, organisation meetings/news

F. Doing and using research for real-world change

For peer review only - http://bmjopen.bmj.com/site/about/guidelines.xhtml

This section explores the way in which research is being done and used by the partnership and the impact the partnership's research is having on practice more broadly. Key aspects to explore are power dynamics, the degree of participation in all aspects of the research by partnership members and how these activities are being supported by the partnership and routinised.

1. Can you describe the process through which you developed your research agenda? To what extent is there an established process for deciding the research agenda?

Probe around:

- What drove the decisions/source of ideas: problems of practice, other internal organisational factors, external factors, previous research done
- process: generating ideas, negotiating around ideas, developing questions, planning the research, including methods and timescales, resourcing the research
- routinisation: What forums did this take place? Are there standard processes?
- Explore role of power, interests balanced, how and to what extent people were involved in the process

2. Can you describe your research agenda? Are you satisfied with the agenda? What would you have preferred to be different? Do you think others feel the same way?

Probe around:

- The process for developing the agenda
- Whether there are specific projects, and the content of projects,
- The focus and nature of the research
- The quality of the research plan
- Considerations around time
- Involvement of different people in the planned research

3. I'd like to understand the research process in more depth, so can we choose two research projects and you can describe to me how these projects are progressing?
NOTE: Choose projects that are different either because they vary in size and scope, involve different people or different methods, have been more or less successful. Talk through each project separately covering the questions below to move from developing and doing the research, to interpreting and using the research.

4. First, can you describe to me how the research is being done?
Probe around:

- the type of research – methods and designs

For peer review only - http://bmjopen.bmj.com/site/about/guidelines.xhtml

- who is involved, to what extent, and how has the research been managed to get greater involvement
- Power dynamics: people involved, degree of participation in the process, who is leading, and attempts to change dynamics

5. Have you had to make any compromises when doing the research? What have these been and why did you make them? Who was involved in the decision?
   Probe around:
   - Types of compromise and how this was negotiated/decided: exclusion of people, types of methods, design and research quality
   - Internal drivers, e.g. time and resources; pacing issues related to skills, experience and expertise of partnership members; availability of data
   - External drivers, e.g. needs of management, HEI, policy environment, local concerns,

6. What was the output of the research? How have the findings been summarised and communicated?
   Probe around:
   - Whether outputs are more academic (e.g. reports and articles) or more practice oriented (guidelines, etc), or innovations
   - Methods of communication that crossed different org boundaries
   - The role of key individuals in this process and the role of leaders
   - The role of existing organisational communication pathways
   - Role of power- who controlled the communication process

7. What has the reaction of the partnership been to this research? Have there been discussions about it?

   Probe around:

   - How has the partnership made sense of the findings: formal and informal activities
   - What the focus of sensemaking was e.g. language, purpose, action, link to partnership goals
   - Who was involved in discussions, how were inputs from everyone managed and facilitated
   - Routinisation of sensemaking and research use: meetings, processes for considering research

For peer review only - http://bmjopen.bmj.com/site/about/guidelines.xhtml

8. Have there been any actions as a consequence of the research? Can you explain the ways in which you personally/[name of partnership]/[name of organisation] were influenced by the research findings?
   Probe around:
- In what forums/meetings/situations are decisions about changes to services/care/strategies made within care homes
- Changed priorities, agendas and solutions
- Co-design of materials/tools/service innovation, new processes/routines
- Explicitly making a decision based on evidence
- Justified previous decisions/whose interests did the use of research serve

9. Has the research been recognised by anyone who isn't part of the partnership or by any organisation that isn't part of the partnership? Have you tried to interest other people and organisations beyond the partnership in your work?
   Probe around:
   - Other academic institutions, providers, local authorities, NHS, funders, knowledge intermediaries
   - How other found out about it: channels of communication, key people, networks, deliberate strategies for spread
   - Strategies used: upscaling research projects, communication channels and materials, individuals/roles

10. Have you been satisfied with how this research project has gone/is going? What would you have preferred to be different? Do you think others feel the same way?

Probe around:

   - The process for doing the research
   - The quality of the research
   - Considerations around time and resources
   - Involvement of different people in the planned research
   - Process for sensemaking
   - Degree to which research was used, made a difference

11. Can you talk about what types of things made the research project difficult to deliver or helped the research to succeed in its aims?
    Probe around:
- Research approach/design used
- Time and resource to use research
- Organisational culture/pre-existing knowledge
- Leadership and communication pathways internal to care home

For peer review only - http://bmjopen.bmj.com/site/about/guidelines.xhtml

- [name of partnership] composition
- Political factors/local authority policy
- Wider HEI/care home/social care context

12. What, if anything, do you think the partnership has learnt from this research project about doing research that is relevant for practice and leads to real world change? How do you think that learning will be used to inform future projects?
    Probe around:
    - What types of things have been learnt, e.g. strategies for involvement, elevating power, ways of communicating, etc
    - What processes are in place to capture learning and ensure it informs future practice
    - Have processes changed as a result, have practices changed as a result

G. Monitoring, evaluation and learning

1. Have you thought about what success would look like for the partnership? Can you describe what success would look like, at one year, two years etc?

2. Do you have any processes in place for monitoring and evaluating what you are doing as a partnership? How are you using that data?
   Probe around:
   - What evidence / data is collected, by whom, e.g. just about research projects or also reflections on how they are working, what is working well / less well
   - Where evidence / data is from, e.g. CCP evaluation team, implementation team, their own data/ tools
   - How is evidence collected: formally through monitoring tools, evaluation, end of project reflection and learning
   - Used to celebrate success, demonstrate success to funders/partner organisations, learning and improvement

3. What do you think are the most important things you have learnt as a partnership? Can you give any examples of how the partnership has changed what it is doing in response to learning?
   Probe around:
   - Learning directly from outputs, e.g. informed practice
   - Changed ways of working to address problems, limitations, improve processes
   - Changed views about who should be involved and to what extent, at what point

For peer review only - http://bmjopen.bmj.com/site/about/guidelines.xhtml

- Changed perspective about potential for the partnership, e.g. changed view about the potential for research to inform practice, scope to have impact on partners and wider system

H. Successes and failures, barriers and facilitators

5. What would you say are your partnership's main achievements so far? What have you learnt from this and how will you take this forward into future partnership activities?

6. Is there anything that has gone less well for your partnership? What have you learnt from this and how will you take this forward into future partnership activities?

7. Have there been any challenges or has anything prevented your partnership making the progress you would have liked? How have these impacted on your partnership's progress?
   Probe around:
- Leadership
- Communication
- trust
- Resources
- Internal politics of partnership or organisation
- External factors, e.g. HEI, organisation, wider political / economic context, structures and wider priorities, CCP funding

8. Have you found anything, anyone or any organisation particularly helpful or supportive? How have these had a positive impact on your partnership's progress?
   Probe around:
   - CCP support offered from co-design or implementation
   - Support from partner organisations, e.g. contribution of resources, changing practices, encouragement
   - Support from other organisations, e.g. regional organisations
   - Peer network for partnership projects
   - Products / previous activities of your partnership

9. Can you describe the interactions you have had the with the co-design and the implementation team? What impact has this had on the partnership?
   Probe around:
   - Whether they made a request or were approached

For peer review only - http://bmjopen.bmj.com/site/about/guidelines.xhtml

- Types of help you received
- Types of questions you had/frequency
- Other support that would be useful

I. Concluding questions

1. Is there anything that we haven't covered that you would like to mention?

2. Are there any people or organisations that you work with that you think we should speak to as part of our research?

3. Are there any documents that you think we should look at as part of our research?

For peer review only

For peer review only - http://bmjopen.bmj.com/site/about/guidelines.xhtml

Stakeholder interview proforma

This proforma will be used for stakeholders connected to the three case study partnership sites. Stakeholders include anyone who has had influence over strategic direction and operations of one the partnership or is part of an organisation that has interest and could be directly influenced by partnership work. This could include university leadership, local authority leaders or commissioners, social workers, directors of care homes, local trade associations, CQC inspectors, local Applied Research Collaborations (ARCs) or other research collaborations, Enabling Research in Care Homes (ENRICH) members, or Clinical Research Networks. We need to adapt the proforma based on who we are speaking to, as not all of these questions will be relevant to all interviewees. As issues emerge, we may need to add questions in to ensure all relevant issues are covered.

The aim of the stakeholder interviews is to explore the way in which those external to the partnership but with a vested interest interact, use, and value the RPP.
In the early-stage interviews, we will focus on gaining insight into the types of organisations and key people who have an interest or strategic oversight of the operations of the partnership and contextual factors relating to their organisation and networks. Early interviews will also seek to gain insight on the level awareness and communication they have of partnership work.

In later interviews, we will look to explore more directly how stakeholders are making sense of partnership goals and research outputs, whether research is useful and useable, and whether the partnership is valuable from an external perspective.

J. Introductory information

My name is XXXXXX. Thank you for agreeing to take part in the Creating Care Partnerships (CCP) study.

We are interviewing stakeholders from the [name of partnership] lead to get a better understanding of your experiences of working with [name of partnership]. The interview should last an hour. During the interview, I will ask you about your level of involvement with the partnership, how you use and value the work produced by the partnership, and external events and local conditions influencing how the partnership works and its ability to achieve its goals.

I am sure there will be some areas you have more knowledge about. Where there are gaps in your knowledge it would be helpful if you could identify people or key documents that may help us to better understand the way your organisation works with the [name of partnership].

Informed Consent

For peer review only - http://bmjopen.bmj.com/site/about/guidelines.xhtml

4. Can I just check that you received the study information sheet and that you have had a chance to read it?

   No - [Review the study information sheet in detail]
   Yes - *Good* [Review the study information sheet briefly]

5. Do you have any questions about the wider study or about the interview we will be conducting today?

6. If you are happy to please can you sign the consent form that I sent you and email it back to me for our records. The consent form is a standard form used in social research and is used to ensure that:

- you understand the aims of the study
- you understand what your participation in the study will involve
- you are happy for the discussion to be audio recorded
- you know that you can change your mind about taking part in the study at any time – you can request for the discussion to stop at any time and if at any point you want to withdraw from the study you can request this by using the contact information provided to you today
- you know that your views will be kept confidential and your name will never be used in anything that is written about the study
- you consent to take part.

[Note: if the participant is not able to sign and return the consent form then consent can be recorded orally.]

[Start recording] Software will request participant to give permission to start recording. If face-to-face then request permission to start recording

K. Introductory questions

The aim of this section is to gain some contextual information about the stakeholder and their organisation, as well as the relationship between themselves/their organisation and the [name of partnership].

3. Can you tell me about your current professional role at [name of organisation] and how long you have been in post?

4. What's a typical day for you?

5. How would you describe your organisation? What is its purpose / mission? Probe around:
   - Organisational type
   - The area the organisation serves
   - Population it serves/demographic

For peer review only - http://bmjopen.bmj.com/site/about/guidelines.xhtml

L. Understanding of the partnership and the RPP approach

1. How were you first introduced to / come to hear about the [name of partnership]? First thoughts and impressions

2. Can you tell me about the relationship between yourself / your organisation and the [name of partnership]?
   Probe around:
   - Nature of relationship

3. How familiar are you with partnerships between research and practice? Is this partnership different to others you have seen, or other collaborations between researchers and people who work in social care organisations? Can you describe the ways in which it is different or similar?
   Probe around:
   - Do they perceive it as having a specific approach
   - Do they perceive an intention for real world change, equality, etc (see other principles)

4. What is your perception of what [name of partnership] is trying to achieve? What do you think its goals and ambitions are? Do you have any thoughts about how achievable these goals are?
   Probe around:
   - What they think might be barriers in the wider system
   - What they think might help the partnership
   - How well it fits in the system, its potential for social care and the production and use of evidence to inform and improve practice

M. Interaction with the [name of partnership]

The aim of this section is to understand more about the ways in which stakeholders are engaging within the [name of partnership]. If they are using the research then we will explore whether they are able to access and make sense of research findings and how this could be improved.

1. Can you describe the ways in which your organisation has worked with or alongside the [name of partnership]?

For peer review only - http://bmjopen.bmj.com/site/about/guidelines.xhtml

Probe around:

- Understanding the joint activities
- How communication takes place
- Whether there was a focus on doing or using research

Note: if there is a focus on doing and using research, explore this in more detail

2. Why did you decide to work with [name of partnership]?
   Probe around:
   - Understanding what the stakeholder brings / partnership brings to stakeholder, e.g. resources, people, different perspective, potential to scale research etc
   - Strategic alignment?

3. How have you found working with [name of partnership]? Have there been difficult points? What have you learnt? Would you like to work with them more?
   Probe around:
   - Points of contention/consensus
   - How have issues been resolved
   - Learning about doing and using research in different ways, learning about practice context
   - What they would like to continue working with them around

N. Exploring influence of [name of partnership] on stakeholders and vice-versa

The aim of this section is to understand the wider impact of [name of partnership] and the influence of stakeholders on the [name of partnership].

1. In what ways, if at all, has [name of partnership] had an influence on your organisation or the way you work?
   Probe around:
   - outputs of the partnership e.g. used as evidence to inform decision-making
   - partnership ways of working, e.g. copying partnership practices, innovations, new infrastructure etc
   - Change mindsets e.g. see a greater potential for research and evidence to be used to inform practice

2. Thinking specifically about the research that [name of partnership] is doing / has done, have you found the research valuable? Can you give an example of how you have used their research and the influence this has had on your organisation or the way you work?

For peer review only - http://bmjopen.bmj.com/site/about/guidelines.xhtml

Probe around:

- Research agenda and priorities are of wider interest or narrow
- Look for different uses of research evidence: Changes in ideas/priorities, Direct decisions, Further research, Drawn upon materials/tools/service innovations, Justifying existing decisions
- Influence may be in changing the way they think about the potential for using evidence

3. In what ways, if at all, has [name of partnership] had an influence on the way you or your organisation think about the potential for research to inform practice? Do you have any examples of how you have changed how you work or how others work?
   Probe around:
   - Impact on their ways of working
   - Impact on the wider system

4. Do you think your organisation has had an influence on the [name of partnership]? Can you describe how you have influenced the partnership?

5. In what ways, if at all, has the work conducted in the partnership built capacity within your organisation?
   Probe around:

- Research capacity
- Other skills and knowledge
- Knowledge exchange

O. Exploring the stakeholder's views about the [name of partnership] and its value

The aim of this section is to understand how the stakeholder views the [name of partnership] and whether they see it as successful or less successful.

6. From the perspective of your role/organisation, in what ways has the [name of partnership] been successful/unsuccessful? What could they do better?
   Probe around:
   - Has it changed the way you value the RPP approach?
   - What could be improved?

Note: if there is a focus on doing and using research, explore this in more detail

7. How would you describe the [name of partnership] as a whole?
   Probe around:

- Identity/coherence

For peer review only - http://bmjopen.bmj.com/site/about/guidelines.xhtml

- Purpose
- Value
- Infrastructure

8. Can you describe the main challenges presented by factors external to [name of partnership] to it being successful?
   Probe around:

- Funding/resources
- Local and national policy
- Wider priorities and strategies within the wider social care/HEI system

9. Can you think of anything that might help the [name of partnership] to be more successful or address any challenges it is facing?
   Probe around:

- Funding/resources
- Local and national policy
- Wider priorities and strategies within the wider social care/HEI system

For peer review only - http://bmjopen.bmj.com/site/about/guidelines.xhtml

Co-design team interview proforma

This proforma will be used for members of the co-design team within the Creating Care Partnerships (CCP) project.

P. Introductory information

My name is XXXXXX. Thank you for agreeing to take part in the Creating Care Partnerships (CCP) study.

We are interviewing members of the codesign team to get a better understanding of the role of the co-design team within the CCP project, how your view of the RPP approach has changed over time and the support you gave to the three partnership sites.

The interview should last an hour. During the interview I will ask you about your role within the co-design team, what types of events and activities were held for the first phase of codesign and how this informed your work going forward with the sites. I will also ask you about your work with the sites, the principles you employed, and your reflections on how the sites were working to build their partnership.

I am sure there will be some areas you have more knowledge about. Where there are gaps in your knowledge it would be helpful if you could identify people or key documents that may help us to better understand the role of the co-design element of the project in supporting the sites to develop their partnerships.

Informed Consent

7. Can I just check that you received the study information sheet and that you have had a chance to read it?

   No – [Review the study information sheet in detail]
   Yes – *Good* [Review the study information sheet briefly]

8. Do you have any questions about the wider study or about the interview we will be conducting today?

9. If you are happy to please can you sign the consent form that I sent you and email it back to me for our records. The consent form is a standard form used in social research and is used to ensure that:

- you understand the aims of the study
- you understand what your participation in the study will involve
- you are happy for the discussion to be audio recorded
- you know that you can change your mind about taking part in the study at any time – you can request for the discussion to stop at any time and if at any point you want to withdraw from the study you can request this by using the contact information provided to you today

For peer review only - http://bmjopen.bmj.com/site/about/guidelines.xhtml

- you know that your views will be kept confidential and your name will never be used in anything that is written about the study
- you consent to take part.

[Note: if the participant is not able to sign and return the consent form then consent can be recorded orally.]

[Start recording] Software will request participant to give permission to start recording. If face-to-face then request permission to start recording

Q. Introductory questions about co-design team members and the role of co-design in the CCP project

The aim of this section is to gain some contextual information about the co-design team member's role in relation to the Creating Care Partnerships (CCP) project.

6. Can you tell me about your current professional role at [name of organisation] and how long you have been in post?

   How were you first introduced to the RPP approach? First thoughts and impressions

- Can you tell me in your own words what the RPP approach is and what it is trying to achieve?

- Can you tell me in your own words what the role of co-design is in the CCP project?

Explore around:

- Your role in relation to the overall aims of the project
- Your role in relation to the other CCP project teams, i.e. implementation, evaluation, spread and sustainability, lived experience reference group
- Your role in relation to the three sites that are implementing the RPP approach
- Any differences in how you have approached and carried out co-design in the CCP project compared to other projects, e.g. steps/method, aims/goals, outputs

R. First phase of co-design

The aim of this section to explore the types of activities taking place in the first phase of co-design work, what was learnt about the RPP approach from this phase, and how this learning fed into the co-design work with the case study sites.

1. Can you describe the main aims of the first phase of co-design?

For peer review only - http://bmjopen.bmj.com/site/about/guidelines.xhtml

2. Can you describe the process of taking the insights from the co-design activities to develop an RPP approach that is suitable for the English care home context?
   Explore around:
- How you balanced pre-existing knowledge/insights from the literature with stakeholder insight
- How your thinking changed about what the RPP approach is

3. How do you think implementing the RPP approach will play out in the sites? How will it be influenced by or influence elements of social care/HEI context? What kinds of challenges will the sites face? Do you think any elements of the RPP approach will be more achievable?
   Note: discuss social care and HEI context separately

   Explore around different levels of the system:
   - sectoral/political
   - organisational
   - professional

   Explore around the influence in relation to the following areas:
   - Building trust and relationships within RPPs
   - Individual skills, knowledge, capacity for engaging in partnership work
   - Organisational capabilities for doing research for practice improvement
   - Organisational capabilities for using research for service and system improvement

S. Second phase of co-design

The aim of this section is to explore the types of activities taking place in the second phase of co-design work, what types of support the local sites needed and the challenges of developing RPPs within the local context.

1. Can you describe the main aims of the co-design phase with the sites?

2. Can you describe how you organised the co-design sessions and your decision-making around the structure and content of the different sessions?

Probe around:
- What type of co-design activities did you run?
- What principles did you follow during this phase?
- Adapting to time and resources available within the sites, and the attitudes of the sites

For peer review only - http://bmjopen.bmj.com/site/about/guidelines.xhtml

3. How have the sites responded to the co-design process and activities?
Note: discuss each site separately

Probe around:

- What challenges did they have/what seemed to come easier?
  - in relation to the co-design materials and the activities
  - in relation to their understanding of the RPP approach
  - in relation to their understanding of how to develop their partnership in a way that is consistent with the RPP approach
- How did the different members of the partnership work together and with the co-design team? Any differences in degree of participation? Were some people more vocal? Did people defer to particular members of the partnership?

4. Do you have any sense of how the sites have used/are using the co-design sessions to inform the way they are developing their partnership?
Note: discuss each site separately
Probe around:

- Any feedback from the sites / follow-up
- Any indications from discussions in the sessions
- Did they bring plans or start planning in the sessions?

5. Did you have any sense of how the site's local context might be influencing their engagement with the co-design work?
Note: discuss each site separately
Probe around:

- What they want to discuss?
- Ability to work with the co-design team or together?

6. You have said your approach to working with the sites is to be more facilitative, so the co-design team do not become an extension of the partnership at each site. How has that intention worked in practice?

Note: discuss each site separately
Probe around:

- Was there an evolution from front-loaded support to gradually stepping back?
- Have the sites approached you for additional support, e.g. where progress has stalled? How have you managed that process?
- How did you decide when to give support and when to step back?
- Have there been any really challenging moments? Can you describe what the issue was and how it played out?

For peer review only - http://bmjopen.bmj.com/site/about/guidelines.xhtml

CCP implementation team interview proforma

This proforma will be used for members of the implementation team within the Creating Care Partnerships (CCP) project.

T. Introductory information

My name is XXXXXX. Thank you for agreeing to take part in the Creating Care Partnerships (CCP) study.

We are interviewing members of the implementation team to get a better understanding of the role of the implementation team within the CCP project and support you have given the sites as they are developing their partnership.

The interview should last an hour. During the interview, I will ask you about your role in the implementation team, how you view the RPP approach and the influence of wider contextual factors on applying this approach within social care. I will also ask you about the activities you have carried out to support the sites and your reflections on how the sites are developing their partnership.

I am sure there will be some areas you have more knowledge about. Where there are gaps in your knowledge it would be helpful if you could identify people or key documents that may help us to better understand the implementation element of the project.

Informed Consent

10. Can I just check that you received the study information sheet and that you have had a chance to read it?

    No - [Review the study information sheet in detail]
    Yes - *Good* [Review the study information sheet briefly]

11. Do you have any questions about the wider study or about the interview we will be conducting today?

12. If you are happy to please can you sign the consent form that I sent you and email it back to me for our records. The consent form is a standard form used in social research and is used to ensure that:

- you understand the aims of the study
- you understand what your participation in the study will involve
- you are happy for the discussion to be audio recorded
- you know that you can change your mind about taking part in the study at any time – you can request for the discussion to stop at any time and if at any point you want to withdraw from the study you can request this by using the contact information provided to you today
- you know that your views will be kept confidential and your name will never be used in anything that is written about the study

For peer review only - http://bmjopen.bmj.com/site/about/guidelines.xhtml

- you consent to take part.

[Note: if the participant is not able to sign and return the consent form then consent can be recorded orally.]

[Start recording] Software will request participant to give permission to start recording. If face-to-face then request permission to start recording

U. Introductory questions about co-design team members and the role of implementation team in the CCP project

The aim of this section is to gain some contextual information about the implementation team member's role in relation to the Creating Care Partnerships (CCP) project. It is also to gain an understanding of how the implementation team view the RPP approach and the ways it will relate to the social care/HEI context.

1. Can you tell me about your current professional role at [name of organisation] and how long you have been in post?

2. Can you tell me in your own words what the role of the implementation team is in the CCP project?

Probe around:
- Your role in relation to the overall aims of the project
- Your role in relation to the other CCP project teams, i.e. co-design, evaluation, spread and sustainability, lived experience reference group
- Your role in relation to the three sites that are implementing the RPP approach

3. Can you tell me in your own words what the RPP approach is and what it is trying to achieve?

   Probe around:
   - First thoughts and impressions
   - Core principles
   - Whether your views on this have changed following co-design work

4. How do you think implementing the RPP approach will play out in the sites? How will it be influenced by or influence elements of social care/HEI context? What kinds of challenges will the sites face? Do you think any elements of the RPP approach will be more achievable?
   Note: discuss social care and HEI context separately

   Explore around different levels of the system:
   - sectoral/political
   - organisational
   - professional

For peer review only - http://bmjopen.bmj.com/site/about/guidelines.xhtml

Explore around the influence in relation to the following areas:
- Building trust and relationships within RPPs
- Individual skills, knowledge, capacity for engaging in partnership work
- Organisational capabilities for doing research for practice improvement
- Organisational capabilities for using research for service and system improvement

C. Main activities and ways of working with the sites

The aim of this section is to explore the types of activities that the implementation team are engaged in to support the sites, the main challenges, and the impact.

1. Can you describe the main aims of the implementation work you are doing with the sites?
   Probe around whether there are any differences across the sites

2. Can you tell me about the activities you have been involved in to support sites and the main aims of these activities?
   Probe around whether there are any differences across the sites and around the following areas:
- Infrastructure support
- Research skills
- Agenda setting
- Communication skills
- Building relationships/managing conflict

3. Can you tell me about the types of support that the sites have asked for?
   Probe around whether there are any differences across the sites and around the following areas:
- Types of questions
- Formal/informal support
- Important/least important support needed
- Difference in support needs depending on academic/care home partners

4. How are the sites responding to the support you have given?

Note: discuss each site separately
Probe around:
- Your experiences of how receptive the sites are to suggestions and ideas
- How sites approach their interactions with you, i.e. collaborative, antagonistic, distant
- Difference between partners in how much they engage
- What challenges did they have/what seemed to come easier?
  - in relation to the co-design materials and the activities

For peer review only - http://bmjopen.bmj.com/site/about/guidelines.xhtml

- in relation to their understanding of the RPP approach
- in relation to their understanding of how to develop their partnership in a way that is consistent with the RPP approach

5. Can you describe how the local partnership context is influencing the type of support they need?
   Note: discuss each site separately
   Probe around:
- Local geography/particular needs of the population served
- Composition of partnership
- Care home type
- Existing infrastructure

6. Do you have any sense of how the sites have used/are using the implementation support to inform the way they are developing their partnership?
   Note: discuss each site separately
   Probe around:
- Any feedback from the sites / follow-up
- Any indications from discussions in the sessions
- Did they bring plans or start planning in the sessions?

7. You have said your approach to working with the sites is to be more facilitative, so the implementation team do not become an extension of the partnership at each site. How has that intention worked in practice?
   Note: discuss each site separately
   Probe around:
   - Was there an evolution from front-loaded support to gradually stepping back?
   - Have the sites approached you for additional support, e.g. where progress has stalled? How have you managed that process?
   - How did you decide when to give support and when to step back?
   - Have there been any really challenging moments? Can you describe what the issue was and how it played out?

D. Reflections on how the partnerships are working in practice

The aim of this section is to understand how the implementation team perceive the way in which partnership sites are working together, their ability to assess their own needs, and how contextual factors may be influencing their success.

1. Can you describe how partnership members are working together?
   Note: discuss each site separately
   Probe around:
   - Ways in which trust and relationships are building
   - Power dynamics between the partners, are some members more dominant than others?

For peer review only - http://bmjopen.bmj.com/site/about/guidelines.xhtml

- Whether partnerships have developed intentional strategies to manage unequal power dynamics

2. How well do the sites understand the RPP approach? What types of strategies are they putting in place to implement the approach?
   Note: discuss each site separately
   Probe around:
   - Strategies for conducting research to meet the problems of practice
   - Level of understanding and strategies for setting organisational and broader goals
   - Building capacity for joint boundary work
   - Focus on organisational goals vs wider system impact
   - Variation between different types of partners

3. How well are sites able to identify problems and the support they need?
   Note: discuss each site separately
   Probe around:
   - Ability to understand the knowledge, expertise & skills of different partners
   - Overcoming differences of opinion or ensuring the right mix of voices are there

4. In your opinion do the sites need more or different types of support to what has been identified? Are there problems within the partnership that have not been raised?
   Note: discuss each site separately
   Probe around:
   - Organisational contextual factors
   - Partnership composition
   - Partnership internal dynamics

5. In what ways do you think the sites have been affected by the social care/HEI context, and how has this affected their success in implementing the RPP approach?
   Note: discuss each site separately, and discuss social care & HEI context separately

For peer review only - http://bmjopen.bmj.com/site/about/guidelines.xhtml

Lived experience lead interview proforma

This proforma will be used for the Lived Experience Reference Group (LERG) of the Creating Care Partnerships (CCP) project.

V. Introductory information

My name is XXXXXX. Thank you for agreeing to take part in the Creating Care Partnerships (CCP) study.

We are interviewing the lived experience reference group lead to get a better understanding of the role of the lived experience reference group within the CCP project and the support the group has given the sites as they are developing their partnership.

The interview should last an hour. During the interview, I will ask you about the role of the lived experience reference group, how you view the RPP approach and the influence of wider contextual factors on applying this approach within social care. I will also ask you about the activities the group has carried out to support the CCP sites and your reflections on how the sites are developing their partnership.

I am sure there will be some areas you have more knowledge about. Where there are gaps in your knowledge it would be helpful if you could identify people or key documents that may help us to better understand the lived experience element of the project.

Informed Consent

13. Can I just check that you received the study information sheet and that you have had a chance to read it?

    No - [Review the study information sheet in detail]
    Yes - *Good* [Review the study information sheet briefly]

14. Do you have any questions about the wider study or about the interview we will be conducting today?

15. If you are happy to please can you sign the consent form that I sent you and email it back to me for our records. The consent form is a standard form used in social research and is used to ensure that:

- you understand the aims of the study
- you understand what your participation in the study will involve
- you are happy for the discussion to be audio recorded
- you know that you can change your mind about taking part in the study at any time – you can request for the discussion to stop at any time and if at any

For peer review only - http://bmjopen.bmj.com/site/about/guidelines.xhtml

point you want to withdraw from the study you can request this by using the contact information provided to you today
- you know that your views will be kept confidential and your name will never be used in anything that is written about the study
- you consent to take part.

[Note: if the participant is not able to sign and return the consent form then consent can be recorded orally.]

[Start recording] Software will request participant to give permission to start recording. If face-to-face then request permission to start recording

W. Introductory questions about the lived experience reference group and the role of the group in the CCP project

The aim of this section is to gain some contextual information about the LERG's role in relation to the Creating Care Partnerships (CCP) project. It is also to gain an understanding of how the LERG lead views the RPP approach and the ways it will interact with the social care/HEI context.

5. Can you tell me about your current professional role at [name of organisation] and how long you have been in post?

6. How were you first introduced to the RPP approach? First thoughts and impressions

7. Can you tell me in your own words what the RPP approach is and what it is trying to achieve?
   Explore around:
   - Core principles
   - Whether your views on this have changed following co-design work

8. Can you tell me in your own words what the role of the lived experience reference group within the CCP project?

Explore around:
- How members were recruited and what you were looking for
- The group's role in relation to the overall aims of the project
- The group's role in relation to the other CCP project teams, i.e. co-design, evaluation, implementation, spread and sustainability
- The group's role in relation to the three sites that are implementing the RPP approach
- What's not within their remit

9. How do you think implementing the RPP approach will play out in the sites? How will it be influenced by or influence elements of social care/HEI context?

For peer review only - http://bmjopen.bmj.com/site/about/guidelines.xhtml

What kinds of challenges will the sites face? Do you think any elements of the RPP approach will be more achievable?
Note: discuss social care and HEI context separately
Explore around different levels of the system:

- sectoral/political
- organisational
- professional

Explore around the influence in relation to the following areas:

- Building trust and relationships within RPPs
- Individual skills, knowledge, capacity for engaging in partnership work
- Organisational capabilities for doing research for practice improvement
- Organisational capabilities for using research for service and system improvement

C. Main activities and ways of working with CPP teams and the sites

The aim of this section is to explore the types of activities that the LERG are taking part in to support the sites, the challenges of this, and the impact.

8. Can you tell me about the activities you have been involved in to support the sites?
   Note: discuss the sites separately, if applicable
   Probe around:

- Site specific feedback on development of partnership, their research etc
- Indirect support to sites through working with the co-design or implementation team to make activities accessible, etc

9. Can you tell me about the types of support that the sites have asked for or the wider CCP team to support their work with the sites?
   Note: discuss the sites separately, if applicable
   Probe around:

- Types of issues
- Difficulties with any requests

10. Can you describe the benefit/value of the work the group has done so far?
    Note: discuss the sites separately, if applicable
    Probe around:

- Ways in which advice was received and incorporated
- Times when it has been less valuable
- Additional ways that the group could assist the sites or CCP team members in working with the sites

For peer review only - http://bmjopen.bmj.com/site/about/guidelines.xhtml

11. How are the sites responding to the support you have given?
Note: this question relates just to the sites so as to avoid evaluating CCP colleagues
Probe around:
- Your experiences of how receptive the sites are to suggestions and ideas
- How sites approach their interactions with you, i.e. collaborative, antagonistic, distant
- Difference between partners (care home/academic) in how much they engage

12. Can you describe any challenges faced by the lived experience reference group?
Probe around:
- Occasions when feedback was resisted or not taken on board
- Skills or specialisms of LERG members

13. Can you describe how the local partnership context influenced the type of support the sites needed?
Note: discuss each site separately
Probe around:
- Local geography/particular needs of the population served
- Composition of partnership
- Care home type
- Existing infrastructure and lived experience input

14. Can you describe how the RPP approach influenced the type of support the LERG provided to the sites/the wider CCP project team to support the sites?
Note: discuss the sites separately, if applicable
Probe around:
    - Core principles of RPP
    - Idea that it is based on bottom-up development
    - Ways this may differ to providing lived experience advice on other research projects

E. Reflections on how the partnerships are working in practice

The aim of this section is to understand how the lived experience lead perceives the way in which the sites are working with residents and family members, their ability of the sites to assess their own needs and how contextual factors may be influencing their success.

6. Can you describe how partnership members are working with care home residents and their families/friends?
Note: discuss each site separately

For peer review only - http://bmjopen.bmj.com/site/about/guidelines.xhtml

Probe around:

- Ways in which trust and relationships are building
- Power dynamics between the partners, are some members more dominant than others?
- Whether partnerships have developed intentional strategies to manage unequal power dynamics

7. How well were sites able to identify problems and what lived experience support they needed?
   Note: discuss each site separately
   Probe around:
   - Ability to assess where they were missing lived experience input and the consequences this was having

8. In your opinion what types of lived experience support do the sites need but have not identified? What are the barriers to this?
   Note: discuss each site separately
   Probe around:
   - Organisational contextual factors
   - Partnership composition
   - Partnership internal dynamics
   - Factors which relate to the social care context

For peer review only - http://bmjopen.bmj.com/site/about/guidelines.xhtml
