# Supplementary material for: Theory-based evaluation of three research–practice partnerships designed to deliver novel, sustainable collaborations between adult social care research and practice in the UK: a research protocol for a ‘layered’ contributions analysis and realist evaluation
Source: BMJ Open. 2022 Nov 25;12(11):e068651. doi: 10.1136/bmjopen-2022-068651 (PMC9703321; doi:10.1136/bmjopen-2022-068651)
Supplement: Reviewer comments [file bmjopen-2022-068651.reviewer_comments.pdf]

PEER REVIEW HISTORY

BMJ Open publishes all reviews undertaken for accepted manuscripts. Reviewers are asked to complete a checklist review form (**http://bmjopen.bmj.com/site/about/resources/checklist.pdf**) and are provided with free text boxes to elaborate on their assessment. These free text comments are reproduced below.

ARTICLE DETAILS

**TITLE (PROVISIONAL)**	A theory-based evaluation of three Research Practice Partnerships designed to deliver novel, sustainable collaborations between adult social care research and practice in the UK: a research protocol for a ‘layered’ contributions analysis and realist evaluation
**AUTHORS**	Malley, Juliette; Bauer, Annette; Boaz, Annette; Kendrick, Hannah; Knapp, Martin

This article was not externally reviewed at BMJ Open. Protocols that have been independently assessed prior to submission to BMJ Open are usually fast-tracked to publication on the grounds that further substantial changes will not be possible. This independent assessment will usually be external, independent review for both a substantial grant award from a non-commercial or government funder and ethics approval.
